# Multiagency programs with police as a partner for reducing radicalisation to violence

**DOI:** 10.1002/cl2.1162

**Published:** 2021-05-05

**Authors:** Lorraine Mazerolle, Adrian Cherney, Elizabeth Eggins, Lorelei Hine, Angela Higginson

**Affiliations:** ^1^ School of Social Science, St. Lucia Campus University of Queensland St. Lucia Queensland Australia; ^2^ School of Justice, Gardens Point Campus Queensland University of Technology Brisbane Queensland Australia

## Abstract

**Background:**

Multiagency responses to reduce radicalisation often involve collaborations between police, government, nongovernment, business and/or community organisations. The complexities of radicalisation suggest it is impossible for any single agency to address the problem alone. Police‐involved multiagency partnerships may disrupt pathways from radicalisation to violence by addressing multiple risk factors in a coordinated manner.

**Objectives:**

1. Synthesise evidence on the effectiveness of police‐involved multiagency interventions on radicalisation or multiagency collaboration

2. Qualitatively synthesise information about *how* the intervention works (mechanisms), intervention *context* (moderators), implementation factors and economic considerations.

**Search Methods:**

Terrorism‐related terms were used to search the Global Policing Database, terrorism/counterterrorism websites and repositories, and relevant journals for published and unpublished evaluations conducted 2002–2018. The search was conducted November 2019. Expert consultation, reference harvesting and forward citation searching was conducted November 2020.

**Selection Criteria:**

Eligible studies needed to report an intervention where police partnered with at least one other agency and explicitly aimed to address terrorism, violent extremism or radicalisation. Objective 1 eligible outcomes included violent extremism, radicalisation and/or terrorism, and multiagency collaboration. Only impact evaluations using experimental or robust quasi‐experimental designs were eligible. Objective 2 placed no limits on outcomes. Studies needed to report an empirical assessment of an eligible intervention and provide data on mechanisms, moderators, implementation or economic considerations.

**Data Collection and Analysis:**

The search identified 7384 records. Systematic screening identified 181 studies, of which five were eligible for Objective 1 and 26 for Objective 2. Effectiveness studies could not be meta‐analysed, so were summarised and effect size data reported. Studies for Objective 2 were narratively synthesised by mechanisms, moderators, implementation, and economic considerations. Risk of bias was assessed using ROBINS‐I, EPHPP, EMMIE and CASP checklists.

**Results:**

One study examined the impact on vulnerability to radicalisation, using a quasi‐experimental matched comparison group design and surveys of volunteers (*n* = 191). Effects were small to medium and, aside from one item, favoured the intervention. Four studies examined the impact on the nature and quality of multiagency collaboration, using regression models and surveys of practitioners. Interventions included: alignment with national counterterrorism guidelines (*n* = 272); number of counterterrorism partnerships (*n* = 294); influence of, or receipt of, homeland security grants (*n* = 350, *n* = 208). Study findings were mixed. Of the 181 studies that examined mechanisms, moderators, implementation, and economic considerations, only 26 studies rigorously examined mechanisms (*k* = 1), moderators (*k* = 1), implementation factors (*k* = 21) or economic factors (*k* = 4).

All included studies contained high risk of bias and/or methodological issues, substantially reducing confidence in the findings.

**Authors' Conclusions:**

A limited number of effectiveness studies were identified, and none evaluated the impact on at‐risk or radicalised individuals. More investment needs to be made in robust evaluation across a broader range of interventions.

Qualitative synthesis suggests that collaboration may be enhanced when partners take time to build trust and shared goals, staff are not overburdened with administration, there are strong privacy provisions for intelligence sharing, and there is ongoing support and training.

## 1 PLAIN LANGUAGE SUMMARY

### 1.1 Limited evidence for police‐involved multiagency partnerships that seek to reduce radicalisation to violence

Multiagency partnerships involving police are often implemented to foster collaboration and reduce radicalisation to violence. There is no clear evidence to support this approach, although a small number of studies provide mixed evidence about the effectiveness of multiagency partnerships for improving collaboration. Some studies offer insights about the costs and ways to best implement multiagency programmes.

### 1.2 What is this review about?

Police multiagency responses to violent extremism aim to reduce radicalisation to violence by fostering collaboration and partnering with other governmental agencies, private businesses, community organisations, or service providers. Police can play a central role in these partnerships because they are often one of the first points of contact with individuals who have radicalised to extremism.

**What is the aim of this review?**

This Campbell systematic review examines the processes and impact of police‐involved multiagency partnerships that aim to address terrorism, violent extremism, or radicalisation to violence. The review summarises evidence from five studies that met the impact review criteria and 26 studies that were qualitatively synthesised to explore the processes of multiagency collaboration.

### 1.3 What studies are included?

This review includes studies that evaluated either the processes or impacts of programmes that involve police acting in partnership with at least one other agency and that were aimed at reducing terrorism, violent extremism or radicalisation to violence.

The systematic search identified 7384 potential studies, of which five assessed the effectiveness of police‐involved multiagency interventions. A total of 181 studies examined how the intervention might work (mechanisms), under what context or conditions the intervention operates (moderators), the implementation factors and economic considerations. Of the 181 studies, 26 studies met the threshold for in‐depth qualitative synthesis to more comprehensively understand the mechanisms, moderators, implementation and economic considerations for police‐involved multiagency interventions.

### 1.4 What are the findings of this review?

There is not enough evidence to assess whether these programmes work to reduce radicalisation to violence. Only one study assessed the impact of a police‐involved multiagency partnership on radicalisation to violence. This study evaluated the World Organisation for Resource Development Education (WORDE) programme, a Muslim community‐based education and awareness programme involving police in some components.

#### 1.4.1 Do multiagency programmes that aim to reduce radicalisation to violence improve collaboration?

There is a small amount of mixed evidence regarding whether these programmes can work to improve collaborations between agencies. Four studies met the inclusion criteria to assess the impact of a police multiagency partnership on interagency collaboration. The first study examined the impact of agency alignment with a Target Capabilities List (TCL). The evidence from this study showed that greater alignment with the TCL was associated with better working relationships, more intelligence sharing, and more engagement with the U.S. Federal Bureau of Investigation (FBI), other law enforcement agencies, and fusion centres.

The second study assessed whether the number of multiagency collaborative partners influenced perceptions of clarity and understanding of the strategies and goals of organisations at three levels. Evidence from this study suggests that a larger number of collaborative partners is associated with better understandings of missions, responsibilities and goals at the state and local/departmental level, but not at the federal level, where more partners is associated with less understanding.

The third and fourth studies both examined the impact of grants from the U.S. Department of Homeland Security (DHS). One study found a negative direct relationship between the perceptions of the influence of DHS grants, and homeland security preparedness. The final study found that the receipt of DHS funding did not significantly predict whether or not an agency engaged in at least one form of homeland security innovation.

#### 1.4.2 What processes facilitate or constrain implementation of this intervention?

Twenty‐six studies met our threshold for more thorough examination of the processes that facilitate or constrain implementation, as well as providing information about the costs and benefits of the programme. Some themes that emerged include the importance of taking time to build trust and shared goals among partners; not overburdening staff with administrative tasks; targeted and strong privacy provisions in place for intelligence sharing; and access to ongoing support and training for multiagency partners.

### 1.5 What do the findings of this review mean?

There is limited and mixed evidence about the processes and impact of police‐involved multiagency programs aimed at reducing radicalisation to violence. Only five initiatives so far have been evaluated for effectiveness, and with low quality methods. A larger number of studies (181) provide insights in the context, functioning and cost effectiveness of police‐involved multiagency initiatives, with 26 higher‐quality studies synthesised in‐depth. Future research should aim to rigorously evaluate the outcomes of such initiatives.

### 1.6 How up‐to‐date is this review?

The review authors searched for studies conducted between January 2002 and December 2018.

## 2 BACKGROUND

### 2.1 The problem, condition or issue

Violent radicalisation is a complex problem, complicated by the lack of a clear terrorist profile and variation in the risk factors that predict violent extremism across individuals and groups (Campelo et al., 2018; Carlsson et al., 2020; Desmarais et al., 2017; Wolfowicz et al., 2019). While models of understanding radicalisation vary (Borum, 2015; Christmann, 2012; Desmarais et al., 2017; Horgan, 2008; Koehler, 2017; Kruglanski et al., 2019; Sarma, 2017), it is broadly defined as the process by “which a person adopts extremist views and moves towards committing a violent act” (Hardy, 2018, p. 76; Irwin, 2015; Jensen et al., 2018). Radicalisation has been linked with individual and group engagement in terrorist attacks against innocent civilians (Wilner & Dubouloz, 2010), as well as individuals entering conflict zones to join formal extremist groups to engage in violent combat (Lindekilde et al., 2016). As a result, radicalisation has become a key focus for counterterrorism and violence prevention interventions.

The complex and varied nature of individuals' progression from radicalisation to violence presents challenges for designing and evaluating appropriate interventions and policy responses (Hafez & Mullins, 2015; Helmus et al., 2017; Horgan, 2008; Horgan & Braddock, 2010; Jensen et al., 2018; Kruglanski et al., 2019). This level of complexity has driven national counterterrorism policy agendas to adopt intersectoral and multiagency responses that aim to address various radicalisation processes and risks (Beutel & Weinberger, 2016). These multiagency responses often involve partnerships and collaborations between various different agencies and entities (Hardy, 2018), such as governmental agencies, private businesses, community organisations, and service providers.

Multiagency interventions can provide a framework for pooling and sharing resources to address a common problem (Crawford, 1999; Rosenbaum, 2002), such as radicalisation to violence. Yet they can be challenging to implement, and their effectiveness may be influenced by the quality and nature of the collaboration between agencies (see Berry et al., 2011 for review; Atkinson, 2019; Gittell, 2006; Kelman et al., 2013; McCarthy & O'Neill, 2014; Rosenbaum, 2002). Multiagency interventions may be conceptualised on a continuum, with activities ranging from minimal collaboration to a wholistic integration of agencies and organisations (Atkinson, 2019). As a result, the outcomes of multiagency interventions may vary depending on where the intervention falls—or is perceived to fall—on this collaborative continuum (Atkinson, 2019). Partnerships can enhance formal and informal communication, trust, respect, shared goals, and knowledge (Bond & Gittell, 2010). Conversely, partnership‐based interventions may highlight a number of shortcomings in service delivery including the disjointed nature of services, the need for significant stakeholder buy‐in, the isolation for some of the organisations or individuals, and the resource‐intensive nature of many of these collaborations (Atkinson, 2019; Bond & Gittell, 2010; Crawford, 1999; McCarthy & O'Neill, 2014; Youansamouth, 2019). There is also the possibility that multiagency approaches could lead to adverse outcomes (Galloway, 2017; Norton, 2018). For example, multiagency responses that have poor levels of coordination and communication could lead to cases falling through the cracks where no one agency responds under the misguided assumption that another partner agency is taking the lead (Richards, 2017; Smith et al., 1992). Ransley (2016) also raises the possibility of coercion from multiagency responses. Therefore, when assessing the effectiveness and the intended outcomes of multiagency interventions, it is also important to consider the context, potential backfire effects, and quality of the processes underpinning multiagency collaboration.

A broad range of agencies and experts can be involved in multiagency approaches for reducing radicalisation to violence or violent extremism (Weine et al., 2017). Nevertheless, the police are often one of the first points of contact with individuals who have radicalised to extremism. The police are also the first point of call for those who are concerned about or report known associates, friends or family members as being at‐risk of radicalisation. As such, police are important partners for identifying, reducing and building resilience to radicalisation (Cherney, 2015). This review will, therefore, focus on the effectiveness of police‐involved multiagency interventions for reducing radicalisation to violence and improving multiagency collaboration.

### 2.2 The intervention

Multiagency interventions are characterised by two or more entities partnering to solve a shared problem. These entities may be government agencies (such as education, immigration, customs, home affairs, employment, housing, health), or nongovernmental agencies, including: local councils; businesses; community organisations (such as churches, mosques and other houses of worship) and service providers (such as resettlement agencies, local health providers). This review included any multiagency intervention, where at least one of those partners is the police and where the intervention explicitly aims to address terrorism, violent extremism or radicalisation to violence. This type of intervention can include a range of approaches, including: police engaging with different community and agency stakeholders to help identify terrorist threats (Innes et al., 2011; Ramiriz et al., 2013); police working with other agencies to refer, assess, or case‐manage individuals convicted of terrorism or identified as at‐risk for radicalisation (Cherney & Belton, 2019); or police forming task forces or partaking in regular structured meetings with other agencies to problem‐solve issues pertaining to radicalisation or extremism (Koehler, 2016).

### 2.3 How the intervention might work

Some observe that there is a great deal of heterogeneity in the risk factors and triggers for radicalisation (Dalgaard‐Nielsen, 2010, 2018; Horgan, 2009). This means there is a variety of risk and background factors that may lead an individual (or a group of individuals) to radicalise to violent extremism (Campelo et al., 2018; Carlsson et al., 2020; Vergani et al., 2018). Research also demonstrates the complex nature of different progression pathways from radicalisation to violence (Horgan, 2008; Kruglanski et al., 2019, 2020). The literature is, therefore, in agreement that the complex nature of radicalisation risk and pathway processes to violence makes it difficult for any single agency, organisation or entity to address the problem alone (Dalgaard‐Nielsen, 2018). As such, interventions to address the problem of radicalisation to violence are often characterised by multiagency partnerships, working across different service delivery sectors (see e.g., Cherney & Belton, 2019; Innes et al., 2011).

Multiagency partnerships may disrupt pathways from radicalisation to violence by collectively addressing multiple risk factors in a holistic and coordinated manner (Butt & Tuck, 2014). The multiagency approach to tackling violent extremism may be effective because it fosters a coordination of effort (Kelman et al., 2013), draws from a broad range of expertise (Crawford, 1999), allows for information and intelligence sharing (Cherney, 2018; Murphy, 2008; Slayton, 2000), and enables the pooling of resources (Crawford, 1999; Sestoft et al., 2017).

El‐Said (2015) describes a range of different ways that multiagency partnerships operate: by formal and informal arrangements, such as legislative or regulatory frameworks, memoranda of understanding or policy standards stipulating channels for information sharing or better interpersonal relations between agencies (see also Koehler, 2016). These arrangements create opportunities for referrals being made from various sources (Koehler, 2017), increasing the capacities for partnerships to detect and respond to those at early pathways to radicalisation and violence. The capacities of multiagency partnerships to better detect and respond to problems over and above what is possible by agencies or entities working alone are enhanced through better information sharing and referral processes (Cherney, 2018; Murphy, 2008; Slayton, 2000). Partnerships can enhance programme planning and design so that counter radicalisation strategies address the required risks and vulnerabilities amongst individuals and groups (Koehler, 2017). The range of expertise across multiagency partners also help to enhance programme implementation by ensuring that all required components of a strategy are delivered (Crawford, 1999). They are likewise important in relation to programme evaluation by enabling the sharing of data that can be used to assess programme effectiveness (Cherney, 2018).

### 2.4 Why it is important to do the review

Police cannot tackle the problem of radicalisation, violent extremism, and terrorism on their own (Cherney & Hartley, 2017). Many of the risk factors for radicalisation and violent extremism are complex (Dawson et al., 2016; Hafez & Mullins, 2015; Kruglanski et al., 2019, 2020). Research suggests that it is not just the presence of risk factors, but rather the accumulation of risk factors (Campelo et al., 2018; Carlsson et al., 2020; Simi et al., 2016) and what Vergani et al. (2018) describe as the push, pull and personal nature of the radicalisation process. The complexity of the process, therefore, can trigger a range of different vulnerabilities. Some of these vulnerabilities relate to a lack of sense of belonging (Harris‐Hogan, 2014), which requires different institutional responses spanning the family, educational and work context, all of which contribute to the formation of a sense of identity (Kruglanski et al., 2019).

The complexity and variability of the radicalisation process provides an opportunity for police to partner with various agencies and community groups to tackle radicalisation in a multifaceted manner. As such, multiagency interventions have become an important approach to tackle the problem of radicalisation and violent extremism (Butt & Tuck, 2014; Mucha, 2017; Sestoft et al., 2017). Existing evidence, however, does not provide a clear understanding of the effectiveness of police‐involved, multiagency approaches to radicalisation (Cherney & Hartley, 2017; Koehler, 2017; MacDonald, 2002). In addition, there are no existing reviews of multiagency programs, with police as partner, for addressing radicalisation to violence.^1^ Given the cost of forming multiagency interventions and the organisational complexities of managing and maintaining these types of responses, it is imperative to know whether current multiagency approaches that include police partners are effective for reducing radicalisation to violence and enhancing multiagency collaboration. Policy makers, practitioners, and researchers also need to understand not only whether the intervention works, but also how the intervention works (mechanisms), under what conditions or contexts (moderators), and what the implementation considerations and cost implications are.

This review aims to fill a significant gap in the evidence‐based literature for countering violent extremism in two ways. First, by quantitatively synthesising the existing evidence for the impact of multiagency police‐involved programs on violent radicalisation or multiagency collaboration. Second, by qualitatively synthesising research that reports on the mechanisms, moderators, implementation considerations, and economic information pertaining to police‐involved multiagency programs that aim to counter radicalisation to violence. The results from this review will inform future decision‐making regarding the design and evaluation of multiagency programs by synthesising the evidence for their effectiveness, identifying potential gaps in the evidence‐base, and providing insight into what level of investment is required for the implementation and evaluation of primary studies.

## 3 OBJECTIVES

The first objective of this review (Objective 1) is to answer the question: how effective are police‐involved multiagency interventions at reducing radicalisation to violence or improving multiagency collaboration? As part of this objective, the review also aimed to ascertain if the effectiveness of police‐involved multiagency interventions varies by geographical location, target population, nature of the intervention approach (e.g., number of components, specific intervention techniques), and number and type of multiagency partners. The second objective of this review (Objective 2) is to qualitatively synthesise pertinent information about *how* police‐involved multiagency interventions for countering radicalisation to violence might work (mechanisms), under what *context* or conditions (moderators), the implementation factors, and economic considerations.

## 4 METHODS

### 4.1 Criteria for considering studies for this review

#### 4.1.1 Types of studies

To fulfil the objectives of this review, two types of studies will be included. The specific type of studies used to address each review objective may overlap, and are detailed in the subsections below.

##### Types of study designs for review of effectiveness (Objective 1)

To be included in the review of effectiveness (Objective 1), a study needed to be a quantitative impact evaluation that employed a randomised experimental (e.g., RCT) or a quasi‐experimental design with a comparison group that does not receive the intervention. Eligible comparison groups were: “business‐as‐usual” treatment, no intervention, or an alternative intervention (treatment‐treatment designs).

Rigorous quasi‐experimental studies can also be used to estimate causality, particularly when the research design includes strategies to minimise threats to internal validity (see Farrington, 2003; Shadish et al., 2002). Strategies for reducing threats to internal validity may include: controlling case assignment to treatment and comparison groups (regression discontinuity), matching characteristics of the treatment and comparison groups (matched control), statistically accounting for differences between the treatment and comparison groups (designs using multiple regression analysis), or providing a difference‐in‐difference analysis (parallel cohorts with pre‐ and posttest measures). The following “strong” quasi‐experimental designs were eligible for this review:

- Cross‐over designs
- Regression discontinuity designs
- Designs using multivariate controls (e.g., multiple regression)
- Matched control group designs with or without preintervention baseline measures (propensity or statistically matched)
- Unmatched control group designs without preintervention measures where the control group has face validity
- Unmatched control group designs with pre‐post intervention measures which allow for difference‐in‐difference analysis
- Short interrupted time‐series designs with control group (<25 preintervention and 25 postintervention observations (Glass, 1997)
- Long interrupted time‐series designs with or without a control group (≥25 preintervention and postintervention observations (Glass, 1997)
  Less rigorous quasi‐experimental designs can be used to illustrate the magnitude of the relationship between an intervention and an outcome, yet have limitations for establishing causality. Therefore, we excluded the following weaker quasi‐experimental designs in the synthesis of intervention effectiveness:
- Raw unadjusted correlational designs where the variation in the level of the intervention is compared to the variation in the level of the outcome; and
- Single group designs with pre‐ and postintervention measures.

##### Types of study designs for review of mechanisms, moderators, implementation and economic considerations (Objective 2)

To be included in the qualitative synthesis of the potential mechanisms, moderators, implementation factors, and economic considerations related to the intervention (Objective 2), each study needed to be (a) already included in the quantitative synthesis of impact evaluations (see above for review Objective 1); or (b) be an empirical study reporting on an eligible intervention. To be an empirical study, the authors must have either reported on primary quantitative or qualitative data or conducted secondary analysis of primary quantitative or qualitative data. We acknowledge that qualitative studies may not present “data” per se, but report on empirical work such as textual themes from key informant interviews or focus groups, or information gathered by observational methods (e.g., participant‐observers). Purely theoretical work, opinion pieces or research reports that only summarised, referenced or described previous intervention studies were not used for the qualitative synthesis.

#### 4.1.2 Types of participants

For both the review of effectiveness (Objective 1) and the review of mechanisms, moderators, implementation and economic considerations (Objective 2), this review included studies that use any of the following populations:

1. Individuals of any age, gender, or ethnicity; or
2. Micro places (e.g., street corners, buildings, police beats, street segments); or
3. Macro places (e.g., neighbourhoods, communities, police districts).

We placed no limits on the geographical region reported in the study. Specifically, we included studies conducted in high‐, low‐ and middle‐income countries.

### 4.2 Types of interventions

For both the review of effectiveness (Objective 1) and the review of mechanisms, moderators, implementation, and economic considerations (Objective 2), we included any police‐involved multiagency intervention that aimed to address terrorism, violent extremism or radicalisation to violence. Specifically, each study must have met two intervention criteria:

1. Report on a multiagency intervention where police are a partner, defined as some kind of a strategy, technique, approach, activity, campaign, training, programme, directive, or funding/organisational change that involved police and at least one other agency (Higginson, Eggins, et al., 2015). Police involvement was broadly defined as:

- Police initiation, development or leadership;
- Police are recipients of the intervention or the intervention is related, focused or targeted to police practices or
- Delivery or implementation of the intervention by police.

The other agencies or entities involved in the intervention could be government or nongovernmental agencies, including government agencies (e.g., education, immigration, customs, home affairs, employment, housing, health), local councils, businesses, communities (e.g., churches, mosques and other houses of worship), and services providers (e.g., resettlement agencies, local health providers).

**AND**

2. Report on a multiagency intervention with police as a partner that *aimed to address terrorism, violent extremism, or radicalisation to violence*, as defined or specified by study authors.

We anticipated that multiagency interventions with police as a partner that aim to address terrorism, violent extremism or radicalisation to violence may include:

- Police being trained OR police training or educating partner(s), to improve recognition, referral and responses to radicalisation, including guiding at‐risk populations towards numerous forms of support services offered by various partnerships, such as life skills mentoring, anger management sessions, and cognitive/behavioural therapy (Home Office, 2015a).
- Community awareness programs or training delivered to police OR police delivering community awareness training or programs to partner(s) to help partner(s) identify someone who may already be engaged in illegal terrorist‐related activity and are referred to the police (Home Office, 2009).
- Police working in partnership with universities to train, engage, intervene and consult on action plans to reduce at‐risk youth to extremist messaging (Angus, 2016).
- Approaches that involve police working with other agencies to refer, assess, or case‐manage individuals convicted of terrorism or identified as at‐risk of radicalisation (Cherney & Belton, 2019).
- Police partnering with other agencies to address radicalisation or extremism through regular structured/unstructured focus groups or meetings that may or may not be formalised (e.g., memoranda of understanding) or by forming task forces or multiagency intervention teams.
- Police working with external agencies to divert an individual away from violent extremism (e.g., UK Channel program, Home Office, 2015b).
- Police officers undertaking various forms of engagement with different community and agency stakeholders to help identify terrorist threats (Innes et al., 2011; Ramiriz et al., 2013).

#### 4.2.1 Types of outcome measures

##### Types of outcome measures for review of effectiveness (Objective 1)

For the review of effectiveness (Objective 1), we included studies with two main categories of outcomes. The first was radicalisation to violence. For the purposes of this review, radicalisation to violence was defined as the process by “which a person adopts extremist views and moves towards committing a violent act” (Hardy, 2018, p. 76; Jensen et al., 2018). It is important to note that “radicalisation” remains inconclusively defined in the literature (Heath‐Kelly, 2013) and violence is just one potential outcome of radicalisation (Angus, 2016; Hafez & Mullins, 2015; Schmid, 2013). We also recognise that terminology in the extant literature (e.g., radicalisation and extremism) is often used interchangeably (Borum, 2012), and that outcomes may not be labelled explicitly as “radicalisation to violence”. Other labels that may be used include: radicalisation (Horgan, 2009), extremism, violent extremism (Khalil & Zeuthen, 2016), political violence, ideologically motivated violence, political extremism (Lafree et al., 2018), violent radicalisation (Bartlet & Miller, 2012) and terrorism (Christmann, 2012).

We included outcome data measured through self‐report instruments, interviews, observations and/or official data (e.g., contact with police, calls‐for‐service reporting incidents, arrests, charges, prosecution, sentencing and correctional data). Some examples of how radicalisation to violence can be measured include:

- Violent Extremist Risk Assessment‐2 (VERA‐2): A risk assessment of the “likelihood of future violence by an identified offender who has been convicted of unlawful ideologically motivated violence” (RTI International, 2018, p. 10; Pressman & Flockton, 2012).
- Extremist Risk Guidance Factors (ERG 22+): Assesses the needs and risks of offenders who have either been convicted of an extremist offence or have shown behaviours or attitudes that raise concerns about their potential to commit extremist offences (Knudsen, 2020).
- IAT‐8: Assesses the effectiveness of a current intervention at reducing or altering the level of vulnerability to radicalisation (RTI International, 2018).
- RADAR assessments: Identifies “individuals who would benefit from services to help them disengage from violent extremism” (RTI International, 2018, p. 10) by assessing a variety of observations including religious understanding and knowledge, radicalisation source, intervention goals and progress undertaken to achieve these goals (Cherney & Belton, 2019)
- Terrorist Radicalisation Assessment Protocol (TRAP‐18): A professional judgement instrument for risk and threat assessment of individuals who may engage in lone‐actor terrorism (Meloy, 2018).

The second outcome category in the review was multiagency collaboration, broadly defined as a measure that relates to the quality and nature of the partnership between the agencies involved in the intervention. The quality and nature of collaborations or partnerships can be operationally defined in different ways, ranging from the degree of practical sharing of resources (Rosenbaum, 2002) to relational perspectives that encompass variables such as: frequency and quality of communication, shared goals and knowledge, and trust or respect (Bond & Gittell, 2010; Gittell, 2006). This review included both practical and relational measures of collaboration, captured by self‐report or official/administrative data, in one or more of the following categories:

- Information sharing (e.g., frequency, quality);
- Perceptions of trust, respect, or legitimacy within multiagency collaborations or
- Degree of shared goals and understanding between multiagency partners.

##### Types of outcome measures for review of mechanisms, moderators, implementation and economic considerations (Objective 2)

To be included in the qualitative synthesis of the potential mechanisms, moderators, implementation factors and economic considerations (Objective 2), no specific outcome measures were required. Any empirical study of a police‐involved multiagency programme that aimed to address terrorism, violent extremism, or radicalisation to violence was examined for empirical qualitative or quantitative data pertaining to mechanisms, moderators, implementation or economic considerations (see Supporting Information Appendix C for definitions). We note the differences in the conceptualisation of “outcomes” for quantitative and qualitative studies, whereby qualitative studies may not distinguish between different types of variables such as independent, predictor, outcome, moderator or mediator variables. Rather, qualitative studies are likely to present thematic textual data drawn from interviews, focus groups or observational methods. In addition, study authors may use mechanism, moderator, implementation and economic variables as outcome variables, or they may use data within these domains as mediators or moderators to explore their impact on study outcomes. To provide a comprehensive synthesis of potential mechanisms, moderators, implementation factors, and economic considerations, we included empirical studies that reported on data in any of these domains, regardless of whether the data are conceptualised as an “outcome variable”.

#### 4.2.2 Duration of follow‐up

For both the review of effectiveness (Objective 1) and the review of mechanisms, moderators, implementation, and economic considerations (Objective 2), we included studies with follow‐up periods of any length. If there was variation in the length of follow‐up across studies, we planned to group and synthesise studies with comparable follow‐up durations. For example, short (e.g., 0–3 months postintervention), medium (>3, <6 months) and long‐term follow‐up (>6 months postintervention). While this was not required for this review, we will take this approach in future updates to the review.

#### 4.2.3 Types of settings

We aimed to include studies reporting on an impact evaluation of an eligible intervention using eligible participants, outcome(s) and an eligible research design in any setting. Where there were multiple conceptually distinct settings, we planned to synthesise the studies within the settings separately. However, due to the paucity of information about study settings, we were unable to take this synthesis approach.

We assessed titles/abstracts and full‐text documents that were conducted or published between January 2002 and December 2018. Titles/abstracts published in a language other than English were translated using Google Scholar to identify if they were potentially eligible for the review. If eligibility could not be determined using Google Translate, the first author of the study was contacted to ascertain eligibility. If there was no response from the author or their contact details could not be located, the study was included in the “References to studies awaiting classification” section.

### 4.3 Search methods for identification of studies

The full search record for this review is provided in Supporting Information Appendix A. Electronic, grey literature, trial registry, and journal hand searches were conducted between November 2019 and March 2020. Reference harvesting, forward citation searching, and consultation with experts was conducted in November 2020. The overall search captured research conducted or published between January 2002 and December 2018. Due to the search functionalities of some websites, there was no ability to restrict searches to this date range, and so research from all publication years was assessed for eligibility.

#### 4.3.1 Electronic searches

The search for this review was led by the Global Policing Database (GPD) research team at the University of Queensland (Elizabeth Eggins, Lorelei Hine and Lorraine Mazerolle) and Queensland University of Technology (Angela Higginson). The University of Queensland is home to the GPD (www.gpd.uq.edu.au), which served as the main search location for this review. The GPD is a web‐based and searchable database designed to capture all published and unpublished experimental and quasi‐experimental evaluations of policing interventions conducted since 1950. There are no restrictions on the type of policing technique, type of outcome measure or language of the research (Higginson, Benier, et al., 2015). The GPD is compiled using systematic search and screening techniques, which are reported in Higginson, Eggins, et al. (2015) and summarised in Supporting Information Appendix B. Broadly, the GPD search protocol includes an extensive range of search locations to ensure that both published and unpublished research is captured across criminology and allied disciplines.

The GPD systematic search uses a broad range of policing and research search terms and systematically progresses the screening of the captured research in sequential stages with increasing specificity. At the initial title and abstract screening stage, records identified by the systematic search are screened on whether they are broadly about police or policing (see Higginson, Benier, et al., 2015). At subsequent full‐text screening stages, documents retained at the initial stage are then screened on whether they report on a quantitative impact evaluation of an intervention relating to police or policing, with no limits on outcome measures. As a result, refined corpuses of policing research can be searched and extracted from the GPD without the need to use policing search terms. Because our review captured both quantitative and qualitative studies of eligible interventions, we extracted data from the GPD from the point of title and abstract eligibility (i.e., is the document broadly about police or policing). We searched the title and abstracts within this corpus published between 2002 and 2018, using the following search terms: *terror* OR extrem* OR *radical*.

#### 4.3.2 Searching other resources

We also employed strategies to extend the GPD search. This included:

- Searching trial registries (those not indexed by WHO, but listed on the Office for Human Research Protections website https://www.hhs.gov/ohrp/international/clinical-trial-registries/index.html;
- Searching counterterrorism organisation websites (see Table 1);
- Conducting reference harvesting on existing reviews and eligible studies;
- Forward citation searching for all documents eligible for review Objective 1;
- Liaising with the Five Country Research and Development Network (5RD), and the DHS Advisory Board network for the Campbell Collaboration grants, to enquire about eligible studies that may not be publicly available;
- Personally contacting prominent scholars in the field and authors of eligible studies to enquire about eligible studies not yet disseminated or published; and
- Hand‐searching the following journals to identify eligible documents published in the 12 months prior to the systematic search date that may not have been indexed in academic databases:
  a. Critical Studies on Terrorism
  b. Dynamics of Asymmetric Conflict
  c. Intelligence and Counter Terrorism
  d. International Journal of Conflict and Violence
  e. Journal for Deradicalization
  f. Journal of Policing
  g. Perspectives on Terrorism
  h. Police Quarterly
  i. Policing—An international Journal of Police Strategies and Management,
  j. Policing & Society
  k. Sciences of Terrorism and Political Aggression
  l. Studies in Conflict & Terrorism
  m. Terrorism & Political Violence

**Table 1:** Grey literature search locations

Organisation	Website
Global Terrorism Research Centre (Monash University)	http://artsonline.monash.edu.au/gtrec/publications/
Triangle Centre on Terrorism and Homeland Security	https://sites.duke.edu/tcths/
Department of Homeland Security	https://www.dhs.gov/topics
Public Safety Canada	https://www.publicsafety.gc.ca/index-en.aspx
National Consortium for the Study of Terrorism and Responses to Terrorism (START)	https://www.start.umd.edu/
Terrorism Research Centre	http://www.terrorism.org/
Global Centre on Cooperative Security	https://www.globalcentre.org/publications/
Hedayah	http://www.hedayahcentre.org/publications
RAND Corporation	https://www.rand.org/topics/terrorism.html?content-type=research
Radicalization Awareness Network (RAN)	https://ec.europa.eu/home-affairs/what-we-do/networks/radicalization_awareness_network_en
RadicalizationResearch	https://www.radicalizationresearch.org/
Royal United Services Institute (RUSI)	https://rusi.org/
Impact Europe	http://impacteurope.eu/
National Criminal Justice Reference Service	https://www.ncjrs.gov/App/AbstractDB/AbstractDBSearch.aspx
Terrorism Research Centre (University of Arkansas)	https://terrorismresearch.uark.edu
International Association of Law Enforcement Intelligence Analysts	https://www.ialeia.org
Naval Post‐Graduate School	https://nps.edu

### 4.4 Data collection and analysis

#### 4.4.1 Selection of studies

##### Title and abstract screening

After removal of duplicates and ineligible documents types (e.g., book reviews, blog posts), all records captured by the systematic search were imported into review management software, *SysReview* (Higginson & Neville, 2014). Two review authors (E. E. and L. H.)—with assistance from trained research staff—screened the titles and abstracts for all records identified by the search according to the following exclusion criteria:

1. Ineligible document type (e.g., book review);
2. Record is not unique (i.e., duplicate);
3. Record is not about policing terrorism, radicalisation, or extremism.

Prior to independent screening, all staff engaged in title and abstract screening assessed the same set of 50 records and two review authors (E. E. and L. H.) compared their judgements to verify consistent decision‐making and provide feedback to each screener. In addition, a sample of 10% of all excluded titles and abstracts across all screeners were cross‐checked for accuracy by one review author (L. H.) and any disagreements were mediated by a different review author (E. E.).

Although all efforts were made to remove ineligible document types and duplicates prior to screening, automated and manual cleaning can be less than perfect. As such, the first two exclusion criteria were used to remove ineligible document types and duplicates prior to screening each record on substantive content relevance. It is important to note that “policing” is broadly operationalised in both the GPD screening and the screening for this review. Specifically, a title and abstract can be screened as being about policing if, for example: police are study participants, police are involved in implementing an intervention (alone or in partnership with others), or the focus of the research appears to be police tools, technologies or techniques (see Higginson, Benier, et al., 2015).

All potentially eligible records then progressed to full‐text eligibility screening. Most records indexed in the GPD have a pre‐existing full‐text document. However, records from the additional searches that were deemed as potentially eligible at the title and abstract screening stage progressed to literature retrieval, where attempts were made to locate the full‐text document. Where full‐text documents could not be retrieved via existing university resources, they were ordered through the review authors' university libraries. If the full‐text document could not be located, the abstract was used to assess whether the study met full‐text eligibility criteria. Where a decision could not be unequivocally made about eligibility based on the abstract, the record was categorised as a study awaiting classification (see “References to studies awaiting classification” section).

##### Full‐text eligibility screening

Two review authors (E. E. and L. H.)—with assistance from trained research staff—screened the full‐text of each document for final eligibility using a two‐stage process. The following exclusion criteria was used for the first stage of screening:

1. Ineligible document type (e.g., book review);
2. Document is not unique (i.e., duplicate);
3. Document does not refer to an eligible intervention;
4. Document does not report on an empirical study of a multiagency intervention with police as a partner that aims to address radicalisation, terrorism, or extremism.

While all efforts were made to remove ineligible document types and duplicate documents in earlier stages, these types of records can occasionally progress into later stages of screening (e.g., where duplicate records are not adjacent to each other during screening or where screeners cannot unequivocally determine the document type based on the title and abstract). Therefore, the first two exclusion criteria were used to remove ineligible document types and duplicates before they progressed to the more time‐intensive full‐text screening on inclusion criteria.

The purpose of the second stage of screening was to categorise studies according to the review objectives. Specifically, screeners were asked to determine whether each study was (a) a quantitative impact evaluation of an eligible intervention, using an eligible research design, outcomes, and participants; (b) an empirical (qualitative and/or quantitative) study describing the implementation factors, economic considerations, moderators, and/or mechanisms of an eligible intervention; or (c) a study that eligible for both (a) and (b).

Two review authors (E. E. and L. H.) trained research staff to screen the documents using a standardised screening companion. Prior to independent screening, each review author or research staff member conducting full‐text document screening was required to screen the same set of 25 documents and their answers were compared against the answers determined by two review authors (E. E. and L. H.). Feedback was provided to all screeners prior to beginning independent screening. A random 5% sample of each screener's exclusion screenings were cross‐checked to identify false negative screening decisions. If a screener's decisions were deemed unreliable due to a high rate of false negatives (≥5%), the protocol stated that their exclusion screenings would be reassigned to another screener (Mazerolle, Cherney et al., 2020). There were no instances of high false negative screening decisions. Any disagreements in determining a study's final eligibility for the review were resolved via discussion with a third review author (A. H.).

#### 4.4.2 Data extraction and management

Eligible documents were coded using the coding companion provided in Supporting Information Appendix C. The level of coding was dependent on the category each study was assigned. Data pertaining to the general study characteristics (e.g., document type, study location) were extracted for all studies.

For studies eligible for the review of effectiveness (Objective 1), data was extracted according to the following general domains:

1. Participants (e.g., sample characteristics by condition, attrition)
2. Intervention (e.g., intervention components, intensity, setting)
3. Outcomes (e.g., conceptualisation, mode of measurement, time‐points)
4. Research methodology (e.g., design, unit and type of assignment)
5. Effect size data
6. Risk of bias

For studies eligible for the review of mechanisms, moderators, implementation and economic considerations (Objective 2), each study was first rated on the quality of the evidence across the mechanism, moderator, implementation and economic domains. We used the EMMIE (Effectiveness, Mechanisms, Moderators, Implementation, Economics) appraisal tool developed by Johnson et al. (2015) to guide our decisions (see Table 2). In addition to the criteria delineated by Johnson et al. (2015), to reach a rating of 3 or 4 on the mechanism and moderator domains, studies needed to explicate an independent intervention variable that fit the eligibility criteria for this review, measure an explicit moderator or mechanism, and measure and report on a separate dependent variable.

**Table 2:** EMMIE appraisal tool

Domain	Rating
Mechanism	0 = No reference to theory, simple black box 1 = General statement of assumed theory 2 = Detailed description of theory, drawn from prior work 3 = Full description of the theory of change and testable predictions generated from it 4 = Full description of the theory of change and robust analysis of whether it is operating as expected
Moderator	0 = No reference to relevant contextual conditions that may be necessary 1 = Ad hoc description of possible relevant contextual conditions 2 = Test the effects of contextual conditions defined post hoc using available variables 3 = Theoretically grounded description of relevant contextual conditions 4 = Collection and analysis of relevant data relating to theoretically grounded moderators and contexts
Implementation	0 = No account of implementation or implementation challenges 1 = Ad hoc comments on implementation or implementation challenges 2 = Concerted efforts to document implementation or implementation challenges 3 = Evidence‐based account of levels of implementation or implementation challenges 4 = Complete evidence‐based account of implementation or implementation challenges and specification of what would be necessary for replication elsewhere
Economics	0 = No mention of costs and/or benefits 1 = Only direct or explicit costs and/or benefits estimated 2 = Direct or explicit and indirect costs and/or benefits estimated 3 = Marginal or total or opportunity costs and/or benefits estimated 4 = Marginal or total or opportunity costs and/or benefits estimated by bearer (or recipient) estimated

For studies that reached a rating of 3 or more on any of the mechanism, moderator, implementation and economic domains, data were extracted according to the following (see also “Treatment of qualitative research” section):

1. Research approach (e.g., design, sampling)
2. Participant characteristics
3. Mechanisms that may explain intervention outcomes
4. Moderators that may impact intervention outcomes
5. Implementation considerations (e.g., barriers or facilitators)
6. Economic considerations

For studies that did not meet a rating of 3 on any domain using the EMMIE tool, we coded each study according to document type, setting, intervention, participants, research approach and rating of the level of evidence for mechanisms, moderators, implementation and economics domain.

We anticipated that some studies included in the effectiveness component of the review (Objective 1) may report information eligible for the mechanism, moderator, implementation and economic component of the review (Objective 2), yet this information may not be collected, analysed or reported in the same way as the effectiveness data. Therefore, if studies were eligible for both components data were extracted according to both of the abovementioned frameworks.

All studies eligible for the review of effectiveness (Objective 1) were independently double coded. For studies eligible for the Objective 2 with a rating of three or more on the EMMIE tool, at least one study from each EMMIE domain (Mechanisms, Moderators, Implementation, Economic) and at least one study per coder were independently double coded. The results of this double coding (30% of 26 studies, *n* = 8) was assessed by one review author (E. E.) prior to independent coding for Objective 2, with feedback provided to coders to ensure consistency. The remaining 18 studies reaching a rating of 3 on the EMMIE tool were independently coded, with the extracted information verified upon synthesis by at least one review author (A. C., E. E., L. H.). The 155 studies included in the qualitative synthesis that did not reach a rating of at least three on the EMMIE tool were independently coded by two study authors (E. E. and L. H.).

#### 4.4.3 Assessment of risk of bias in included studies

Due to the nature of the included studies, we selected risk of bias tools most appropriate to the type of research under consideration. In addition, risk of bias assessments were only conducted for the studies included in the effectiveness component (Objective 1, *n* = 5) and studies included in the Objective 2 that reached a rating of at least one rating of 3 on the EMMIE appraisal tool (*n* = 26).

Only one study included in the effectiveness component of the review (Objective 1) was a prospective intervention suited to the Cochrane nonrandomised risk of bias tool (ROBINS‐I). This tool guides rating across seven domains to determine low, moderate, serious, or critical risk of bias, or no information to make a judgement (Sterne et al., 2016). The *confounding* domain assesses whether the study accounts for the baseline and/or time‐varying prognostic factors (e.g., socioeconomic status). The *selection* domain refers to biases internal to the study in terms of the exclusion of some participants, outcome events, for follow‐up of some participants that is related to both intervention and outcome. The *classification of interventions* domain refers to differential (i.e., related to the outcome) or nondifferential (i.e., unrelated to the outcome) misclassification of the intervention status of participants. The *measurement of outcomes* domain assesses whether bias was introduced from differential (i.e., related to intervention status) or nondifferential (i.e., unrelated to intervention status) errors in the measurement of outcome data (e.g., if outcome measures were assessed using different methods for different groups). The *deviations from intended interventions* refers to differences arising in intended and actual intervention practices that took place within the study. The *missing data* domain measures bias due to the level and nature of missing information (e.g., from attrition, or data missing from baseline or outcome measurements). Finally, the *selection of reported results* domain is concerned with reporting results in a way that depends on the findings (e.g., omitting findings based on statistical significance or direction of effect). The results of the risk of bias assessment are provided in a written summary and table. If future updates of the review identify additional eligible studies suited to the ROBINS‐I tool, the results of the risk of bias assessment will also be depicted in a risk of bias summary figure.

The remaining four studies included under Objective 1 were cross‐sectional surveys where one of the independent variables measured an eligible intervention in a way to allow for a counterfactual analysis. These studies were not suited to the ROBINS‐I tool. Consequently, the Effective Public Health Project (EPHPP) tool was used to assess risk of bias. This tool guides the appraisal of studies across six domains: selection bias, study design, confounders, blinding, data collection methods, and withdrawals and drop‐outs. The withdrawals/drop‐outs domain was omitted because the developers state that these questions are not applicable for one‐time survey studies (response rate is captured under questions for the selection bias domain). Based on the guidance specified by the developers, studies are rated as either “strong”, “moderate” or “weak” for each domain. Overall, studies are rated as “strong” (low risk of bias) if they receive no “weak” ratings on any domains, “moderate” if they have only one “weak” rating across domains, or “weak” (high risk of bias) if they receive two or more “weak” ratings across domains.

Five the 26 studies with a rating of 3 or more on the EMMIE appraisal tool that were included in the implementation, mechanisms, moderators, and economics component of the review (Objective 2) were also included in the effectiveness component of the review (Objective 1). As such, the risk of bias for these studies was assessed as outlined above. The risk of bias for the remaining 21 studies was assessed using the suite of CASP critical assessment checklists (https://casp-uk.net/casp-tools-checklists/). This suite includes a checklist for case‐control studies, cohort studies, economic studies, and qualitative studies, all of which contain questions to guiding the rating for each domain (see Supporting Information Appendix D). The selection of the appropriate checklist was based on the nature of the study under consideration and is delineated in the results section.

#### 4.4.4 Measures of treatment effect

Of the five studies included in the review of effectiveness (Objective 1), only one contained sufficient data to calculate effect sizes and used an outcome falling under the category of radicalisation to violence (Williams et al., 2016). This study used a continuous measure of outcome data collected from individual participants. The independent variable was dichotomous, as the participants were either in the intervention group or the comparison group. The specific data required to calculate effect sizes was not provided in the eligible study reports but was provided by the study authors via personal communication. *RevMan* was used to calculate standardised mean differences (SMD) and their 95% confidence intervals.

The remaining four studies reported on continuous outcomes that were eligible as a multiagency collaboration outcome category and reported coefficients from statistical tests to represent the intervention effect. It is important to note that the variables that are conceptualised in this review as intervention variables were not the focus of the study, rather, they were one of several independent variables included in the study's models. Carter et al. (2014) reported incident rate ratio (IRR) coefficients from a negative binomial regression model that used an ordinal intervention variable measuring the agency's alignment with DHS TCL. Baldwin (2010) reported the unstandardised coefficients from a linear regression model that used a continuous intervention variable measuring the number of multiagency homeland security partners. Burruss et al. (2012) reported standardised coefficients from structural equation models that used an ordinal intervention variable measuring participants' perception of the influence of partner grants on current homeland security practice. Finally, Stewart and Oliver (2014) reported unstandardised regression coefficients from zero‐inflated negative binomial regression models that used a dichotomous intervention variable measuring whether or not the agency had received homeland security grants.

Where it was possible to calculate a standardised effect size for regression coefficients, we calculated *r*. The effect size *r* is interpreted as the number of standard deviation changes in the outcome for every one standard deviation change in the intervention variable, controlling for other predictor variables.

Unstandardised regression coefficients (*B*) from linear regression models (Baldwin, 2010) and structural equation models (Burruss et al., 2012) were converted to *r* using the following formulae, and 95% confidence intervals were calculated from *r* and *SE*_*r*_:

$$r=({\mathrm{SD}}_{x}\times B)/{\mathrm{SD}}_{y}.$$

$${\mathrm{SE}}_{r}=(r\times {\mathrm{SE}}_{B})/B.$$

$$95\%\;{\text{LCL}}_{r}=r–1.96\times {\mathrm{SE}}_{r}$$

$$95\%\;{\text{UCL}}_{r}=r+1.96\times {\mathrm{SE}}_{r}.$$

Burruss et al. (2012) reported both the standardised and unstandardised regression coefficients, but did not report *SE_β_, SD_x_* or *SD_y_* to allow calculation of *r* and the 95% confidence intervals for *r*. We calculated SD_y_ from the reported data using:

$${\mathrm{SD}}_{y}=B\times ({\mathrm{SD}}_{x}/\beta ).$$

The authors were contacted for additional data, and provided *SD_x_* by personal communication.

As there is currently no appropriate method to standardise the coefficients from negative binomial models (Wilson, 2020, personal correspondence), for studies that use negative binomial regression (Carter et al., 2014) or zero inflated negative binomial regression (Stewart & Oliver, 2014) we do not report a standardised effect size for the models presented in these studies. Rather, we describe the results using the metric reported by study authors. Carter et al. (2014) reported their results as exponentiated coefficients or IRRs, and Stewart and Oliver (2014) reported unstandardised coefficients (*B*). We calculated 95% confidence intervals from these data.

For future updates of this review, we aim to follow the procedures outlined in the protocol to extract and/or calculate measures of treatment effect wherever possible (Mazerolle, Cherney et al., 2020).

#### 4.4.5 Unit of analysis issues

Unit of analysis and/or dependency issues may occur when (a) multiple documents report on a single empirical study; (b) multiple conceptually similar outcomes are reported in the one document; (c) data is reported for multiple time‐points and/or (d) studies have clustering in their research design. None of these issues were relevant when quantifying and synthesising treatment effects for this review. For future updates of this review, our approach for handling these issues is specified in the protocol (Mazerolle, Cherney et al., 2020).

#### 4.4.6 Dealing with missing data

Due to the number of studies included in this review, study authors were only contacted by email to seek missing data if the data would (a) allow for quantitatively synthesising studies via meta‐analysis or reporting effect sizes; or (b) would have changed the risk of bias rating for the study. The results section also specifies which data were obtained from published reports of a study and which data were obtained directly from study authors (not available in the public domain). For future updates of this review, we will follow this procedure.

#### 4.4.7 Assessment of heterogeneity

Due to the inability to conduct meta‐analyses using the studies included in the effectiveness component of this review (Objective 1), we were unable to statistically assess heterogeneity. However, we provide narrative text which explores the differences between the the included studies. For updates of this review, we will implement either this approach or the statistical assessment approach specified in the review protocol (Mazerolle, Cherney et al., 2020).

#### 4.4.8 Assessment of reporting biases

Due to the inability to conduct meta‐analyses using the studies included in the effectiveness component of this review (Objective 1), we were unable to statistically assess reporting/publication biases. For updates of this review, we will implement the approach specified in the review protocol (i.e., inspecting funnel plots for asymmetry, conducting subgroup analyses to assess if the effect sizes from the published and unpublished documents are significantly different).

#### 4.4.9 Data synthesis

##### Treatment of quantitative evaluation research (Objective 1)

Due to the nature of the studies included in this component of the review, we were unable to conduct meta‐analyses to synthesise the studies. Only one eligible study assessed the impact of the intervention on radicalisation outcomes, and of the four eligible studies using multiagency outcome measures, the disparate intervention and outcomes precluded meta‐analysis. Rather, we describe each study and estimates of treatment effects either using single standardised effect sizes with their corresponding confidence intervals (Williams et al., 2016) or the coefficient reported by study authors where a standardised effect size could not be calculated (Carter et al., 2014; Stewart & Oliver, 2014). For updates of this review, we will use either this approach or the data synthesis approach outlined in the protocol (Mazerolle, Cherney et al., 2020).

##### Treatment of qualitative research (Objective 2)

For the review of mechanisms, moderators, implementation, and economic considerations (Objective 2) we drew on the EMMIE framework developed by the UK's *What Works for Crime Reduction Centre* (Johnson et al., 2015; Thornton et al., 2019). This framework aims to structure the extraction and discussion of the **E**ffects of an intervention, the **M**echanisms by which the intervention is believed to work, the **M**oderators that may vary intervention effectiveness (e.g., characteristics of target people or places), **I**mplementation considerations (e.g., required resources, training), and **E**conomic implications for the intervention in terms of costs and benefits (Johnson et al., 2015; Thornton et al., 2019). Objective 1 of this review encompasses the Effectiveness part of the EMMIE framework, so Objective 2 focuses on qualitatively synthesising the mechanisms, moderators, implementation and economic domains. The data extraction for these domains (Supporting Information Appendix C) was adapted from the EMMIE codebook (Tompson et al., 2015), and has been utilised in a number of realist‐informed systematic reviews (e.g., Belur et al., 2017; Sidebottom et al., 2015; see also Gielen, 2015) and for rating the evidence of systematic reviews in the area of criminal justice (see https://whatworks.college.police.uk/toolkit/Pages/Welcome.aspx).

There are multiple approaches available for qualitative synthesis, yet the development of a clear set of guidelines has been a complex and long‐term problem (Booth et al., 2018; Noyes et al., 2019) and many of the methods have not been thoroughly evaluated for use in mixed‐methods systematic reviews (Dixon‐Woods et al., 2005, 2006; Popay et al., 2006; Pope et al., 2007). Explicitly labelling our qualitative synthesis approach is also complicated by the variations in the terminology in the literature and significant overlap in techniques within different synthesis approaches (Booth et al., 2016; Pope et al., 2007).

We used a Framework Synthesis method to synthesise the qualitative data (see Booth et al., 2016), which is an overarching approach that encompasses analogous methods such as content analysis, framework analysis, and aggregate synthesis (see Booth et al., 2016; Booth & Carroll, 2015; Dixon‐Woods et al., 2005, 2006; Dixon‐Woods, 2011; Noyes et al., 2019; Popay et al., 2006). Broadly, these methods use systematic rules or a framework to arrange data into distinct categories that are then synthesised using a variety of techniques such as tables, matrices, and narrative textual summaries (e.g., see Belur et al., 2017; Petrosino et al., 2012; Sidebottom et al., 2015). Using the data extracted from each study eligible for the qualitative component of the review, we categorise and then synthesise the studies in text and tabular format. We then provide specific subsections aligning to the EMMIE domains of mechanisms, moderators, implementation, and economics. Within each domain subsection, narrative text and tables summarise the number of studies reporting data for that domain, the types research approaches used by included studies (e.g., design and participants), the specific findings for mechanism, moderator, and economic domains, and overarching themes for the implementation barriers and facilitators.

#### 4.4.10 Subgroup analysis and investigation of heterogeneity

We had planned to use subgroup analyses to assess whether the impact of the intervention varied by the following factors: geographical location, target population, nature of the intervention approach (e.g., number of components, specific intervention techniques), and number and type of multiagency partners. However, due to the inability to conduct meta‐analyses using the studies included in the effectiveness component of the review (Objective 1), we were unable to conduct subgroup analyses. For updates of this review, provided sufficient data is found, we will follow the subgroup analysis approach specified in the review protocol (Mazerolle, Cherney et al., 2020).

#### 4.4.11 Sensitivity analysis

We had planned to use sensitivity analyses to assess the impact of risk of bias on estimates of the treatment effect. However, due to the inability to conduct meta‐analyses using the studies included in the effectiveness component of the review (Objective 1), we were unable to conduct these analyses. For updates of this review, provided sufficient data is found, we will follow the sensitivity analysis approach specified in the review protocol (Mazerolle, Cherney et al., 2020).

### 4.5 Deviations from the protocol

Our review made six deviations from the protocol. First, all studies eligible for the review of effectiveness (Objective 1), and 30% (*n* = 8) of the studies eligible for the review of mechanisms, moderators, implementation factors and economic considerations (Objective 2) were independently double coded. This equated to at least one double coding for each coder and at least one double coding for each of the EMMIE domains. The remaining 18 studies included under Objective 2 were independently coded, but verified upon synthesis by at least one review author (A. C., E. E., L. H.). This is a deviation from the protocol which stated we would utilise a set of five training documents to determine coding accuracy prior to independent coding of all studies included in the review (Mazerolle, Cherney et al., 2020). The reason for this deviation is because we deemed it more important to ensure that coding was consistent across coders by domain for the EMMIE synthesis and that all coding was consistent for the effectiveness studies.

Second, due to the large number of studies included in Objective 2 and the wide variation in quality or explicit focus on the mechanisms, moderators, implementation and economic components within the documents, we did not harvest the references lists or conduct forward citation searching for these studies.

Third, we used the EMMIE appraisal tool to grade each study eligible for review Objective 2 across the mechanism, moderator, implementation, and economic domains. Studies that met a minimum threshold of 3 on each domain were then coded using the form in Supporting Information Appendix C and synthesised narratively in the results section. Any study that did not meet a threshold of 3 was not assessed for risk of bias and was only lightly coded for basic information about the setting, intervention, participants and EMMIE domains. The rationale for this was that the EMMIE appraisal tool provided a preliminary indicator of the quality and depth of evidence in the study. We considered this approach to be analogous to the protocol for the review (Mazerolle, Cherney et al., 2020) whereby studies would not be included in the synthesis if the answer to the following items on the CASP qualitative appraisal tool were “No” or “Can't tell”: (a) Is the research design appropriate to answer the question?; and (b) Was the sampling strategy appropriate to the aims of the research? (see Higginson, Benier, et al., 2015). However, studies rated <3 on the EMMIE appraisal tool were included in an overall summary table, with broad themes noted in the results section. For a full description of this approach, please refer to the “Data extraction and management approach” section.

Our fourth deviation was not contacting study authors where there was either missing information or “unclear” ratings during the risk of bias assessment. We chose this approach because the additional information would not have changed the overall risk of bias result. However, for updates of the review, we aim to follow the original protocol (Mazerolle, Cherney et al., 2020).

Fifth, we used a more suitable risk of bias assessment tool to appraise four of the five studies included in the effectiveness component of the review (Objective 1), rather than the ROBINS‐I tool. The rationale for this deviation is provided in Section 4.4.3.

Sixth, we had planned to examine whether the effectiveness of eligible interventions varied by the following factors: geographical location, target population, nature of the intervention approach (e.g., number of components, specific intervention techniques), and number and type of multiagency partners. Due to the limited evidence located, we did not conduct this analysis.

## 5 RESULTS

### 5.1 Description of studies

#### 5.1.1 Results of the search

**Figure 1:**
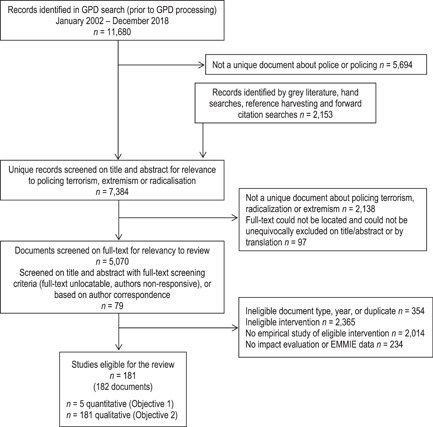
PRISMA flowchart

The results of the search and subsequent screening are summarised in Figure 1. The overall GPD systematic search identified 11,680 references published between January 2002 and December 2018 prior to any systematic processing that underpins the GPD (see Supporting Information Appendices B and C). Of these, 5986 references were eligible after title and abstract screening as being potentially about police or policing, and were imported into *SysReview* to be assessed for eligibility for this review (Higginson & Neville, 2014). The GPD search results were combined with the records identified by the grey literature search, hand searches, reference harvesting, forward citation searching, and consultation with experts (*n* = 2153) to generate a corpus of 7384 records (after preliminary duplicate removal).

A total of 5246 records were eligible after title and abstract screening as being potentially about policing terrorism, radicalisation or extremism. We obtained the full‐text documents for 5070 of these records via institutional libraries or correspondence with document authors, including 145 documents that were written in a language other than English. For documents written in a language other than English, we first assessed the title and abstract of the article using the more specific full‐text screening criteria, as almost all abstracts were written in English. For the remaining documents, we used Google Translate to translate the documents and assess their eligibility. These strategies resulted in the exclusion of 112 documents. For those where translation was not possible or the translation did not permit an unequivocal eligibility decision, we attempted to contact study authors for clarification (*n* = 33). Three study authors responded and confirmed their study was ineligible for the review. The remaining 30 studies are listed in the “Studies awaiting classification” reference list.

The full‐text documents for 176 records screened as potentially about policing terrorism, extremism or radicalisation could not be located via institutional libraries (including *n* = 12 written in a language other than English). Of these, just over one third were conference presentations or magazine articles, with the rest of evenly distributed amongst the following categories of document types: journal articles, books or book chapters, reports or working papers (government and technical), and theses. We handled the processing of records with no full‐text document in two stages. First, we screened their titles and abstracts using the more specific full‐text screening criteria. Second, we attempted to contact the authors where we could not unequivocally exclude the record by screening the title and abstract using the full‐text screening criteria. These two strategies resulted in the exclusion of 79 documents. For the remaining 97 records: (a) authors could not provide the document or recall whether it met inclusion criteria; (b) no response was received from document authors or (c) current contact details for document authors could not be found. These documents are reported in the “References to studies awaiting classification” list.

Of the 5149 studies screened on the full‐text screening criteria, five studies met the review eligibility criteria for the review of effectiveness (Objective 1) and 181 studies (reported in 182 documents) met the inclusion criteria for the mechanisms, moderators, implementation and economic component of the review (Objective 2). The five studies eligible for the quantitative analysis were also eligible for Objective 2.

#### 5.1.2 Included studies

##### Review of quantitative effectiveness studies (Objective 1)

###### Radicalisation to violence outcome category

One study examined the impact of a police‐involved multiagency intervention to counter radicalisation to violence using a radicalisation to violence outcome. Williams et al. (2016) evaluated the impact of the World Organisation for Resource Development Education (WORDE) programme on measures aligned with radicalisation to violence. The intervention was implemented in Montgomery County, Maryland, United States in 2015 and was funded by the National Institute of Justice. The intervention model was led by a nonprofit group of Muslim scholars and community leaders who collaborated with experts in policy analysis, theology, academia, and development. The programme was comprised of three “interlocking” components:

1. Community education via (a) town hall meetings that aimed to provide opportunities for dialogue between public officials and the public, and (b) educational topics including youth engagement, conflict resolution, and family support.
2. Capacity‐building for agencies including law enforcement, community organisations and social services (e.g., social workers and psychologists) to create a referral network. This network was to identify and assist individuals who may be at‐risk of becoming radicalised or committing violent offences.
3. Community participation in organised volunteerism and/or multicultural activities (e.g., art projects, work to assist homeless people). This component did not explicitly involve law enforcement.

Overall, the programme aimed to foster and/or maintain cooperative relationships and networks between law enforcement and social services and community members. The community members targeted by the WORDE programme were youth and adults who were sensitised to issues of violent extremism, civically engaged, and residing in Montgomery County in Maryland, although the composition of the sample in relation to these categories is not explicitly reported in the study.

To generate the treatment group for this study (*n* = 133), the researchers used a prescreening questionnaire to generate a stratified random sample of community members involved with the WORDE programme, who were then invited to participate in the survey. For the comparison group, the researchers targeted a sample which was considered to be engaged with multicultural or volunteerism events and activities, but not with the WORDE programme. This comparison group (*n* = 58) were recruited through school list‐serves, electronic bulletin boards such as Google Groups, and interfaith partners that were not involved in the implementation of WORDE. These groups were predominantly aged in their mid‐twenties (treatment $\overset{®}{X}$ = 26.54 years, comparison $\overset{®}{X}$ = 27.33), male (treatment = 75.9%; comparison = 51.7%), and Caucasian (treatment = 91.7%, comparison = 57.9%).^2^ While the authors report that the groups were propensity score matched using age, religion, race and educational level, it is unclear whether this was implemented as intended.

The impact of the WORDE intervention was assessed on knowledge of out‐group cultures, attitudes (e.g., towards different religions), and behavioural outcomes (e.g., coping skills). Williams et al. (2016) used self‐report surveys to capture the outcomes at one time point (the period of engagement with the programme is unknown) using a 14‐item measure constructed for the study, the *Brief Volunteer Program Outcome Assessment*. This measure used a 7‐point Likert scale from “completely disagree” to “completely agree” whereby higher values equated to greater agreement with each statement. The items were:

“Thinking of when you volunteer, please rate your level of agreement with the following statements:

- I feel welcome*
- I feel a part of something bigger than myself*
- I feel a sense of teamwork*
- I make friendships that are active beyond the event*
- I make friends with people from other races*
- I feel useful
- I have responsibilities
- I have leadership responsibilities
- I feel a sense of purpose
- I feel free of peer pressure*
- I feel accepted*
- I wouldn't feel lonely
- I wouldn't feel afraid to talk to others*
- I learn about cultures other than my own*” (p. 157).

Of the above items, nine were deemed as eligible outcomes for this review (marked with asterisk) and were considered to fall under the general banner of an outcome linked with existing measures that aim to assess the level of vulnerability to radicalisation (e.g., IAT‐8, RADAR). For example, Barrelle (2015) identifies factors associated with the deradicalisation process such as the level of acceptance and/or engagement with cultural and religious differences or pluralistic views and modification of group or personal identity. It is important to note that it is not explicitly clear whether treatment participants were all directly exposed to the two intervention components that involved police. Hence, these evaluation outcomes may not be direct measures of how effective police‐involved multiagency interventions are for countering violent radicalisation. In addition, neither the treatment group nor the comparison group were noted by the study authors as being at risk for radicalisation.

###### Multiagency collaboration outcome category

Four studies provided data to measure the impact of a police‐involved multiagency intervention to counter radicalisation to violence using a multiagency collaboration outcome (Baldwin, 2010; Burruss et al., 2012; Carter et al., 2014; Stewart & Oliver, 2014). Each of the studies used quasi‐experimental designs and reported regression or structural equation models with multiple predictor variables. The eligible interventions for this review were selected from these independent variables, and included: the alignment of local practice with national counterterrorism guidelines that emphasise multiagency working; the number of counterterrorism partnerships; and collaboration via grants to facilitate strategies for countering radicalisation to violence. All of the interventions involved governmental agencies as partners or did not specify the exact type and/or number of partners. The data for all four studies were self‐report using retrospective cross‐sectional surveys of practitioners (mainly police).

Carter et al. (2014) surveyed 272 U.S. state, local and tribal law enforcement agencies from a population of personnel (*N* = 967) who attended a national training programme funded by the U.S. DHS. The sample, deemed to be knowledgeable about their agencies' intelligence functions, completed a self‐report survey which included a measure of the degree to which their agency aligned with the DHS TCL. This independent variable was conceptualised as the multiagency intervention for our review, as the TCL explicitly encourages multiagency working in the context of terrorism, extremism and/or radicalisation, by providing agencies with a guide through which to extend their ability to prevent, respond to, and recover from major events including terrorism. The study operationalised this variable as the degree to which the respondent felt their agency's intelligence function aligned with the TCL, using a four‐point Likert scale from 1 (not at all) to 4 (completely). The study used a series of quasi‐experimental negative binomial regression models to evaluate the impact of reporting more or less alignment with the TCL on three eligible multiagency outcome measures, controlling for the effect of multiple key policing variables. The first outcome was a “relationships scale” measuring the extent to which the agency had close working relationships with external organisations (i.e., U.S. FBI, other federal law enforcement agencies, state law enforcement agencies, local law enforcement agencies, the respondent's state fusion centre, and other state fusion centres).^3^ The second outcome was a “provide intelligence scale” which measured the frequency with which the agency reported providing actionable intelligence to any of the aforementioned agencies. Finally, the third outcome measure is a “receive intelligence scale” measuring the frequency with which the agency reported receiving actionable intelligence from the external agencies.^4^ In all three scales higher scores equated to closer or more frequent multiagency working with more agencies.

Baldwin (2010) conducted a survey of practitioners who were “law enforcement supporters or professionals” (p. 28) and members of the Tennessee Chiefs of Police Association. A total of 2457 members were identified, however, only 1938 had valid email addresses and were invited to participate in the survey. A total of 294 practitioners completed the survey (15.17% of members invited) and the sample of was comprised of 18.4% females and 85.5% white ethnicity. The age of participants fell into four categories: (1) 21–30 years (2.1%); (2) 31–40 years (27.1%); (3) 41–50 years (40.5%) and (4) 51 years and over (30.2%). In regards to distribution across organisational settings and roles, 53.3% of agencies were municipal, 50% had 100 or more staff, 51% were in urban jurisdictions, 44% of respondents had served 16 or more years in their agency, 53.6% had served 16 or more years in their profession, and 49.7% had a college degree. The independent variable conceptualised as the intervention for this review was a continuous survey item that asked participants to indicate from a list which categories of agencies or organisations that they had collaborated with “on homeland security issues” (p. 35).^5^ The organisations were: other local governments, local military installations, state government agencies, the FBI, Department of Justice, DHS, Department of Defence, and nongovernment agencies. The number of collaboration types ranged from 1 to 7 ($\overset{®}{X}$ = 3.6, SD = 1.8). This variable was conceptualised for the purposes of this review as a measure of the degree of multiagency collaboration. The study used a series of quasi‐experimental linear regression models to assess the influence of differing levels of collaborations on three eligible multiagency outcome measures, controlling for the effect of multiple key policing variables. The three eligible outcomes measured participants' agreement with “statements regarding the clarity of the mission, responsibilities, goals and strategies of homeland security” at the federal, state, and local levels (Baldwin, 2010, p. 38). For the purposes of this review, these outcomes were conceptualised as measures of understanding between multiagency partners. At the federal level, respondents were asked to rate their level of agreement with the following statement: “the mission, responsibilities, strategies and/or goals of the DHS are clearly understood or defined” (p. 62). At the state level, respondents were asked to rate their level of agreement with the following statement: “the mission and responsibilities of state and local homeland security strategies and/or goals are clearly defined or understood” (p. 63). At the department level, respondents were asked to rate their level of agreement with the following statement: “the mission, responsibilities, strategies, and/or goals of your department's homeland security vision is clearly understood or defined” (p. 63). All three outcome items were measured on a 5‐point Likert scale, with higher scores indicative of higher levels of agreement with the survey item.

Burruss et al. (2012) used data from a self‐report survey that used a stratified national sample to examine the level of national security preparedness among a sample of 350 small (≤25 sworn personnel) state and local US law enforcement agencies. While these authors used a range of variables to examine multiagency working (refer to the review of mechanisms, moderators, implementation, and economic considerations (Objective 2) section for a description of these), only one variable, “grants,” was operationalised as an eligible intervention variable for the purposes of this review. Burruss et al. (2012) measured the respondents' perceptions of the level of influence that grants from four partners (DHS, private industry, community, or corporate bodies) had on their agency's current practices regarding homeland security prevention, preparedness, response and recovery. Grants from partners was conceptualised as a measure of multiagency working, as it asked participants to report, on a three‐point Likert scale from 0 (not at all influential) to 2 (very influential), their perceptions of the level of influence of (1) federal or state equipment grants, (2) training grants, (3) personal grants, and (4) private or corporate grants. The authors constructed an additive scale based on these four measures (Cronbach's *α* = .860, $\overset{®}{X}$ = 3.253, *SD* = 2.596, min = 0, max = 8).^6^ The study used a series of quasi‐experimental structural equation models to evaluate the direct impact of the influence of grants on an eligible multiagency outcome measure, controlling for the effect of multiple key policing and terrorism variables. The eligible outcome was homeland security preparedness, a scale that contained items pertaining to the nature and quality of multiagency collaborations. The outcome was measured by asking respondents to report on 13 different actions that officers may take within their agency. Each item was coded 0 if the agency did not employ the action, and 1 if it did. These 13 items were summed to create an index ranging 0–13 (Cronbach's *α* = .815). Only five of the 13 items were indicative of multiagency partnerships and therefore eligible for the review. These are: interagency taskforce participation ($\overset{®}{X}$ = 0.516), procedures for contacting other authorities ($\overset{®}{X}$ = 0.592), mutual aid agreements with law enforcement agencies ($\overset{®}{X}$ = 0.790), mutual aid agreements with nonlaw enforcement agencies ($\overset{®}{X}$ = 0.458) and operating on a shared radio frequency ($\overset{®}{X}$ = 0.910). It should be noted that because the items were summed into a single composite scale, the study cannot disentangle the impact of the influence of homeland security, private, community or corporate grants on multiagency working from the other ineligible outcomes in the preparedness scale. The results of this study must therefore be considered with caution in the context of this systematic review.

Stewart and Oliver (2014) conducted a survey of 208 Texan police chiefs in 2007 to examine homeland security initiatives since September 11, 2001. A total of 271 police chiefs were invited to participate in the study, 242 agreed to complete the survey, and 208 provided useable data. The authors report that this sample represents approximately 20% of all police chiefs in Texas. Few demographic details were reported by the study authors, aside from the nature of the police chiefs' roles and experiences with emergency events (including terrorist incidents). Specifically, most respondents oversaw small or very small police departments (82.4%) in municipalities (75.5%) compared to school districts (15.4%) or universities (9.1%), and most of the respondents reported that they had no experience with emergency events (79.3%). For the purposes of this review, the eligible intervention variable was a dichotomous survey item that asked participants to indicate whether they had received homeland security funding/grants (1 = yes; no = 0). A total of 71 police chiefs reported receiving homeland security grants, compared to 137 who did not (comparison condition). The receipt of grants from homeland security was conceptualised as a police‐involved multiagency intervention because the police were partnering with funding agencies to enact strategies to counter violent radicalisation. The study used a zero inflated negative binomial regression model to assess the impact of receiving homeland security grants on an eligible multiagency outcome, controlling for the effect of multiple key policing variables. The eligible outcome was the extent of homeland security initiatives implemented by the police departments, a scale that contained several items pertaining to the nature and quality of multiagency collaborations. The outcome was a summed composite scale comprised of 14 dichotomous items requiring police chiefs to indicate whether they had implemented any of the homeland security measures. Each item was coded 0 if the agency did not employ the action, and 1 if it did. Only six of the 14 items were indicative of multiagency partnerships and therefore eligible for the review. The items in the homeland security initiative scale including (eligible items indicated by asterisk):

- “Ensured interoperable radio emergency communications with agencies outside jurisdiction*
- Personnel assigned to a FBI‐led Joint Terrorism Task Forces*
- Ensured interoperable radio emergency communications with agencies within jurisdiction
- Changed mission statement to reflect homeland security responsibilities*
- Adopted the National Incident Management System*
- Reassigned personnel to counterterrorism/homeland security functions*
- Signed/updated formal mutual aid agreements with other jurisdictions (since 9/11)*
- Formed an intelligence unit focused on counterterrorism
- Initiated, expanded, and/or participated in disaster response exercises
- Formed an counterterrorism unit other than criminal intelligence counterterrorism
- Conducted a local risk assessment
- Changed deadly force policies
- Linked offence report system to Texas Data Exchange^7^
- Broadened the role of an existing intelligence unit” (p. 7)

Respondents reported that the most frequently used initiative by their agency was interoperable radio communications with agencies outside of their jurisdiction (*n* = 62, 87.3%), followed by adopting the National Incident Management System (*n* = 60, 84.5%). Just over three quarters of agencies had signed or updated formal mutual aid agreements (*n* = 54, 76.1%), while just over one tenth assigned personnel to Joint Terrorism Task Forces (JTTF; *n* = 8, 11.3%) or had changed their mission statement to reflect homeland security responsibilities (*n* = 8, 11.3%). The least common initiative of the eligible measures was reassignment of personnel to counterterrorism/homeland security functions (*n* = 7, 9.9%). For the purposes of this review, the composite scale is considered an eligible outcome measure because it does contain key multiagency outcome items. It should be noted that because these items were summed into a composite scale, the study does not disentangle the impact of partnering with homeland security via grants on multiagency working from the other ineligible outcomes within the scale. The results of this study must therefore be considered with caution in the context of this systematic review.

##### Review of mechanisms, moderators, implementation, and economic considerations (Objective 2)

Of the 181 studies deemed eligible for this component of the review, 26 (14.36%) met a threshold of at least 3 on at least one of the mechanisms, moderators, implementation and economic domains using the EMMIE appraisal tool (Johnson et al., 2015; Tompson et al., 2015). Table 3 provides a brief summary of all 181 studies deemed eligible for this review, including the 155 studies that did not reach a rating of 3 on the EMMIE appraisal tool. This table details brief coding on the document type, data collection tools, participants, intervention and partners of each study, alongside the ratings for each study on each of the MMIE domains. The studies encompass a variety of publication types, including peer‐reviewed journal articles (*n* = 73), dissertations (*n* = 55), technical research reports (*n* = 40), and books or chapters in edited books (*n* = 13). Data collection tools were overwhelmingly qualitative with 119 of 181 (65.75%) studies utilising semistructured interviews, observations, case studies and document analysis. A total of 37 (20.44%) used quantitative data and methods only with no supplementation with qualitative evidence. The remaining 25 studies (13.81%) utilised mixed qualitative and quantitative data and methodologies. In terms of partners, most (*n* = 128) were agencies or organisations, with smaller representation from community patterns (*n* = 18) and a mixture of community and agency/organisational partners (*n* = 35). The intervention models varied across the studies, including fusion centres, community policing, training, multiagency information sharing, Prevent/CONTEST model (UK), and multiagency task‐forces, teams or expert panels.

**Table 3:** Overview of studies with a rating <3 on EMMIE appraisal tool

Study	Document type	Data collection tools	Participants	Intervention	Partners	Rating for mechanisms	Rating for moderators	Rating for implementation	Rating for economics
Ablah et al. (2010)	Journal article	Quantitative survey	Health department, fire department, fire marshal, fire and EMS academies, police and sheriff, hospitals, and New York consortium for Emergency preparedness continuing education (*n* = 1181)	Operation RAPID (simulated multiagency response to anthrax bioterror threat). Points‐of‐dispensing (PODs) were multiagency ‐ included first responders/receivers and medical consultants	Agencies/organisations: health department, fire department, fire marshal, fire & EMS academies, police and sheriff, hospitals, and New York consortium for Emergency preparedness continuing education	0	0	2	0
Ahmed et al. (2018)	Book	Qualitative semistructured interviews, document analysis	Community organisation reps (*n* = 29)	Law enforcement and community multiagency initiatives (in general, but some specific projects are discussed)	Agencies/organisations: community organisations	1	1	2	1
Anagnostakis (2017)	Book	Qualitative semistructured interviews and document analysis	US and EU officials (*n* = 13)	EU‐US cooperation since 9/11	Agencies/organisations: government agencies/stakeholders	1	1	1	0
Argomaniz (2011)	Book	Qualitative semistructured interviews, document analysis	Officials, policy‐makers and practitioners (“more than 40”, p. 2)	EU counterterrorism activities, including agency coordination	Agencies/organisations: no explicit entity, but references partner agencies	2	1	1	0
Atkinson (2019)	Journal article	Qualitative semistructured interviews	Representatives (*n* = 12) from agencies who work in partnership with the core five Scottish Crime Campus agencies (not directly specified, but could come from government bodies, universities, or community/faith groups)	Scottish Crime Campus, a “purpose‐built specialist accommodation” (p. 1) for law enforcement to collaborate around organised crime and terrorism	Mixed (community and organisations): government agencies (Crown Office and Procurator Fiscal Service, UK National Crime Agency, HM Revenue & Customs), university/academic establishments, community/faith groups	1	1	2	0
Australian National Audit Office (2010)	Report	Audit (qualitative semistructured interviews, field observations, and document analysis)	Attorney‐General's Department, AFP, ASIO staff and documents (*n* = NR)	National Security Hotline	Agencies/organisations: Attorney‐General's Department (manages operation of NSH), Australian Security Intelligence Organisation (ASIO), Australian Federal Police (AFP), and state/territory police agencies	0	0	3	1
Awan (2012)	Journal article	Qualitative semistructured interviews	Community—Muslim community members from Alum Rock (*n* = 6)	Prevent (UK)	Community: Police, community leaders, Imam, local councillor	0	0	2	0
Aydinli and Yon (2011)	Journal article	Qualitative semistructured interviews	Police liaison officers (*n* not specified)	Police liaison officers, which do transnational police cooperation	Agencies/organisations: other officers, other countries' police, government	1	0	1	0
Bailey and Cree (2011)	Journal article	Quantitative survey	Police officers (*n* = 247)	“Collaboration among agencies” subscale (p. 438)	Agencies/organisations: agencies, not specified (p. 438)	1	1	0	0
Baldwin (2010)	Thesis	Quantitative survey	Law enforcement (*n* = 294)	Number of homeland security agency collaborations	Agencies/organisations: local governments, local military installations, state governments, FBI, Department of Justice, FEMA, Department of Defense, nongovernment agencies	0	2	1	0
Barton (2014)	Thesis	Qualitative semistructured interviews, qualitative survey instrument, field observations	United States Special Operations Forces commanders and members of the Assistant Secretary of Defense for Special Operations/Low‐Intensity Conflict (*n* = 15), US Special Operations Forces operations and intelligence personnel (*n* = 62)	Coordination and duplication of efforts of the two organisations with Afghan National Police	Agencies/organisations: United States Special Forces, Afghan National Police	0	1	2	0
Braziel et al. (2015)	Report	Qualitative semistructured interviews and focus groups, field observations, document analysis	Practitioners involved in the attack (police, FBI, victims, etc., see p. 6 for full list), over 200 ppl, specific *N* not reported	Response to terror attack in San Bernardino on December 2nd, 2015	Agencies/organisations: fire, emergency management, health, SWAT, etc. see p. 6	1	1	3	0
Brito et al. (2006)	Report	Working group observations	Police, fire, emergency medical services, emergency management, and public health agencies, government (*n* = NR)	Partnerships for planning and respondent to critical incidents	Agencies/organisations: fire, emergency medical services, emergency management, and public health agencies, government	1	1	2	0
Bullock and Johnson (2018)	Journal article	Qualitative semistructured interviews	Police officers and administrative staff (*n* = 12)	Interaction between police and Muslim faith groups, citizens, communities	Community: Muslim faith groups, community members	2	0	0	0
Burruss et al. (2010)	Journal article	Quantitative survey	Police officers (*n* = 1047)	Dependent variable is a scale that includes partner activities (i.e., “assigned staff to act as liaison to other agencies”, training, written protocols for contacting authorities;, p. 88)	Agencies/organisations: not specified beyond “other agencies” and “authorities”	2	0	0	0
Burruss et al. (2012)	Report	Quantitative survey	Chief executives from law enforcement agencies (*n* = 350)	Level of influence of grants from four partners (DHS, private industry, community, or corporate bodies)	Agencies/organisations: no explicit entity, but references partner agencies	1	3	1	1
Caeti et al. (2005)	Journal article	Qualitative semistructured interviews	Police officers (*n* = 11)	Response to Oklahoma city bombing	Agencies/organisations: media	1	1	1	0
Callaway et al. (2010)	Journal article	Mixed methods (survey + field observations)	Police officers and medical personnel (*n* = 16)	Responses to school terrorism events	Agencies/organisations: emergency medical services, school administrators	0	0	1	1
Campbell (2016)	Thesis	Quantitative survey	State police officers (state, highway patrol, and state bureaus of investigation; *n* = 60)	Allocation of funds/resources to intelligence gathering/analysis/sharing; and level of involvement in intelligence gathering/analysis/dissemination	Agencies/organisations: federal agencies (border patrol, customs, FBI, immigration)	0	2	2	1
Carter and Chermak (2012)	Book chapter	Quantitative survey	Regional and state Fusion Centre personnel (*n* = 96)	Fusion Centre	Agencies/organisations: other fusion centres, other law enforcement orgs (see p. 77 for list)	1	0	1	0
Carter (2006)	Thesis	Quantitative survey	Police officers (*n* = 175)	Intelligence toolbox training programme	Agencies/organisations: developed/delivered by university, FBI, and DEA	0	1	3	0
Carter (2014)	Journal article	Quantitative survey	Law enforcement personnel (*n* = 345) and fusion centre representatives (*n* = 96)	Information sharing practices and working relationships	Agencies/organisations: FBI, other law enforcement, other fusion centres, state government, Department of Corrections, hospitals and health, fire, homeland security, private sector	1	1	1	0
Carter et al. (2017)	Journal article	Mixed methods (survey + 3× in‐depth case studies based on visits to sites and semistructured interviews with centre staff)	Fusion centre personnel (*n* = 96) for survey + semistructured interviews with Centre Directors, Manager of Operations, Records Management personnel and 2× intelligence analysts	Fusion Centre	Agencies/organisations: emergency services, transportation services, private businesses	0	1	2	0
Carter et al. (2014)	Journal article	Quantitative survey	Law enforcement officers (*n* = 272)	National Criminal Intelligence Sharing Plan recommendations and DHS Target Capabilities List	Agencies/organisations: FBI, other federal LEAs, state LEAs, local LEAs, the state Fusion Centre, another state's Fusion Centre	3	1	2	0
Carver (2016)	Thesis	Qualitative semistructured interviews, document review and case studies	Law enforcement personnel (*n* = 21)	Information sharing and interagency coordination	Agencies/organisations: local and federal agencies	1	0	1	0
Castillo (2015)	Thesis	Qualitative semistructured interviews	Police officers (*n* = 10), community members (*n* = 8)	iWATCH antiterrorism programme	Community: community members involved in iWATCH program	0	0	1	0
Center on Global Counterterrorism Cooperation & IGAD (2012)	Report	Field observations and consultations with experts	Government officials, members of parliament, and officials from criminal justice, law enforcement, military, intelligence, diplomacy (*N* = NR)	Task force on Legal Cooperation Against Terrorism	Agencies/organisations: no explicit entity, but references partner agencies	1	1	2	0
Cetin (2010)	Thesis	Qualitative semistructured interviews	US (*n* = 10), Turkey (*n* = 13) officials from police, ministries, prosecutors, attorneys	Witness security programs	Community: witnesses	1	0	2	0
Cherney (2018)	Journal article	Qualitative semistructured interviews	Community Liaison Team members (sworn and civilian AFP; *n* = 8)	Community Liaison Team	Community: Muslim community members	2	0	1	0
Clarke (2006)	Thesis	Qualitative semistructured interviews	Police (*n* = 12)	Interagency connectedness	Mixed (community and organisations): other law enforcement, communities	2	0	2	0
Clément (2017)	Journal article	Document analysis	Documentation from Royal Canadian Mounted Police (over 50,000 pages)	Planning and security operations for Olympics	Agencies/organisations: security service, foreign service personnel, Olympics committee	0	0	2	0
Comens (2016)	Thesis	Quantitative survey	Law enforcement agencies (*n* = 902)	Counterterrorism preparedness, including training, partnerships with other agencies	Agencies/organisations: no explicit entity, but references partner agencies	0	1	0	0
Cordner and Scarborough (2010)	Journal article	Quantitative vignette survey	Experts (police executives, military intelligence, federal law enforcement, and academic experts, *n* = 14)	Who and how each potential terrorist scenario would be handled by authorities (often involving multiagency and information sharing between LE and other govt. agencies). Mostly expert recommendations or broader level implementation detail rather than play by play	Agencies/organisations: Intelligence agencies, military, FBI	0	0	1	0
Council of State Governments (2006)	Report	Mixed (survey, case studies, working group)	Law enforcement agencies (*n* = 400)	Intelligence gathering, sharing and analysis (range of practices)	Agencies/organisations: homeland Security, FBI, various other entities	1	0	1	0
D'Souza (2009)	Thesis	Mixed (qualitative semistructured interviews, quantitative surveys)	Reporting entities, law enforcement, politicians, bureaucrats, subject matter experts	Financial Intelligence Units—which receive info, analyse it appropriately, and then disseminate it to the appropriate agency	Agencies/organisations: reporting agencies, domestic agencies	0	0	2	1
Davies and Murphy (2004)	Report	Working group observations	Federal, state and local law enforcement executives, federal law enforcement professionals, diverse community leaders and criminal justice practitioners (*n* = NR)	Community policing	Mixed (community and organisations): FBI, community	1	0	2	0
Davies and Plotkin (2005)	Report	Working group observations	Law enforcement executives (*n* = NR)	Law enforcement partnership with Department of Homeland Security	Agencies/organisations: DHS	1	0	2	0
Davis et al. (2010)	Report	Qualitative semistructured interviews, field observations, document analysis	Case study agencies (*n* = 5)	Fusion centres, information sharing	Agencies/organisations: funding bodies (e.g., DHS), fusion centre partners	1	1	2	0
Davis et al. (2016)	Report	Mixed (field observations, qualitative semistructured interviews, surveys, document analysis)	Training participants (police, *n* = 226), planners (*n* = 11), staff	State and Local Anti‐Terrorism Training (SLATT) program	Agencies/organisations: Bureau of Justice Assistance	2	0	3	3
De Londras and Doody (2015)	Book	Qualitative focus groups	Police officers, military (*n* = 7)	European border control databases	Agencies/organisations: other EU Member states/police	1	0	1	0
Dehnke (2011)	Thesis	Mixed (qualitative semistructured interviews, survey)	Police officers (*n* = 19 interviewees and *n* = 459 survey respondents)	Relationships between local and federal law enforcement, as well as whether agencies can implement anti‐terror policy	Agencies/organisations: other levels of law enforcement	1	0	2	0
Disley et al. (2016)	Report	Qualitative semistructured interviews	Police and probation officers, and persons from the Reducing Reoffending Group at the National Offender Management Service, *n* = 10	Multi‐Agency Public Protection Arrangements	Agencies/organisations: Probation Service, National Offender Management Service	1	1	2	0
Dorn and Levi (2009)	Journal article	Qualitative semistructured interviews	Public and private security personnel (*n* = 12)	“Trusted forum” for trading knowledge/expertise	Agencies/organisations: private security (e.g., information technology and communications, transport infrastructure security, chemical firms, public relations),and public security (judicial, supply chain, customs)	1	0	2	0
Dutcher (2011)	Thesis	Qualitative semistructured interviews and document analysis	Law enforcement (*n* = 18)	Guidelines for Homeland security (collaboration, information sharing)	Mixed (community and organisations): community (in general) and other law enforcement	2	0	1	0
Efrat (2015)	Journal article	Quantitative survey	Countries (*n* not reported)	Legal Attachés (Legats)—FBI‐run offices in another country to promote cooperation with that country's CT efforts	Agencies/organisations: International law enforcement agencies	1	2	0	0
Enders and Sandler (2011)	Journal article	Quantitative (official data)	Countries (*n* = 195)	MIND/FIND—technology for accessing Interpol databases remotely p. 266	Agencies/organisations: Interpol member countries	2	2	0	0
Fagersten (2010)	Journal article	Document analysis	Documents (*n* = NR)	Europol intelligence cooperation for counterterrorism	Agencies/organisations: governments	1	1	1	0
Favarel‐Garrigues et al. (2011)	Journal article	Qualitative semistructured interviews	Chief compliance officers at banks (*n* = 75), police, Banking Commission personnel, Tracfin agents, officials from the data protection agency	Partnerships with banks	Agencies/organisations: banks	2	1	1	0
Foster and Cordner (2005)	Report	Mixed (quantitative survey and case studies)	State police (*n* = 50)	Fusion Centre	Agencies/organisations: other levels of law enforcement, other fusion centre partners	0	0	1	0
Foster and Cordnerr (2005)	Report	Mixed (survey, case studies, working group)	Law enforcement agencies (*n* = 400)	Intelligence gathering, sharing and analysis (range of practices)	Agencies/organisations: Homeland Security, FBI, various other entities	1	1	2	0
Gale (2012)	Thesis	Qualitative semistructured interviews; field observations; publicly available quantitative data	From Oldham (Greater Manchester): Police officers (*n* = 8), Council members (Housing *n* = 1, anti‐social behaviour officer *n* = 1, neighbourhood management team member *n* = 1, communities unit *n* = 1), college staff members (*n* = 3), member of the media (*n* = 1), third sector community members/stakeholders (*n* = 3), community members (*n* = 21)	Neighbourhood Policing and National Intelligence Model	Mixed (agencies/organisations and community): local government (council) and community members/community organisation representatives	2	1	2	0
Giblin et al. (2014)	Journal article	Quantitative survey	Law enforcement agencies (*n* = 350)	Homeland security preparedness activities (includes multiagency such as mutual aid agreements and interagency task force participation)	Agencies/organisations: other law enforcement and nonlaw enforcement agencies	1	2	1	0
Graphia (2011)	Thesis	Qualitative semistructured, open‐ended interviews; qualitative field observations	Persons who worked/had worked within fusion centres internally (primarily involved in analytical processes and centre functioning/management), and external individuals (i.e., FBI representatives) who had been affiliated/worked with fusion centres (*N* = 49). Sample contained some sworn officers, previously sworn officers, and civilians	State fusion centres (collection, analysis and dissemination of intelligence)	Agencies/organisations: government agencies (DHS, FBI, local police departments, Department of Justice Bureau of Assistance, Immigrations and Customs Enforcement (ICE), U.S. Bureau of Alcohol, Tobacco and Firearms (ATF), and Federal Air Marshall Service), fusion centres (containing law enforcement and civilian employees), local and tribal partners, and private sector partners	1	1	2	0
Gresham (2018)	Thesis	Qualitative semistructured interviews, document analysis and field observations	Police officers and leadership (*n* = 13), police ride‐alongs (observations) =20. Document *N* = NR	Community partnerships, partnerships with other departments in the city and federally	Mixed (community and organisations): fire department, businesses, city departments, faith based organisations, shopping mall directors, health departments, community members	0	0	1	0
Gssime and Meines (2018)	Report	Field observations	P/CVE coordinators (*n* = 21)	Tabletop exercises using scenarios to prepare agencies for multiagency collaboration in the field	Mixed (community and organisations): mayors, local P/CVE coordinators, youth/family workers, community representative, prison and probation	1	0	2	0
Gunaratna et al. (2013)	Book	Qualitative semistructured interviews	Police officers, Muslim community groups, local authority reps, youth agency workers, policymakers; total *N* = 62	Police‐community engagement for counterterrorism	Mixed (community and organisations): Muslim community groups, other stakeholders ‐ local authority, youth agency workers, policymakers	2	1	1	0
Harwood (2017)	Thesis	Document (policy) analysis	Governmental documents, peer reviewed books and articles, policy documents (*n* = NR)	Standard Operating Procedures to incorporate collaboration	Agencies/organisations: airport staff	1	1	2	0
Henderson et al. (2006)	Report	Mixed (quantitative survey, qualitative semistructured interviews, focus groups)	Police personnel, FBI agents, community leaders (*n* = 209)	Police‐community interactions	Mixed (community and organisations): community, FBI	1	2	1	0
Hidek (2010)	Thesis	Qualitative semistructured interviews	Security planners (*n* = 28)	Lower Manhattan Security Initiative	Agencies/organisations: military, defense	1	1	2	0
Hirschfield et al. (2012)	Report	Mixed (qualitative semistructured interviews, administrative data)	Representatives from youth offending teams (*n* = 49), organisations (*n* = 38)	Range of programs within youth offending teams to reduce radicalisation of youth	Mixed (community and organisations): schools, mosques, local councils, other youth services, community groups	0	0	1	0
Holcomb (2017)	Thesis	Qualitative unstructured interviews	Law enforcement officers (*n* = 6)	Intelligence dissemination between local/state/federal agencies	Agencies/organisations: federal agencies	0	0	1	0
Holguin (2011)	Thesis	Comparative case study using qualitative semistructured interviews and document analysis	Local emergency managers, executive managers, and community representatives (Californian cities: Pasadena *N* = 6, Ontario *N* = 5, Anaheim *N* = 3)	Collaborative efforts in emergency management operations	Mixed (community and organisations): federal government, county government, private sector, NGOs, volunteer groups, religious groups	2	1	2	1
Hossan & Kuti (2010)	Journal article	Quantitative social network analysis	State law enforcement (*n* = 39), local law enforcement (*n* = 148), state emergency agencies (*n* = 37)	Disaster response preparedness networks	Agencies/organisations: other agencies at varying levels of law enforcement	1	0	1	0
Hutchinson et al. (2002)	Journal article	Qualitative observations	Emergency responders, managers, technical experts (state/local/fed level), *N* = “over 60” (p. 268)	5× 3‐day technical workshops on planning for bioterror response	Agencies/organisations: public health, fire, law enforcement, environmental protection, emergency management	0	0	2	0
Ibbetson (2011)	Thesis	Qualitative semistructured interviews	Police chiefs (*N* = 40)	Informal grape vine' and other community policing activities	Community: community members	1	0	1	0
Innes et al. (2017)	Journal article	Mixed methods (survey + field observations)	Police officers (*n* = 41); Community members “who had engaged with Prevent interventions in some fashion” (p. 255), *n* = 53.	Prevent (part of the UK “multi‐government CONTEST strategy”, p. 257, 262)	Mixed (community and organisations): government agencies (not further specified), community members	0	1	2	0
Institute of Race Relations (2010)	Journal article	Mixed methods (FOI requests, interviews, roundtable discussion)	Prevent programme managers, local authorities, members of Prevent boards, paid and voluntary personnel working on Prevent projects, community workers familiar with Prevent project (*n* = 32)	Prevent (UK)	Mixed (community and organisations): government agencies (not further specified), community members	0	1	2	1
Intelligence and Security Committee (2009)	Report	Document analysis	Operational documents, surveillance photographs, transcripts of conversations, police action logs and covert recordings (*n* documents = NR)	Operation CREVICE and 7/7/London bombing	Agencies/organisations: MI5	0	1	2	1
Isakjee and Allen (2013)	Journal article	Field observations, qualitative semistructured interviews, document analysis	“Institutional figures and campaigners” as well as “authorities”, p. 754	Project Champion, CCTV and automated numberplate recognition	Agencies/organisations: Birmingham City Council (to share running costs)	1	1	2	1
Jackson et al. (2002)	Report	Qualitative observations and written summaries of conference material	Firefighters, law enforcement, construction & other trade, health and safety, federal/state agencies (*n* = 150)	Three‐day conference for emergency service workers around PPE and terrorism	Agencies/organisations: National Institute for Occupational Safety & Health, emergency services organisations (local, state, federal, incl. police), trade unions, health & safety agencies, private sector equipment providers, govt., academic research	1	1	1	0
Jeffrey (2009)	Thesis	Qualitative semistructured interviews	Senior officers in RCMP (*n* = 5)	Interagency cooperation re borders	Agencies/organisations: other border agencies	1	0	0	0
Jenkins et al. (2014)	Report	Qualitative observations, focus group	Reps from: DHS, FBI, CIA, Department of Defence, first responder orgs, state/local law enforcement, state‐level homeland security agencies (*N* participants not disclosed)	Day‐long seminar on domestic intelligence and information sharing	Agencies/organisations: DHS, FBI, CIA, Department of Defence, first responder orgs, state/local law enforcement, state‐level homeland security agencies	1	0	1	0
Jiao and Rhea (2007)	Journal article	Qualitative semistructured interviews, document analysis	Police officers (*n* = 21)	Police integration—loose term describing reorganisation to combine multiple agencies' efforts for CT	Agencies/organisations: other levels of law enforcement	1	1	1	0
Johns et al. (2014)	Journal article	Qualitative semistructured interviews and focus groups	Community participants (*n* = 21), programme facilitators (*n* = 8), students (*n* = 10)	“More than a game” youth mentoring programme	Mixed (community and organisations): sporting associations, Islamic society, Attorney‐General's Department, AFP	1	0	1	0
Jones (2017)	Thesis	Quantitative survey	Law enforcement and security industry personnel (*n* = 412)	Federal Bureau of Investigation's National Improvised Explosives Familiarization/Chemical Industry Outreach Workshop	Agencies/organisations: FBI	1	0	2	1
Joyal (2012)	Journal article	Qualitative semistructured interviews	Fusion centre personnel (*n* = 49), sworn and administrative staff	Fusion centre	Agencies/organisations: FBI, National Guard and Department of Corrections, DHS, Department of Justice's Bureau of Justice Assistance (potentially others not mentioned, see p. 361)	1	1	1	0
Kaza et al. (2007)	Journal article	Quantitative modelling using real data	Police datasets	Mutual information—that is, combining police datasets to predict border crossings	Agencies/organisations: other law enforcement	1	0	0	0
Kerry (2007)	Report	Mixed (document analysis, surveys, interviews, see pp. 12–13)	Recipients of intervention (police), intervention managers, instructors, and stakeholders	Chemical, Biological, Radiological and Nuclear (CBRN) training for law enforcement	Agencies/organisations: Canadian Emergency Management College (CEMC) of Public Safety Canada (PS); the Public Health Agency of Canada (PHAC) and Health Canada (HC); the Royal Canadian Mounted Police (RCMP); Defence Research & Development Canada (DRDC); and the Canadian Nuclear Safety Commission (CNSC)	0	2	3	1
Kilianski et al. (2014)	Journal article	Field observations	Health authorities, medical reserve corps, fire personnel, community volunteers	Mass prophylaxis exercise in response to simulated bioterror threat	Mixed (community and organisations): health authorities, medical reserve corps, fire personnel, community volunteers	0	0	1	0
Kılıçlar et al. (2018)	Journal article	Mixed (survey (qualitative open‐ended questions and quantitative close‐ended questions), qualitative semistructured interviews)	Police and tourism directors (*n* = 162)	Practices to prevent terrorism in tourist destinations	Agencies/organisations: tourism managers	0	0	1	0
Kim and de Guzman (2012)	Journal article	Administrative data (one‐way ANOVA and multivariate regression)	State and local law enforcement agencies (*n* = 414)	Community oriented policing (including problem solving partnerships with citizens)	Community: citizen groups	0	2	0	0
Knight (2009)	Thesis	Qualitative semistructured interviews and field observations	Law enforcement professionals (*n* = 23), observations of meetings (*n* = 12)	Fusion Centre: Tampa Fusion Centre	Agencies/organisations: FBI, Immigration and Customs Enforcement, Customs and Border Protections, United States Secret Service, United States Marshals Service, United States Coast Guard, Department of Defense, Tampa International Airport.	1	1	3	1
Kundnani (2009)	Report	Qualitative semistructured interviews, document analysis, focus group	Stakeholders involved in Prevent (*n* = 32, Prevent managers/workers, community sector, voluntary sector)	Prevent programme activities	Mixed (community and organisations): local authorities, voluntary sector organisations	0	0	2	0
Lakhani (2012)	Journal article	Qualitative semistructured interviews	Community members, community leaders (e.g., religious leaders, imams), government minister, civil servants, local govt. employees, police officers, academics, researchers, *n* = 56	Prevent	Mixed (community and organisations): community members, community leaders, government employees, researchers/academics	0	1	2	1
Lamb (2012)	Thesis	Document analysis	Documentation (*n* = 14)	G20 Joint Intelligence Group—multiagency network to collect data and knowledge on individuals with the potential to disrupt the G20	Agencies/organisations: Royal Canadian Mounted Police, Ontario Provincial Police, Toronto Police Service, Peel Regional Police Service, Canadian Security Intelligence Service, Communications Security Establishment Canada, Canada Border Services Agency, and Canadian Forces	0	0	3	0
Lamb (2013)	Journal article	Qualitative semistructured interviews	Police officers (*n* = 16)	Prevent	Community: community members/groups	1	1	3	0
Lambert (2010)	Thesis	Qualitative semistructured interviews and participant observation	Muslim Contact Unit officers (police, *n* = 5), Brixton Salafis (*n* = 8), Finsbury Park Islamists (*n* = 8)	Muslim Contact Unit	Community: Brixton Salafis, Finsbury Park Islamists	2	1	2	0
Levy (2015)	Thesis	Qualitative semistructured interviews, document analysis	Security agency employees (*n* = 20)	Collaboration facilitators/barriers generally	Agencies/organisations: security agencies	1	0	1	0
Lewandowski (2012)	Thesis	Qualitative semistructured interviews and field observations	Employees of the ROIC (*n* = 12), observations over 18 months	Fusion Centre: New Jersey Regional Operations Intelligence Centre (ROIC)	Agencies/organisations: FBI, DHS, Alcohol, Tobacco & Firearms, Immigration & Customs Enforcement/Homeland Security Interventions, National Crime Information Centre, Customs and Border Patrol, Secret Service, Diplomatic Security Service, New Jersey State Police, New Jersey Office Of Homeland Security & Preparedness, New Jersey Department Of Corrections, New Jersey Probation & Parole, New Jersey Fire Service, New Jersey Treasury, Essex County Prosecutor's Office, Newark Police Department, Paterson Police Department, Camden Police Department, Ocean County Prosecutor's Office	1	1	3	0
Lewandowski et al. (2018)	Journal article	Quantitative survey	Fusion centre partners (*n* = 339)	Fusion Centre	Agencies/organisations: private sector, health, fire, emergency management services, other law enforcement	1	2	1	0
Lieberman (2009)	Thesis	Mixed (qualitative focus groups and quantitative survey)	Focus groups (*n* = 9) with participants from law enforcement and communities (*n* = 52 total)	Community policing	Community: community members and business owners	1	1	1	1
Loukaitou‐Sideris et al. (2006)	Journal article	Qualitative semistructured interviews, field observations	Transit managers and officials (*n* not reported)	Partnership with police around counterterror transit security	Agencies/organisations: transport officials/bodies	0	0	1	1
Lowe (2011)	Journal article	Covert participant observations	Officers from the Integrated Special Branch, *N* not stated due to nature of research methods	Surveillance by UK's Integrated Special Branch, incl. info sharing	Agencies/organisations: other ISBs, other agencies, e.g., Benefits Agency or National Health Service	1	1	2	0
Loyka et al. (2005)	Report	Working group observations	Federal, state, and local law enforcement officials, intelligence experts, and academics from the intelligence and criminal justice fields. *n* = NR)	Intelligence and information sharing in community policing	Agencies/organisations: FBI, other entities not explicitly mentioned	1	0	2	0
Lyons (2002)	Journal article	Qualitative semistructured interviews, participant observation, document analysis	Community members, police (*N* not specified)	Community policing	Community: community activists	1	0	2	0
Mabrey et al. (2006)	Journal article	Participant observation	Stakeholder reps: university staff/researchers, police	Development of the Border Intelligence Networks Project (training for police officers)	Agencies/organisations: universities	1	0	3	1
MacPherson (2006)	Thesis	Qualitative semistructured interviews	Representatives from local security network (e.g., coast guard, intelligence units, RCMP, *n* = 10)	Multiagency security network	Agencies/organisations: border security, coast guard, immigration services, government departments (e.g., Department of Fisheries), port authorities	2	1	2	0
Madsen (2013)	Thesis	Qualitative semistructured interviews, document analysis	Law enforcement (*n* = 6)	Interagency cooperation, JTTF, fusion centres (general discussion)	Agencies/Organisations: Other govt. agencies involved in counterterrorism	1	0	1	1
Manley and Bravata (2009)	Journal article	Qualitative focus groups	Civilian and military officials (*N* not reported), comprising public health, emergency management, hazardous materials response, law enforcement, military health and emergency management	Bioterrorism preparedness	Agencies/Organisations: Public health, emergency management, hazardous materials response, military health & emergency management	1	1	2	0
Manning (2006)	Book chapter	Qualitative ethnographic case studies	Practitioners comprising the case studies (federal and local police, Military (Air Force, Army, Coast Guard), other first responders (Fire, Emergency))	Temporary multiagency network to prepare and manage terrorist risk for large public events	Agencies/organisations: military (Air Force, Army, Coast Guard), other first responders (Fire, Emergency)	0	1	2	1
Marion and Cronin (2009)	Journal article	Quantitative survey	Police chiefs (*n* = 260)	Departmental changes (interagency communication, training, grants)	Agencies/organisations: other enforcement agencies, government	1	0	2	1
Marks and Sun (2007)	Journal article	Document analysis	Police agencies	Organisational change oriented toward increased interaction with other agencies and community	Mixed (community and organisations): federal, state & local police agencies, community	0	0	1	0
Marks (2014)	Thesis	Qualitative semistructured interviews	Fusion centre personnel	Fusion centre	Agencies/organisations: fusion centre partners	1	1	2	0
Matusitz (2006)	Thesis	Qualitative semistructured interviews	Computer security experts and law enforcement personnel (*n* = 27)	Partnerships and networks of computer security labs, law enforcement and FBI for addressing cyberterrorism	Agencies/organisations: computer security labs, universities, FBI	2	1	0	0
McDonagh (2013)	Thesis	Mixed methods (qualitative semistructured interviews, focus groups, quantitative questionnaires, document analysis, and quantitative and qualitative questionnaires)	Interviews—experts (Government officials, senior police officers including Assistant and Deputy commissioners, journalists, and key person in case study. total *N* = 9); 1 in‐depth case study; Focus groups (police officers ranked constable, sergeant, and inspector; held in four locations; total *N* (focus groups) =NR); Questionnaires—Used as both prompts for focus groups (*N* = NR); Document analysis (*N* = NR)	Counter terrorism stop and search powers in the UK	Mixed (community and organisations): Community Monitoring Network, unions, Directorates of legal services and public affairs, Muslim Safety Forum, Home Office	0	1	1	0
McQuade (2016)	Thesis	Qualitative semistructured interviews and ethnographic fieldwork	Administrators, police officers, intelligence analysts, and other domestic intelligence employees (*n* = 75)	Fusion centres	Agencies/organisations: Fusion Centre partners	2	2	2	0
McRae (2010)	Journal article	Qualitative case study, field observations	Police and vocational programme participants	Police‐led and ‐designed reintegration programs for former combatants—in the form of vocational training	Agencies/organisations: government, nongovernment organisation	2	2	1	1
Mesloh et al. (2003)	Journal article	Field observations	University staff, PhD students, and campus police (*n* = 3)	Amnesty boxes	Agencies/organisations: universities	1	0	3	1
Mitchell (2016)	Journal article	Qualitative semistructured interview and document analysis	Policy officials, police, industry reps, media (*n* not reported)	Sharing of counterterrorism info between authorities and media	Agencies/organisations: media	0	1	1	0
Monahan and Palmer (2009)	Journal article	Document analysis	Document analysis of news articles	Fusion centre	Agencies/organisations: fusion centre partners	1	1	2	1
Stewart and Mueller (2014)	Journal article	Cost‐benefit analysis	Police (multiple levels), customs and border security, staffing reported in Table 1 (*n* = 82 for the two joint interventions).	Joint airport investigation teams, Joint airport intelligence groups	Agencies/organisations: Customs & Border Protection Service, Federal, state police.	0	0	1	3
Mullins (2016)	Journal article	Qualitative semistructured interviews	Counterterrorism police officers (*n* = 11)	Joint Counterterrorism Teams	Agencies/organisations: other police, ASIO, joint counterterrorism team members	1	1	1	0
Murphy and Plotkin (2003)	Report	Working group	Police personnel, FBI, Department of Justice, private practitioners, District Attorney's office (*n* = 33)	Meeting/working group to discuss practice for local/federal partnerships	Mixed (community and organisations): citizens, FBI, government	1	1	1	0
Nolan et al. (2003)	Journal article	Observations	Law enforcement (*n* = 400)	Planning and response to anthrax bioterrorism threat	Agencies/organisations: physicians, emergency departments, clinical laboratories	0	0	1	0
Odabasi (2011)	Thesis	Mixed (quantitative survey, qualitative semistructured interviews)	Law enforcement (*n* = 11 interviewees and *n* = 40 survey respondents)	Fusion centre	Agencies/organisations: fusion centre partners	1	1	1	0
Office of Community Oriented Policing Services (2004)	Report	Working group observations	Local, state, and federal law enforcement executives; public health and fire officials and subject matter experts (*n* = NR)	Collaboration around bioterrorism	Agencies/organisations: first responders	1	0	2	0
Onyango (2018)	Thesis	Qualitative semistructured interviews and document analysis	National Counter‐Terrorism Centre security personnel and Kenyan Police Service Gazetted Officers (*n* = 4), Content analysis of newspapers and government documents (*n* = 150)	Terrorism Amnesty Reintegration Program (ARP)	Agencies/organisations: National Counter‐Terrorism Centre of Kenya (multiagency group including state agencies, collaborates with nonstate agencies).	2	1	3	0
Ors (2011)	Thesis	Qualitative semistructured interviews	Mid‐level police managers (*n* = 62)	Interpol, Europol, SECI and Turkey's bilateral police cooperation with other countries	Agencies/organisations: no explicit entity, but references partner agencies	0	0	1	0
Ortiz et al. (2007)	Journal article	Qualitative semistructured interviews	Local law enforcement (*n* = 38), FBI (*n* = 16), community leaders (*n* = 53)	Multiple discussed—Joint Terrorism Task Force, interagency collaboration, info sharing	Mixed (community and organisations): FBI, other law enforcement, community groups	0	1	1	0
Ozguler (2008)	Thesis	Qualitative semistructured interviews, document analysis and participant observation	Law enforcement experts (*n* = 15)	External learning opportunities—personnel exchange with partners (e.g., banks)	Agencies/organisations: customs, banks, other law enforcement entities (e.g., in other countries)	1	0	2	0
Paripurna (2018)	Thesis	Qualitative semistructured interviews and document analysis	Counterterrorism police, academics, members of parliament, human rights activists, and former jihadists (*n* = NR), cases and policy documents	Intelligence‐led policing and collaborations	Agencies/organisations: state intelligence agency (Badan Intelijen Negara), armed forces (BAIS)	1	0	2	0
Pelfrey (2007)	Journal article	Quantitative survey	Law enforcement (from police departments, sheriffs departments, state agencies including highway patrol, *n* = 171)	Multiagency counterterrorism training	Agencies/organisations: emergency services, fire, hospitals, other law enforcement	1	0	0	0
Pickering et al. (2007)	Report	Mixed (semistructured interviews, surveys, and document/legislation review)	Police officers (*n* = 581)	Community policing	Mixed (community and organisations): community members, other agencies	2	1	2	0
Pickering et al. (2008a)	Journal article	Mixed methods (legislative analysis, surveys and interviews with police, interviews/focus groups/public forums with communities)	Police (*n* = 541; 18 specialist police) and community members (*n* = 42 individuals; focus groups, public forums)	Community policing	Mixed (community and organisations): government agencies (not further specified), community members	1	1	2	0
Pickering et al. (2008b)	Book	Mixed (qualitative semistructured interviews, survey)	Police officers (*n* = 50 interviewees and *n* = 541 survey respondents), community members (“more than 500”, p. 113)	Community policing	Mixed (community and organisations): community members, other agencies	1	2	1	0
Police Executive Research Forum (2017)	Report	Forum observations and case studies	Police leaders and community partners (*n* = NR)	Forum on Building Interdisciplinary Partnerships to Prevent Violent Extremism.	Agencies/organisations: no explicit entity, but references partner agencies	1	1	2	0
Power and Alison (2017)	Journal article	Observations (decision logs from exercises), quantitative questionnaire (descriptive)	Commanders from police, fire and emergency, and ambulance services (*n* = 50)	Simulated multiagency response to terrorist firearm attack	Agencies/organisations: fire & rescue, ambulance services	1	1	2	0
Presnell (2008)	Thesis	Quantitative survey	Law enforcement agencies (*n* = 186)	Resource allocation to multiagency activities and partnership with federal agencies	Agencies/organisations: FBI, FEMA, other entities not explicitly named	0	2	0	0
Rabbit (2009)	Thesis	Quantitative survey	Police officers (*n* = 64)	Collaboration with Homeland Security	Agencies/organisations: homeland Security	0	2	2	0
Ramirez and Quinlan (2012)	Report	Qualitative focus groups and semistructured interviews	Law enforcement and community members (from Mosque, volunteer group), *N* not reported	Community policing	Mixed (community and organisations): community members (from Mosque, volunteer group)	0	1	1	0
Ramirez (2012)	Report	Qualitative semistructured interviews, focus groups and written communications (e.g., email)	Law enforcement personnel and community members (*n* = NR)	Partnering for Prevention and Community Safety Initiative	Mixed (community and organisations): Muslim, Arab and Sihk communities, university	1	1	2	0
Ramsey (2012)	Thesis	Qualitative semistructured interviews, document analysis	Police training officers (*n* = 7)	Homeland security information sharing and voluntary additional training	Agencies/organisations: homeland Security	1	1	2	0
Randol (2013)	Journal article	Quantitative survey	Police agencies (*n* = 816)	Terrorism response plan with interagency planning	Community: citizen groups	2	1	2	0
Regan et al. (2015)	Journal article	Qualitative semistructured interviews	Fusion centre reps, industry partners, civil society groups (p. 758, *n* = 56)	Suspicious Activity Reports (SARs) use in fusion centres	Agencies/organisations: fusion centre reps, industry partners, civil society groups	0	1	2	0
Ryan (2006)	Thesis	Qualitative semistructured interviews	Intelligence agency personnel (*n* = NR)	Cooperation and information sharing at EU level	Agencies/organisations: EU member states and their intelligence agencies	1	1	1	0
Sandler et al. (2011)	Journal article	Cost‐benefit analysis	Interpol member countries	Interpol proactive coordination of CT efforts	Agencies/organisations: Interpol member countries	0	1	0	3
Sandoval (2013)	Thesis	Quantitative survey	Diplomatic, Intelligence/Information, Military, Economic, Homeland Security, and Law Enforcement (*n* = 180)	Information sharing	Agencies/organisations: the Departments of Defense, State, Energy, Homeland Security, Justice, Transportation and Commerce; the National Archives and Records Administration; the Office of the Director of National Intelligence; the Program Manager‐Information Sharing Environment; the National Maritime Domain Awareness Coordination Office (NMCO); the Army‐hosted, INTELST intelligence sharing forum; Booz Allen Hamilton; Armed Forces Communication and Electronics Association; the National Industrial Security Program Policy Advisory Committee; and the National Defense Intelligence College (NDIC).	2	2	3	0
Schaible and Sheffield (2012)	Journal article	Quantitative survey	Law enforcement agencies (*n* = 61)	Involvement in homeland security‐related intelligence and intelligence gathering, analysis and sharing	Agencies/organisations: international (e.g., border patrol, CIA), financial (e.g., secret service, IRS), aviation (e.g., US air marshals), and disaster management (e.g., FEMA).	0	1	1	0
Schanzer et al. (2016)	Report	Mixed (quantitative survey, qualitative semistructured interviews and field observations)	Municipal and county law enforcement officers (*n* = 19 telephone interviews and *n* = 50 interviews during site visits) and Muslim American communities (*n* = 200).	Police‐community partnerships and outreach.	Community: Muslim groups, churches, and social services	1	1	3	0
Sedevic (2012)	Journal article	Quantitative survey	Law enforcement recruits (*n* = 133)	“Emergency Response Week” component of recruit training curriculum	Agencies/organisations: Fire department, SWAT, Critical Incident Response Team	1	0	2	0
Sevinc and Guler (2006)	Journal article	Qualitative semistructured interviews and document analysis	Police officers (*n* = 37)	Community policing	Community: Community members	1	1	1	0
Shultz (2009)	Thesis	Qualitative semistructured interviews and document analysis	Members of enforcement teams and other elite police officers (n = NR)	Integrated border enforcement teams	Agencies/organisations: International law enforcement, border enforcement agencies	1	1	2	0
Simon (2012)	Journal article	Ethnographic field work	Researcher (*n* = 1)	Street policing of the public engaging in potentially suspicious behaviours: photography	Agencies/organisations: DHS	0	1	0	0
Spalek and McDonald (2010)	Journal article	Qualitative semistructured interviews	Police officers (Muslim Contact Unit, National communities Tension Team, or Association of Chief of Police Officers), *N* = 13, and members of Muslim communities or organisations, *N* = 29	Police partnership with Muslim communities	Community: Muslim community members and organisations	1	1	1	0
Spalek (2010)	Journal article	Qualitative semistructured interviews and participant observation	Police officers (Muslim Contact Unit, National communities Tension Team, or Association of Chief of Police Officers), *N* = 13, and members of Muslim communities or organisations, *N* = 29	Community policing	Community: Muslim community members and organisations	2	1	1	0
Spalek (2013)	Book chapter	Qualitative semistructured interviews	Police officers (*n* = 15); Muslim community groups (*n* = 14); Other stakeholders (*n* = 13); Muslim youth (*n* = 9 + 2 focus groups, 6 participants in each group)	Police‐community partnerships to “prevent extremism amongst Muslim youth” (p. 58)	Mixed (community and organisations): Muslim communities and other stakeholders (e.g., youth agency workers, policy makers)	1	1	2	0
Spalek et al. (2008)	Report	Qualitative semistructured interviews, document analysis and participant observation	Police officers (*n* = 13) and community members (*n* = 29)	Muslim Contact Unit and Muslim Safety Forum	Community: members of Muslim communities and organisations	1	1	2	0
Stalcup (2009)	Thesis	Qualitative semistructured interviews and field observations	Not clearly specified, includes police officers	Fusion centres	Agencies/organisations: fusion centre partners, e.g., National Guard	0	0	1	0
Stewart and Mueller (2018)	Journal article	Cost‐benefit analysis	Police, other airport security personnel (see partners column), *N* not specified.	Joint Terrorism Task Force; Visible Intermodal Protection Response teams	Mixed (community and organisations): JTTF includes FBI, police, and public. VIPR includes Federal Air Marshals, transport security, behaviour detection officers, explosive specialists, airport officials, & law enforcement	0	1	1	3
Stewart and Oliver (2014)	Journal article	Quantitative survey	Police chiefs (*n* = 208)	Homeland security grant funding	Agencies/organisations: homeland Security	1	1	2	0
Straub et al. (2017)	Report	Qualitative semistructured interviews, focus groups, incident data review, document analysis)	local, state, and federal law enforcement and first responder including dispatchers, officers and deputies, and public safety executives and managers; city, state, and federal officials; medical responders; a survivor; and community and faith leaders (*n* = NR)	Response to the Pulse Nightclub terror attack	Agencies/organisations: FBI, other state/local police departments, fire and rescue departments, Bureau of Alcohol, Tobacco, Firearms, and Explosives, DHS, Customs & border patrol, immigration & customs enforcement, DEA, US Department of State, Internal Revenue Services, Office of the United States Attorney (Middle District of Florida)	2	1	2	0
Streichert et al. (2005)	Journal article	Quantitative survey	Public health practitioners and first responders from the police and fire departments (p. 896, *n* = 113)	Cross‐disciplinary problem‐based‐learning course for multiple professionals involved in responding to bioterrorism	Agencies/organisations: public health, fire fighters, emergency medical services, emergency management, hospital administration	2	0	2	0
Strom and Eyerman (2007)	Journal article	Qualitative semistructured interviews	Law enforcement and public health practitioners (*n* = 42)	Interagency coordination, not otherwise explained	Agencies/organisations: public health officials	2	0	1	0
Tatil (2011)	Thesis	Qualitative semistructured interviews	Counterterror police (*n* = 3)	Information sharing	Mixed (community and organisations): see p. 72, includes other police departments, other cities, media, dispatch, community members, and so forth	0	0	2	0
Taylor and Kaufmann (2009)	Journal article	Qualitative case study	Ports (*n* = 17)	Inter‐organisational relationships at ports	Agencies/organisations: public and private officials at local, state, federal level (not specified beyond this, p. 33)	1	0	2	0
Taylor et al. (2012)	Book	Qualitative semistructured interviews and field observations	Key personnel from police and Australian Security Industry Association (*n* = 12)	Police‐private security partnerships: Project Griffin, Qantas Security, Eyes on the Street	Agencies/organisations: private security	0	0	1	0
Thacher (2005)	Journal article	Qualitative semistructured interviews, field observations, document analysis	People involved in Arab American institutions, mayor, police chief, police command staff, community policing officers; *N* not specified	City's responses to hate crimes following 9/11 and subsequent media attention, local homeland security office (p. 646)	Agencies/organisations: Arab American institutions, mayor, other government	1	1	2	0
Thompson (2017)	Thesis	Quantitative survey	Law enforcement agencies (*n* = 102)	Contingency plan designed for multiagency use (p. 45), counterterrorism training, “resource level” (measured by interdepartmental information exchange)	Agencies/organisations: county, municipal, state, federal agencies	0	2	1	0
Tregidga (2011)	Thesis	Mixed (quantitative document analysis, qualitative semistructured interviews, document analysis, field observations)	Police of varying ranks (*n* = 39)	Channel project (part of Prevent/CONTEST)	Mixed (agencies/organisations and community): statutory, voluntary and community sectors	1	0	2	1
U.S. Department of Homeland Security (2015)	Report	Mixed methods (qualitative semistructured interviews, document analysis, self‐assessment tool, surveys)	Fusion Centres (*n* = 78), other agencies (*n* = 43) and fusion centre customers (*n* = 199)	Fusion Centre	Agencies/organisations: Fusion Centre implementers and partners (e.g., law enforcement, FBI, Federal Emergency Management Agency).	1	0	3	1
U.S. Department of Homeland Security (2016)	Report	Quantitative survey	Fusion centres (*n* = 77), “key customers” of fusion centres (n = 158)	Fusion Centre	Agencies/organisations: law enforcement, emergency operations centre, homeland security, fire, national guard, maritime, customs, and more.	1	0	3	1
Udo‐Akang (2013)	Thesis	Qualitative open‐ended survey responses	Homeland Security subject matter experts (*n* = 20)	Homeland Security—institutional consensus among agencies	Agencies/organisations: no explicit entity, but references partner agencies	0	0	1	0
United Nations Office on Drugs and Crime (2010)	Report	Working group (field observations and document analysis)	Law enforcement, judicial, and prosecution experts	International cooperation initiatives for counterterrorism	Agencies/organisations: law enforcement, security services, international bodies, governments	1	1	2	0
United States Senate Committee on Homeland Security and Governmental Affairs (2012)	Report	Mixed (qualitative semistructured interviews, quantitative surveys, and document analysis)	Federal, state and local officials (*n* = NR)	Fusion centres	Agencies/organisations: Fusion Centre partners	0	0	2	1
US Government Accountability Office (2012)	Report	Document analysis and qualitative semistructured interviews	Government reports (*n* = NR), senior level law enforcement (*n* = NR)	Watchlist nominations processes	Agencies/organisations: Department of Homeland Security (DHS); Department of Justice's Federal Bureau of Investigation (FBI) and Terrorist Screening Centre (TSC); Department of State (State); Department of Defense; Office of the Director of National Intelligence's National Counterterrorism Centre (NCTC); Central Intelligence Agency (CIA); and Executive Office of the President's National Security Staff	0	1	1	0
van den Heuvel et al. (2012)	Journal article	Observations (“decision logs”)	Police officers (high ranking, UK) and reps from Home Office, Security Service and Ministry of Defence (*n* = 136)	SAFE‐T model for decision making during CT event	Agencies/organisations: Home Office, Security Service & Ministry of Defence	2	1	3	0
Vidal (2013)	Thesis	Qualitative semistructured interviews and case study	Police officers and civilian intelligence analysts (*n* = 12)	Information sharing and fusion centres	Agencies/organisations: other LEAs at varying levels, fusion centre	1	0	1	0
Waldman et al. (2012)	Journal article	Qualitative ethnographic case studies	Israeli Defence Force captain (*n* = 1)	Tactical Medicine (TAC‐MED)	Agencies/organisations: SWAT teams × 3 (breaching team, secure perimeter, backup & cover), Medical force deployment × 2 (team A physicians & combat medics, team B paramedic & combat medics)	0	1	2	0
Walsh (2011)	Thesis	Mixed (quant survey and semistructured interviews)	Police (federal, state, local), *n* = 32	Training for LEOs	Agencies/Organisations: Differing agency levels (state, federal, local), or private agencies	0	2	2	0
Waters (2011)	Thesis	Qualitative semistructured interviews; phenomenological study	Professionals & experts in antiterrorism (*n* = 17, includes federal judge, federal agent, federal prosecutor)	DHS/police partnership	Agencies/organisations: DHS, FBI, state departments	0	0	2	0
Weine and Braniff (2015)	Report	Working group observations	Federal, state and local, international, and nongovernmental entities (*n* = NR)	National Summit on Empowering Communities to Prevent Violent Extremism (community policing models were discussed)	Mixed (community and organisations): federal government, and community stakeholders from mental health, social work, religious groups, education	1	1	1	0
Weine and Braniff (2017a)	Book chapter	Qualitative thematic analysis	Federal, state, local and international and NGOs “engaged in CVE efforts” (p. 453), and six UA community representatives	Two‐day summit to facilitate multiagency partnerships for CVE (US‐focused)	Mixed (community and organisations): federal, state, local and international agencies, NGOs “engaged in CVE efforts” (p. 453), and 6 UA community representatives	0	1	2	1
Weine and Braniff (2017b)	Report	Working group observations	Federal, state, local, international, and nongovernmental representatives (*n* = NR)	National Summit on Empowering Communities to Prevent Violent Extremism (community policing models were discussed)	Mixed (community and organisations): federal government, and community stakeholders from mental health, social work, religious groups, education	1	1	1	0
Weine and Younis (2014)	Book	Qualitative semistructured interviews and field observations	Police officers, community leaders/advocates, parents, youth, *n* = 70	LAPD's liaison section of the Counter‐Terrorism & Special Operations Bureau (community policing)	Mixed (community and organisations): government stakeholders, faith‐based orgs, other private/public orgs, nongovernment orgs	0	0	3	0
Weine et al. (2017)	Report	Qualitative semistructured interviews and field observations	Community police (*n* = 15), community members (*n* = 90)	Community policing	Agencies/organisations: healthcare organisations, faith‐based organisations, government, nongovernment organisations, and academic institutions (i.e., schools and universities)	1	1	3	0
Whelan (2012)	Book	Qualitative semistructured interviews	Security, law enforcement and intelligence personnel (*n* = 20).	Multiagency counterterrorism networks	Agencies/organisations: ASIO, customs/border control, AFP, government departments	2	1	2	0
Whelan (2016)	Journal article	Qualitative semistructured interviews	Federal police, state police, customs, Office of Transport Security, Emergency Management Australia, National Security & International Policy Group for the PM&C, ASIO, and DIO; *n* = 20	Various partnerships are reflected on—joint counter‐terrorism teams (state/feds/ASIO), National Threat Assessment Centre (fusion centre with “several agencies” involved (p. 149), Australia & NZ National Counter‐Terrorism Committee—security, police, emergency response agencies	Agencies/organisations: federal police, state police, customs, Office of Transport Security, Emergency Management Australia, National Security & International Policy Group for the PM&C, ASIO, and DIO	1	1	1	0
Whitehurst (2011)	Thesis	Qualitative semistructured interviews	Police chiefs and sheriffs (*n* = 15)	Information sharing between agencies	Agencies/organisations: federal law enforcement bodies	0	0	2	0
Williams et al. (2016)	Report	Quantitative survey	Programme participants (*n* = 133) and control community members (*n* = 58)	World Organization for Resource Development and Education (WORDE)	Mixed (community and organisations): scholars and community leaders who worked with experts in policy, academia, development and theology. Social services (e.g., social workers, psychologists) and interfaith groups.	1	1	3	0
Wurmb et al. (2018)	Journal article	Qualitative observations	Expert panel (including police, fire, health, emergency services, government, *n* = 14, p. 2)	Expert panel for developing a terrorism response plan/indicators	Agencies/organisations: emergency medical services, fire brigade, government, emergency pastoral care	0	0	3	0
Zecca (2015)	Thesis	Qualitative open‐ended survey responses	Police officers and Muslim volunteers (*n* = 131)	Executive Community Outreach Program	Community: Islamic Society of New Jersey	1	0	1	0

Of the 26 studies eligible for the synthesis, one reached a rating of 3 on the mechanisms domain, one reached a rating of 3 on the moderators domain, 21 reached a 3 on the implementation domain, and four reached a 3 on the economics domain. Table 4 provides an overview of the following details for each of the 26 studies included in the MMIE synthesis: document type, intervention location, year intervention was implemented, funding source, and MMIE domain. The included studies encompass a variety of publication types, including peer‐reviewed journal articles (*n* = 8), dissertations (*n* = 6) and technical research reports (*n* = 12), and span 2001–2018. The interventions were implemented predominantly in the United States (*n* = 17), but covered other countries including: Australia (*n* = 2), Canada (*n* = 2), UK (*n* = 2), Germany (*n* = 1), Kenya (*n* = 1) and multiple international locations (*n* = 1). A total of 16 interventions and/or evaluations were funded, predominantly by US government departments such as the Department of Justice and DHS (*n* = 12), with other funding sources including research fellowships and grants (*n* = 4).

**Table 4:** Overview of studies included in MMIE synthesis (Document type, location, year and funding)

Study	Document type	Location	Intervention year	Funding	MMIE domains
Carter et al. (2014)	Peer‐reviewed journal article	United States (41 unspecified states)	Not reported	Project funded by National Institute of Justice, US Department of Justice (Grant No.: 2008‐IJ‐CX‐0007)	Mechanisms
Burruss et al. (2012)	Government report, technical report, or working paper	United States (states not specified)	Survey conducted 2011	Study/evaluation is funded by a National Institute of Justice, Office of Justice Programs, US Department of Justice grant (Grant No.: 2010‐IJ‐CX‐0024).	Moderators
Van den Heuval et al. (2012)	Peer‐reviewed journal article	UK (no further specification)	Not reported	Not reported	Implementation
Sandoval (2013)	Dissertation	United States (states not specified)	Survey conducted 2010–2011	Not reported	Implementation
Lamb (2013)	Peer‐reviewed journal article	UK (West Midlands)	2011	Not reported	Implementation
Lamb (2012)	Dissertation	Canada (Toronto)	2010	Not reported	Implementation
Lewandowski (2012)	Dissertation	United States (New Jersey)	2007	Not reported	Implementation
Knight (2009)	Dissertation	United States (Tampa Bay, Florida)	2006	Not reported	Implementation
Australian National Audit Office (2010)	Government report, technical report, or working paper	Australia	2009 to present	Not reported	Implementation
Carter (2006)	Dissertation	United States (country wide)	Not reported	Not reported	Implementation
Mabrey et al. (2006)	Peer‐reviewed journal article	United States (Texas)	2003–2005	Texas State University System received funding from the National Institute of Justice ($993,000) to implement the evidence‐based counter‐terrorism and anti‐gang training project	Implementation
Mesloh et al. (2003)	Trade journal article	United States (Orlando, Florida)	2001	Not reported	Implementation
Wurmb et al. (2018)	Peer‐reviewed journal article	Germany (Wuerzburg)	2016	Publication funded by German Research Foundation and University of Wuerzburg	Implementation
Onyango (2018)	Dissertation	Kenya	2015	Research partly funded by Dr James Fyfe Fellowship	Implementation
Braziel et al. (2015)	Government report, technical report, or working paper	United States (California)	2015	Report funded by Office of Community Oriented Policing Services (US Department of Justice, Grant No.: 2015‐CK‐WX‐K005)	Implementation
Department of Homeland Security (2015)	Government report, technical report, or working paper	United States (country wide)	2014	Not reported	Implementation
Department of Homeland Security (2016)	Government report, technical report, or working paper	United States (country wide)	2015	Funding received from federal, state, local, territorial and tribal and private entities (Federal includes DHS and Department of Justice, others not specified)	Implementation
Weine et al. (2017)	Government report, technical report, or working paper	United States (Los Angeles, California)	Not reported	Research supported by DHS Science and Technology Directorate's Office of University Programs through (Award No: 2012‐ST‐061‐CS0001), Centre for the Study of Terrorism and Behaviour (CSTAB) made to START	Implementation
Weine and Braniff (2015)	Government report, technical report, or working paper	United States (Los Angeles, California)	2008	Intervention designed, funded, and implemented by the Los Angeles Police Department with support from DHS Science and Technology Directorate's Office of University Programs	Implementation
Kerry (2007)	Government report, technical report, or working paper	Canada	2002–2007	Federal Government of Canada funded intervention ($59 million), funding for evaluation not specified	Implementation
Williams et al. (2016)	Government report, technical report, or working paper	United States (Maryland)	Unclear	Evaluation and intervention funded by National Institute of Justice, Office of Justice Programs, US Department of Justice	Implementation
Schanzer et al. (2016)	Government report, technical report, or working paper	United States (country‐wide)	Survey conducted 2014	US National Institute of Justice	Implementation
Davis et al. (2016)	Government report, technical report, or working paper	United States (Nationwide intervention; sample from AL, MA, PA, IL, MO)	Ongoing; data from 2014/2015	Bureau of Justice Administration funds and manages the SLATT program	Implementation, Economics
Sandler et al. (2011)	Peer‐reviewed journal article	Multiple (international)	2006–2007	Partly funded by Centre for Risk and Economic Analysis of Terrorism Events (CREATE) at University of Southern California (Grant No.: 2007‐ST‐061–000001), which is supported by the US Department of Homeland Security (DHS)	Economics
Stewart and Mueller (2014)	Peer‐reviewed journal article	Australia (10 largest city airports)	2010/2011 data for costs	Evaluation likely funded by Australian Research Council (see acknowledgements)	Economics
Stewart and Mueller (2018)	Peer‐reviewed journal article	United States (country‐wide)	2016 data for cost estimates	Australian Research Council (Grant No.: DP160100855)	Economics

The 26 studies covered a wide range of intervention approaches (Table 5), including:

**Table 5:** Overview of studies included in MMIE synthesis (Research design, partners, intervention and participants)

Study	Research design and EMMIE domain	Partners	Intervention	Participants
Carter et al. (2014)	Quasi‐experiment (cross‐sectional)	Not clearly specified, however the dependent variables measure a scale of level of involvement with other agencies, as listed: FBI, other federal LEAs, state LEAs, local LEAs, the state Fusion Centre, another state's Fusion Centre	Degree to which agency aligned with the Department of Homeland Security Target Capabilities List (TCL). The TCL provides a guide for LEAs which emphasises intelligence and information sharing to assist agencies prevent/respond to/recover from “major events” (including terrorism)	Practitioners: Law enforcement personnel from state, local and tribal LEAs (specifically, personnel considered to be representative of intelligence functions within their respective agencies, *n* = 272)
	Mechanisms			
Burruss et al. (2012)	Quasi‐experiment (cross‐sectional)	No explicit entity, but reference to partners	Respondent's perceived level of influence that grants from four partners (DHS, private industry, community, or corporate bodies) had on their agency's current practices regarding homeland security prevention, preparedness, response and recovery	Practitioners: Chief executive (or other representative) from state and local law enforcement agencies (*n* = 350)
	Moderators			
Australian National Audit Office (2010)	Audit	Attorney‐General's Department (manages operation of NSH), Australian Security Intelligence Organisation (ASIO), Australian Federal Police (AFP), and state/territory police agencies	National Security Hotline (NSH) 27‐station call centre in the ACT with ~100 operators and supervisors (as of 2010), operating 24 h a day, 365 days per year. Designed to take calls from the public regarding potential terrorism activity and pass this information on to stakeholder agencies (AFP/ASIO/state and territory police agencies). The call centre receives an average of 50 calls per day	Practitioners: Attorney‐General's Department, AFP, ASIO staff. Organisations: Documents from the three organisations, observations of call centre operations, examination of NSH database
	Implementation			
Braziel et al. (2015)	Case study	Multiple police agencies, community stakeholders (i.e., church leaders and employees), and counsellors	San Bernardino public safety response to the terrorist shooting incident at the Inland Regional Centre (December 2, 2015). Response time total: 10.5 h	Practitioners and citizens involved in/impacted by the San Bernardino shooting (e.g., first responders, public safety executives, SWAT team members, victims and their families, community leaders, and faith leaders impacted by or involved in the San Bernardino shooting)
	Implementation		
Carter (2006)	Case study	Development and delivery of programme: School of Criminal Justice at Michigan State University in partnership with the Intelligence Directorate of the Federal Bureau of Investigation and Intelligence Division of the Drug Enforcement Administration	*Intelligence Toolbox* training programme. 2 days, 20–24 h. Face‐to‐face, seven modules: overview of law enforcement intelligence, community partnerships, intelligence products and resources, developing a mission, intelligence capacity building, intelligence led policing, and external funding	Practitioners: State, local and tribal law enforcement officers attending training (*n* = 175)
	Implementation			
Davis et al. (2016)	Case study and cost‐benefit analysis	Development and delivery of programme: Institute for Intergovernmental Research. Workshops often include a panel of local representatives from FBI, Joint Terrorism Task Forces, and other government agencies to facilitate networking	*State and Local Anti‐Terrorism Training* (SLATT) programme. Typically 2.5 days for investigative workshops and typically 1.5 days for train‐the‐trainer workshops. Face‐to‐face, workshop content is tailored to each agency but often focuses on investigative or intelligence techniques	Case study—Practitioners: Institute for Intergovernmental Research staff (*n* = 11). Cost‐benefit analysis—Practitioners: Law enforcement officers (*n* = 124)
	Implementation and Economics			
Department of Homeland Security (2015)	Process evaluation	Fusion Centre implementers and partners (e.g., law enforcement, FBI, Federal Emergency Management Agency)	Fusion Centres (national examination of all centres).	Organisations: Fusion Centres (*n* = 78), federal agencies that provide resources to Fusion Centres (*n* = 43). Practitioners: Fusion Centre customers (e.g., state Homeland Security Advisors, police chiefs, sheriffs, and FBI representatives, *n* = 199)
	Implementation			
Department of Homeland Security (2016)	Process evaluation	Fusion Centre implementers and partners (e.g., law enforcement, FBI, Federal Emergency Management Agency)	Fusion Centres (national examination of all centres)	Organisations: Fusion Centres (*n* = 77), federal agencies that provide resources to Fusion Centres (*n* = 43). Practitioners: Fusion Centre customers (e.g., state Homeland Security Advisors, police chiefs, sheriffs, and FBI representatives, *n* = 158)
	Implementation			
Kerry (2007)	Case study	Canadian Emergency Management College of Public Safety Canada, the Public Health Agency of Canada, Health Canada, the Royal Canadian Mounted Police, Defence Research & Development Canada, and the Canadian Nuclear Safety Commission	Chemical, Biological, Radiological, & Nuclear (CBRN) First Responder Training Program. Involves introductory/awareness, intermediate and advanced, and health‐specific courses for first responders and receivers	Practitioners: key stakeholders, first responders from the development team, departmental managers, Canadian Emergency Management College staff/instructors/students. Organisations: Document review
	Implementation			
Knight (2009)	Case study	FBI, Immigration and Customs Enforcement, Customs and Border Protections, United States Secret Service, United States Marshals Service, United States Coast Guard, Department of Defense, Tampa International Airport	Fusion Centre: Tampa Fusion Centre. Includes Joint Terrorism Task Force and Regional Domestic Security Task Force as major components	Practitioners: law enforcement professionals (*n* = 23). Organisations: Observations of fusion centre collaborative meetings (*n* = 12)
	Implementation			
Lamb (2012)	Case study	Royal Canadian Mounted Police, Ontario Provincial Police, Toronto Police Service, Peel Regional Police Service, Canadian Security Intelligence Service, Communications Security Establishment Canada, Canada Border Services Agency, and Canadian Forces	G20 Joint Intelligence Group—multiagency network to collect data and knowledge on individuals with the potential to disrupt the G20 in Toronto, Canada in 2010. Liaison management team worked with external partners regarding terrorist threats	Organisations: documentation (practitioner documents from the Royal Canadian Mounted Police and Ontario police departments, *n* = 14)
	Implementation			
Lamb (2013)	Case study	Community members, community stakeholders, a local religious organisation (not further specified) and their associated youth group	“Three cups of tea” police‐community partnership within the Prevent stream of the CONTEST policy. Face‐to‐face interactions structured around three stages: familiarisation, trust‐building, and activities to broach the subject of terrorism	Practitioners: security and partnership police officers within Counter Terrorism Unit (*n* = 16)
	Implementation			
Lewandowski (2012)	Case study	FBI, DHS, Alcohol, Tobacco & Firearms, Immigration & Customs Enforcement/Homeland Security Interventions, National Crime Information Centre, Customs and Border Patrol, Secret Service, Diplomatic Security Service, New Jersey State Police, New Jersey Office Of Homeland Security & Preparedness, New Jersey Department Of Corrections, New Jersey Probation & Parole, New Jersey Fire Service, New Jersey Treasury, Essex County Prosecutor's Office, Newark Police Department, Paterson Police Department, Camden Police Department, Ocean County Prosecutor's Office	Fusion Centre: New Jersey Regional Operations Intelligence Centre (ROIC). The threat watch desk, which falls under the threat analysis arm, is where most of the collaboration occurs. Involves preparation of reports and assessments when incidents occur or are planned and may involve homeland security issues and/or terrorism	Practitioners: Employees of the ROIC (*n* = 12). Organisations: Observations of daily operations (over 18‐month period)
	Implementation			
Mabrey et al. (2006)	Case study	Sam Houston State University (lead institution) coordinating the efforts of Texas State University at San Marcos, Lamar University, Sul Ross University, and Angelo State University	Development and implementation of training for law enforcement officers at Mexican border. 5 modules over 20 h and 187‐page manual. Content was specific to issues around terrorism and organised crime at the Texas/Mexico border. Nonintrusive interviewing model to detect false documentation	Practitioners: Implementers and training participants (*n* = 300)
	Implementation			
Mesloh et al. (2003)	Implementation	The University of Central Florida	Amnesty boxes. Sealed container that allows persons to discard contraband items within it before entering a metal detector or security checkpoint. Accompanying interventions included narcotics detector dog 4 h before the concert and signage around the exterior of the auditorium explaining the purpose of the amnesty boxes. Uniformed and plainclothes police were deployed in the area during the event to monitor patron behaviour	Practitioners: University staff, PhD students, and University of Central Florida Police Department police officer (*n* = 3)
Onyango (2018)	Process evaluation	National Counter‐Terrorism Centre of Kenya (multiagency group including state agencies, collaborates with nonstate agencies)	Terrorism Amnesty Reintegration Program (ARP). Amnesty to radicalised Kenyan youths who voluntarily surrender to Kenyan national security agencies. Involves training, counselling, access to social services, reintegration kits to aid economic independence, and monitoring by authorities	Practitioners: National Counter‐Terrorism Centre security personnel and Kenyan Police Service Gazetted Officers (*n* = 4). Documents: Content analysis of newspapers and government documents (*n* = 150)
	Implementation			
Sandoval (2013)	Raw unadjusted correlational design	Military, homeland security, intelligence/information, economic, and diplomatic personnel	Perceived information sharing between practitioners on policy, legal, cultural, technology and trust domains	Practitioners: Individuals from “communities of interest” including military, law enforcement, homeland security, intelligence/information, economic and diplomatic (*n* = 180)
	Implementation			
Schanzer et al. (2016)	Case study	Muslim groups, agencies including churches and social services	Police‐community partnerships and outreach. Interview and focus group data examine facilitators and barriers to a number of different initiatives (e.g., forums, meetings, training content), with participants reflecting on what is implemented locally in their jurisdiction	Practitioners: Municipal and county law enforcement officers (*n* = 19 telephone interviews and *n* = 50 interviews during site visits) Citizens: Muslim American communities (*n* = 200)
	Implementation			
van den Heuvel et al. (2012)	Process evaluation	Home Office, Security Service, and Ministry of Defence	Situation Assessment, Formulate a plan, Execute a plan, and Team learning (SAFE‐T) model for team decision making in counterterrorism operations. Comprises three iterative decision phases: situation assessment, plan formulation, and plan execution, followed by team learning	Practitioners: Senior police officers, and representatives from the Home Office, Security Service, and Ministry of Defence (*n* = 136)
	Implementation			
Weine and Younis (2015)	Process evaluation	Government stakeholders, public, private and faith‐based organisations, nongovernment organisations and local communities	The Liaison Section of the Las Angeles Police Department (LAPD) Counter‐Terrorism and Special Operation Bureau	Practitioners (LAPD officers) citizens and community leaders/advocates (*n* = = 70). Organisations: Field observations of policing activities (*n* = 40 h)
	Implementation			
Weine et al. (2017)	Process evaluation	Healthcare organisations, faith‐based organisations, government, nongovernment organisations, and academic institutions (i.e., schools and universities)	Practices of countering violent extremism‐tailored community policing. Observation and interview data examine a number of different initiatives more generally (e.g., community forums, interfaith events, community engagement, education and training)	Practitioners: Community police officers working with Muslim communities (*n* = 15). Citizens: Muslim American community members and community advocates (*n* = 90). Organisations: Field observations of policing activities (*n* = 100 h)
	Implementation			
Williams et al. (2016)	Quasi‐experiment (matched control group without baseline measures)	World Organization for Resource Development and Education (WORDE) group comprised of scholars and community leaders who worked with experts in policy, academia, development and theology. Social services (e.g., social workers, psychologists) and interfaith groups	World Organization for Resource Development and Education (WORDE) program. Three interlocking countering violent extremism programs: town hall meetings to educate community members, capacity‐building training for law enforcement and social services networks, and volunteerism and multicultural activities for community	Citizens: Current WORDE participants (*n* = 133) and prospective participants (*n* = 58)
	Implementation			
Wurmb et al. (2018)	Case study Implementation	Medical rescue control centre and emergency medical services	Multiagency response to a terror attack in Wuerzburg, Germany (July 18, 2016) Response time total: 106 min	Organisations: Police and medical rescue services (official data)
	Implementation			
Stewart and Mueller (2018)	Cost‐benefit analysis	Federal Air Marshals, transport security inspectors, behaviour detection officers, explosive specialists, and airport officials	Joint Terrorism Task Forces (JTTF) and Visible Intermodal Protection Response (VIPR) teams	Other: Security measures used at US airports (*n* = 18)
	Economics			
Stewart and Mueller (2014)	Cost‐benefit analysis	Australian Customs and Border Protection Service	Australian Federal Police airport counterterrorism policing. Two specific teams: Joint Airport Investigation Teams (to target serious/organised crime in the aviation network) and Joint Airport Intelligence Groups (coexist with the former teams to provide dedicated intelligence support to the unified policing model)	Organisations: Australian Federal Police counterterrorism policing unit
	Economics			
Sandler et al. (2011)	Cost‐benefit analysis	Interpol General Assembly of delegates from member countries, Interpol Executive Committee (elected by General Assembly), member countries' national central bureaus (NCBs), the United Nations, and “all organisations, authorities, and services whose mission is to prevent or combat international crime” (p. 82)	Use of Interpol assets to create the Public Safety and Terrorism subdirectorate for the purpose of coordinating its member countries' counterterrorism efforts. International channels for disseminating information about suspected terrorism	Other: Scenarios/hypothesised events and actual event data
	Economics

- Multiagency information sharing approaches, including fusion centres (*n* = 6);
- Training with either collaborative development and implementation or that contains multiagency working as a component (*n* = 5);
- Community‐police‐agency partnership approaches (*n* = 5);
- Specialised task forces, networks, or localised integration of formalise federal frameworks that emphasise partnership and multiagency working (*n* = 6);
- Mass public safety responses to specific terrorist incidents (*n* = 2);
- Strategies for citizens to partner with agencies by reporting information or reducing risks at public events (*n* = 2) and
- Multiagency reintegration programs for at‐risk youth (*n* = 1)

Most studies did not specify the exact number of partners in addition to police, but for those that did, the number of partners ranged from 1 to 14. Partners, in addition to police, included community organisations (e.g., churches), citizens, and agencies within and outside the criminal justice system at federal and local levels. The vast majority of studies used practitioners as direct participants, with some studies including both practitioners and citizens and only one study using at‐risk individuals or offenders (Williams et al., 2016). The included studies encompass qualitative, quantitative, and mixed‐methods methods such as case‐studies, cost‐benefit analyses, cross‐sectional surveys, interviews and process evaluations.

#### 5.1.3 Excluded studies

Due to the number of full‐text documents screened (*n* = 5149), we are unable to describe the full body of excluded studies. The vast majority of studies were excluded due to absence of a police‐involved multiagency intervention aiming to counter violent extremism (*n* = 2365) or because study authors did not report an empirical study of an eligible intervention (*n* = 2014). Due to the number of excluded studies, the “References to excluded studies” only contains those studies that were deemed to at least reference an eligible intervention, but that did not report of an empirical study of that intervention (*n* = 2014). The “References to excluded studies” also lists the studies that were excluded because they did not meet the research design or outcome measure criteria for the effectiveness component of the review (Objective 1) and also did not report on any data pertaining to mechanisms, moderators, implementation or economic considerations (Objective 2, *n* = 234).

### 5.2 Risk of bias in included studies

#### 5.2.1 Effectiveness studies (Objective 1)

Table 6 summarises the degree of bias for the Williams et al. (2016) study, along with specific reasons for ratings across the seven domains, based on the standardised questions provided by the ROBINS‐I tool. The study was rated as having serious risk of bias for the confounding, selection, classification of interventions, and measurement of outcomes domains. Lack of information or ambiguity in the available study reports and data provided by the authors led to a rating of “no information” for the deviations from intended interventions and the missing data domains. The selection of reported results domain was rated as having a moderate risk of bias. Overall, these ratings suggest that this study has a relatively serious risk of bias.

**Table 6:** Results for risk of bias assessment for Objective 1 (ROBINS‐I) for Williams et al. (2016)

Risk of bias domain	Rating	Rationale
Confounding	Moderate/Serious	No baseline assessment of outcome measures or other potential confounders that might influence engagement with the treatment or comparison condition activities. In addition, it is unclear if authors verified if comparison participants had no interaction with the treatment (WORDE program), which raises the risk of time‐varying confounding because participants may have received both interventions. While the authors state that propensity score matching was used, their published report and data provided by the authors for the purposes of this review do not allow for independent assessment of the propensity score matching. Specifically, the variables the authors use to “match” participants vary within the report are not clear. For example, one section states participants were matched on demographics and another section states the participants were matched on nine measures including: trust in police, political extremism, racism, resiliency and coping, emotional stability, amped political extremism, historical loss, religiosity, and religious dogmatism. The time of measurement for these potential confounders is also not clear (e.g., whether at the beginning and end of the intervention)
Selection	Serious	Participants were invited to participate in the evaluation after they had already begun engagement with the WORDE program (treatment) and the treatment group was based on a stratified random sample from those who expressed interest in the study. One of the variables influencing selection included the frequency of attendance. Although authors sampled across regular, infrequent and ‘one‐timer’ attendees, it is unclear how attendance might impact estimates of treatment effect
Classification of interventions	Serious	Intervention and comparison group not clearly defined. Specifically, it is unclear whether authors verified whether comparison group participants had any contact or engagement with the WORDE program. The specific treatment received by comparison group participants is not clearly articulated aside from other multicultural events or volunteerism activities. It is also unclear whether the information used to define the groups was recorded at the start of the intervention or after the intervention and evaluation was underway
Deviations from intended interventions	No information	The intended intervention and actual implementation of intervention were reported by study authors. The authors outlined the aims and activities of WORDE program, but do not discuss implementation challenges or changes made to implementation. The reported variation in attendance for WORDE participants is suggestive of adherence issues, but this is not unexpected in community interventions and the content of the intervention (countering violent extremism)
Missing data	No information	The published report of the study does not provide participant demographics. Data supplied by study authors suggests there was no attrition, but this could be not be independently verified
Measurement of outcomes	Serious	Outcome measurement appears to be equivalent across groups. However, study authors developed the outcome measures based on interviews with WORDE participants (treatment group) and it is not clear whether the same participants are used for the evaluation and design of the instrument. In addition, the way participants answered the survey questions may have been influenced by their knowledge of and self‐selection into the treatment and evaluation
Selection of reported result	Moderate	No prospectively published protocol for the study exists. The published report for the study does not provide the results of the between group analyses, but rather reports data for the treatment group and a statement that there were no statistically significant differences between the treatment and comparison group. Given this statement about lack of differences, it is unlikely that the reported effects or data provided by study authors for this review are selected on the basis of the results from multiple outcome measurements within the one domain, multiple analyses of the intervention‐outcome relationship, or different subgroups. Data was provided by authors upon request

Table 7 summarises the degree of bias for the other four studies included in the synthesis for Objective 1 (Baldwin, 2010; Burruss et al., 2012; Carter et al., 2014; Stewart & Oliver, 2014), which were rated using the EPHPP Quality Assessment Tool. Overall, all studies were rated as having high risk of bias because they received ratings of “weak” on two or more domains. All studies were rated as having selection bias because they either were not representative of the target population, used restricted purposive or self‐selection methods, and/or had response rates <60%. According to the EPHPP tool, all four studies could be rated as “moderate” because they fit the definition of a cohort analytic design provided by the developers. All but one study was rated as weak for confounding factors because they did not examine differences between treatment and comparison groups based on confounding factors, did not specify or measure confounding factors, and/or did not account for them sufficiently in their analyses. Burruss et al. (2012) was rated as “moderate” on this domain, as the authors thoroughly discussed and measured multiple confounding factors and accounted for them in their statistical models. Given the nature of the studies and the difficulty of double‐blinding in applied criminological evaluations, it is unsurprising that all were rated as “weak” for the blinding domain. Although the EPHPP tool specifies that a study can be rated as “moderate” of the study authors do not mention blinding, we believe that a rating of “weak” is more suitable for the four studies because it is likely authors were aware of the intervention allocation during analysis and because respondents may be aware of the nature of the research questions due to the provision of information to secure informed consent. All but one study were rated as “weak” for their data collection methods, as there was variable information about outcome measures in terms of reliability and validity, and in some cases how the outcome variables were operationalised and measured. Burruss et al. (2012) was rated as “moderate” on this domain because the authors provided reliability data for the eligible outcome measure. Another notable limitation across the four studies was the paucity of data regarding recruitment, attrition, sociodemographic characteristics, and treatment of participants in treatment and comparison conditions.

**Table 7:** Summary results for risk of bias assessment for Objective 1 (EPHPP Quality Assessment Tool)

Study	Selection bias	Study design	Confounders	Blinding	Data collection methods
Baldwin (2010)	Weak	Moderate	Weak	Weak	Weak
Carter et al. (2014)	Weak	Moderate	Weak	Weak	Weak
Burruss et al. (2012)	Weak	Moderate	Moderate	Weak	Moderate
Stewart and Oliver (2014)	Weak	Moderate	Weak	Weak	Weak

#### 5.2.2 Mechanisms, moderators, economic, and implementation studies (Objective 2)

Three of the 26 studies included in the assessment of mechanisms, moderators, implementation factors and economic considerations (Objective 2) were also included in the effectiveness component of the review (Objective 1) and their risk of bias assessment can be found in Table 7. Of the remaining 23 studies included in the mechanisms, moderators, implementation and economics component, four were assessed using the CASP Economic Evaluation checklist (Davis et al., 2016; Sandler et al., 2011; Stewart & Mueller, 2018), four were assessed using the CASP Cohort Study checklist (Carter, 2006; Department of Homeland Security, 2016; Sandoval, 2013; van den Heuvel et al., 2012), and 16 studies were assessed using the CASP Qualitative Study checklist (Australian National Audit Office, 2010; Braziel et al., 2015; Davis et al., 2016; Department of Homeland Security, 2015; Lamb, 2012, 2013; Lewandowski, 2012; Kerry, 2007; Knight, 2009; Mabrey et al. 2006; Mesloh et al., 2003; Onyango, 2018; Schanzer et al., 2016; Weine & Younis, 2014; Weine et al., 2017; Wurmb et al., 2018). One study was assessed on both the Economic Evaluation and Qualitative Study checklists because it was included in the synthesis for economic and implementation considerations (Davis et al., 2016). The tools collectively aim to critically appraise studies to inform the degree of confidence in study findings and, overall, the studies varied in their consideration and reporting across the domains, with most domains rated as unclear or high‐risk of lowering confidence in the study findings (see Supporting Information Appendix D for guiding questions informing ratings in each domain).

Table 8 provides the summary of ratings on the CASP Economic Evaluation checklist (see Supporting Information Appendix D for guiding questions informing ratings in each domain). The four economic studies all had well defined research questions or aims yet varied in their ratings across the remaining domains. All but one study (Stewart & Mueller, 2014) included comprehensive descriptions of competing alternatives in their cost‐benefit analyses, yet only one study unequivocally provided evidence for effectiveness for the intervention eligible for this review (Sandler et al., 2011). All but one study (Davis et al., 2016) measured and valued the eligible intervention appropriately. The studies were rated as “No” or “Can't Tell” on the item assessing the identification and measurement of costs and the item assessing whether adequate sensitivity analyses were performed. Only one study (Sandler et al., 2011) adjusted costs and consequences for different times, only two studies (Sandler et al., 2011; Stewart & Mueller, 2014) provided an incremental analysis of consequences and costs for the alternatives provided.

**Table 8:** Summary results for risk of bias assessment for Objective 2 (CASP Economic Checklist, *n* = 4)

Study	Well defined question?	Comprehensive description of competing alternatives?	Evidence for effectiveness?	Effects of intervention measured and valued appropriately?	All important and relevant resources required, and outcome costs for each alternative identified, measured in appropriate units and valued credibly?	Costs and consequences adjusted for different times (discounting)?	Incremental analysis of consequences and cost of alternatives provided?	Adequate sensitivity analysis performed?
Sandler et al. (2011)	Yes	Yes	Yes	Yes	Can't Tell	Yes	Yes	Can't Tell
Stewart and Mueller (2014)	Yes	Can't Tell	Can't Tell	Yes	Can't Tell	No	Yes	No
Stewart and Mueller (2018)	Yes	Yes	Can't Tell	Yes	No	No	Can't Tell	No
Davis et al. (2016)	Yes	Yes	Can't Tell	Can't Tell	No	No	Can't Tell	Can't Tell

Table 9 provides the summary of ratings on the CASP Cohort Study checklist (see Supporting Information Appendix D for guiding questions informing ratings in each domain). The four cohort studies all had well defined research questions or aims yet varied in their ratings across the remaining domains. Two studies utilised appropriate recruitment strategies (Department of Homeland Security, 2016; Sandoval, 2013) and reported complete and appropriate lengths of follow‐up (Department of Homeland Security, 2016; Sandoval, 2013). The studies were rated as “Can't Tell” on the item assessing the whether participant exposure was measured in a way to minimise bias, and two studies measured the outcome in a way to minimise bias (Department of Homeland Security, 2016; Sandoval, 2013). While one study identified important confounding factors (Carter, 2006), the authors did not account for these in their design and analysis, and the remaining three studies were rated as “No” or “Can't Tell” for the identification of confounding factors and accounting for confounders in their design or analysis (Department of Homeland Security, 2016; Sandoval, 2013; van den Heuvel et al., 2012).

**Table 9:** Summary results for risk of bias assessment for Objective 2 (CASP Cohort Study Checklist, *n* = 4)

Study	Well defined question?	Appropriate recruitment?	Exposure measured to minimise bias?	Outcome measured to minimise bias?	Identification of and account important confounding factors in design or analysis?	Complete and appropriate length of follow‐up?
van den Heuvel et al. (2012)	Yes	Can't Tell	Can't Tell	Can't Tell	Can't Tell/No	Can't Tell
Carter (2006)	Yes	Can't Tell	Can't Tell	Can't Tell	Yes/No	Can't Tell/No
Sandoval (2013)	Yes	Yes	Can't Tell	Yes	Can't Tell	Yes
Department of Homeland Security (2016)	Yes	Yes	Can't Tell	Yes	Can't Tell/No	Yes

Table 10 provides the summary of ratings on the CASP Qualitative Study checklist (see Supporting Information Appendix D for guiding questions informing ratings in each domain). Of the 16 studies rated, 14 were assessed to have well defined research questions or aims (Australian National Audit Office, 2010; Braziel et al., 2015; Davis et al., 2016; Department of Homeland Security, 2015; Lamb, 2013; Lewandowski, 2012; Kerry, 2007; Knight, 2009; Mabrey et al., 2006; Onyango, 2018; Schanzer et al., 2016; Weine & Younis, 2014; Weine et al., 2017; Wurmb et al., 2018) and utilisation of a qualitative approach was deemed appropriate for all 16 studies. However, only five of the 16 studies implemented a design appropriate to the research aims (Department of Homeland Security, 2015; Lamb, 2013; Onyango, 2018; Weine & Younis, 2014; Wurmb et al., 2018), while the rest were rated as “Can't Tell” on this item. Only three studies utilised appropriate recruitment strategies (Department of Homeland Security, 2015; Kerry, 2007; Wurmb et al., 2018) and two studies used data collection methodologies appropriate for the research aims or questions (Knight, 2009; Wurmb et al., 2018). One study adequately considered the relationship between the research and participants (Lamb, 2012) and only two studies took ethical considerations into account (Knight, 2009; Wurmb et al., 2018). One study was considered to conduct a rigorous data analysis (Onyango, 2018), yet seven studies were rated as providing a clear statement of study findings (Australian National Audit Office, 2010; Braziel et al., 2015; Department of Homeland Security, 2015; Kerry, 2007; Knight, 2009; Weine & Younis, 2014; Wurmb et al., 2018).

**Table 10:** Summary results for risk of bias assessment for Objective 2 (CASP Qualitative Study Checklist, *n* = 16)

Study	Well defined question?	Qualitative method appropriate?	Research design appropriate for research aims?	Appropriate recruitment strategy?	Data collection appropriate for research?	Relationship between researcher and participants adequately considered?	Ethical issues taken into consideration?	Rigorous data analysis?	Clear statement of findings?
Davis et al. (2016)	Yes	Yes	Can't Tell	No	No	No	No	Can't Tell	Can't Tell
Lamb (2013)	Yes	Yes	Yes	Can't Tell	Can't Tell	No	No	No	Can't Tell
Lamb (2012)	Can't Tell	Yes	Can't Tell	Can't Tell	Can't Tell	Yes	Can't Tell	Can't Tell	Can't Tell
Lewandowski (2012)	Yes	Yes	Can't Tell	Can't Tell	Can't Tell	Can't Tell	No	No	Can't Tell
Knight (2009)	Yes	Yes	Can't Tell	Can't Tell	Yes	Can't Tell	Yes	Can't Tell	Yes
Australian National Audit Office (2010)	Yes	Yes	Can't Tell	Can't Tell	Can't Tell	No	No	Can't Tell	Yes
Mabrey et al. (2006)	Yes	Yes	Can't Tell	Can't Tell	Can't Tell	No	Can't Tell	No	Can't Tell
Mesloh et al. (2003)	Can't Tell	Yes	Can't Tell	Can't Tell	Can't Tell	No	No	Can't Tell	Can't Tell
Wurmb et al. (2018)	Yes	Yes	Yes	Yes	Yes	No	Yes	Can't Tell	Yes
Onyango (2018)	Yes	Yes	Yes	No	Can't Tell	Can't Tell	Can't Tell	Yes	Can't Tell
Braziel et al. (2015)	Yes	Yes	Can't Tell	Can't Tell	Can't Tell	No	No	Can't Tell	Yes
Department of Homeland Security (2015)	Yes	Yes	Yes	Yes	Can't Tell	Can't Tell	No	Can't Tell	Yes
Weine et al. (2017)	Yes	Yes	Can't Tell	Can't Tell	No	No	Can't Tell	Can't Tell	Can't Tell
Weine and Younis (2015)	Yes	Yes	Yes	Can't Tell	Can't Tell	Can't Tell	Can't Tell	Can't Tell	Yes
Kerry (2007)	Yes	Yes	Can't Tell	Yes	Can't Tell	No	No	Can't Tell	Yes
Schanzer et al. (2016)	Yes	Yes	Can't Tell	Can't Tell	Can't Tell	No	No	Can't Tell	Can't Tell

### 5.3 Synthesis of results

#### 5.3.1 Effectiveness studies (Objective 1)

##### Radicalisation to violence outcome category

Williams et al. (2016) provided data that permitted the comparison between intervention participants who received a police‐involved multiagency intervention versus those who did not using a measure of vulnerability to radicalisation. Table 11.1 includes the SMDs and their associated 95% Confidence Intervals for each eligible self‐report survey item from the Williams et al. (2016) study. *RevMan* was used to calculate effect sizes using data supplied by the study authors (propensity‐matched means, standard deviations, and number of participants in each group). The effect sizes were small to medium and favoured the treatment group, aside from one item that favoured the comparison group (survey item: “I make friends with people from other races”). All but three of the SMDs have confidence intervals including zero, indicating a lack of statistically significant differences between participants in the treatment and comparison group for most survey items.

**Table 11.1:** Impact of WORDE program on self‐report deradicalisation outcomes

Survey item	Standardised mean difference	95% confidence interval
I feel welcome	0.47	0.15–0.78
I feel part of something bigger than myself	0.11	−0.20–0.42
I feel a sense of teamwork	0.24	−0.07–0.55
I make friendships that are active beyond the event	0.21	−0.10–0.52
I make friends with people from other races	−0.51	−0.82 to −0.19
I feel free of peer pressure	0.88	0.56–1.20
I feel accepted	0.44	0.13–0.75
I wouldn't feel afraid to talk to others	0.44	0.13–0.75
I learn about cultures other than my own	0.25	−0.06–0.56

##### Multiagency collaboration outcome category

Table 11.2 summarises the four studies included in the synthesis of multiagency outcomes. Carter et al. (2014) used three negative binomial regression models to examine the impact of agency alignment with the TCL on the degree of close working relationships with external organisations, frequency of information sharing with external agencies, and frequency of receiving information from external agencies. The authors concluded that greater alignment with the TCL was positively and statistically significantly related to greater working relationships with other organisations, as well as with providing and receiving intelligence from these organisations. The authors present results as exponentiated coefficients, or IRRs. For every one unit increase in a policing agency's alignment with the TCL (from 1 = not at all, to 4 = completely), there is a corresponding 3.6% increase on the scale measuring close working relationships with external organisations (IRR = 1.036, *SE*_IRR_ = 0.012, LCL_IRR_ = 1.013, UCL_IRR_ = 1.060), an 8.1% increase on the scale measuring the extent to which they provide intelligence to external agencies (IRR = 1.081, *SE*_IRR_ = 0.025, LCL_IRR_ = 1.032, UCL_IRR_ = 1.13), and a 6.1% increase on the scale measuring the extent to which they receive intelligence from these partners (IRR = 1.061, *SE*_IRR_ = 0.031, LCL_IRR_ = 1.000, UCL_IRR_ = 1.122).

**Table 11.2:** Summary of studies using multiagency collaboration outcomes

Study	Outcome	Effects
Carter et al. (2014)	Degree of close working relationship with external organisations	IRR = 1.036, *SE*_IRR_ = 0.012, LCL_IRR_ = 1.013, UCL_IRR_ = 1.060
	Frequency of information sharing with external agencies	IRR = 1.081, *SE*_IRR_ = 0.025, LCL_IRR_ = 1.032, UCL_IRR_ = 1.13
	Frequency of receiving information from external agencies	IRR = 1.061, *SE*_IRR_ = 0.031, LCL_IRR_ = 1.000, UCL_IRR_ = 1.122
Baldwin (2010)	Clarity and understanding of federal DHS missions, responsibilities, strategies, and/or goals	*r* = −0.212, *SE*_*r*_ = 0.049, LCL_*r*_ = −0.308, UCL_*r*_ = −0.117
	Clarity and understanding of state‐level missions, responsibilities, strategies, and/or goals	*r* = 0.173, *SE*_*r*_ = 0.041, *LCL*_*r*_ = 0.093, UCL_*r*_ = 0.253
	Clarity and understanding of department‐level missions, responsibilities, strategies, and/or goals	*r* = 0.161, *SE*_*r*_ = 0.053, LCL_*r*_ = 0.058, UCL_*r*_ = 0.264
Burruss et al. (2012)	Homeland security preparedness actions (including multiagency partnership initiatives)	*r* = −0.288, *SE*_*r*_ = 0.087, LCL_*r*_ = −0.458, UCL_*r*_ = −0.118
Stewart and Oliver (2014)	Homeland security initiatives (including multiagency partnership initiatives)	*B* = 0.22, *SE*_*B*_ = 0.08, LCL_*B*_ = 0.063, UCL_*B*_ = 0.377

Abbreviations: DHS, Department of Homeland Security; IRR, incident rate ratios; LCL, lower confidence limit; UCL, upper confidence limit.

Baldwin (2010) conducted three separate linear regression models, assessing the impact of the number of collaborations on the level of understanding of DHS missions, responsibilities, strategies, and/or goals at the federal, state, and departmental level. Each model controlled for a range of other demographic, law enforcement practice, and ideology variables. The effect of the number of collaborations was mixed across outcomes. A higher number of collaborations was associated with a lower rating of clarity and understanding of federal DHS missions, responsibilities, strategies and/or goals (*r* = −0.212, *SE*_*r*_ = 0.049, LCL_*r*_ = −0.308, UCL_*r*_ = −0.117); however, a higher number of collaborations was associated with a higher rating of clarity and understanding of state‐level DHS missions, responsibilities, strategies and/or goals (*r* = 0.173, *SE*_*r*_ = 0.041, LCL_*r*_ = 0.093, UCL_*r*_ = 0.253), and a higher rating of clarity and understanding of departmental‐level DHS missions, responsibilities, strategies and/or goals (*r* = 0.161, *SE*_*r*_ = 0.053, LCL_*r*_ = 0.058, UCL_*r*_ = 0.264).

Burruss et al. (2012) used structural equation modelling (SEM) to examine the impact of perceptions of the influence of grants from DHS, private industry, community, or corporate bodies on homeland security preparedness. The grants variable is defined as the influence of partner grants on formulating the “agency's current approach or practices related to homeland security prevention, preparedness, response, and recovery” (Burruss et al., 2012, p. 108). The first model, which controls for institutional pressures, terrorism risk, nonterrorism risk, and agency size, shows a negative direct relationship between perceptions of the influence of grants and homeland security preparedness (*r* = −0.333, *SE*_*r*_ = 0.0913, LCL_*r*_ = −0.512, UCL_*r*_ = −0.154). Similarly, the full model indicates that when taking into account institutional pressures, perceived terrorism and nonterrorism risk, agency size, rurality, and connections to larger agencies, there was a negative direct relationship between the perception of the influence of grants and the number of homeland security preparedness activities that were conducted (*r* = −0.288, *SE*_*r*_ = 0.087, LCL_*r*_ = −0.458, UCL_*r*_ = −0.118). The authors state that this negative result was not expected and hypothesise that this may be due to a suppression effect in the models. While this hypothesis was not tested, the authors suggest that agencies that are not influenced by institutional pressures but seek grants may engage in fewer homeland security preparedness activities.

Stewart and Oliver (2014) used a zero inflated negative binomial regression model (ZINB) to explore the relationship between receipt of homeland security grants and the number of homeland security initiatives. The authors found that the receipt of homeland security funding did not significantly predict whether or not the agency engaged in *at least one* form of homeland security innovation (*B* = −1.12, *SE*_*B*_ = 0.67, LCL_*B*_ = −2.433, UCL_*B*_ = 0.193). However, for those agencies that had engaged in *at least one* homeland security innovation, the receipt of homeland security funding was associated with an increased number of homeland security initiatives, a measure which includes initiatives that focus on multiagency collaboration (*B* = 0.22, *SE*_*B*_ = 0.08, LCL_*B*_ = 0.063, UCL_*B*_ = 0.377). While this study and Burruss et al. (2012) may appear to use a similar intervention variable related to grants, these studies were not combined using meta‐analysis because both the intervention and the outcomes were considered too conceptually distinct. Burruss et al. (2012) focused on the perceived influence of grants on homeland security preparedness, whereas Stewart and Oliver (2014) focused on whether or not homeland security grants had been received. Similarly, the outcome used in Burruss et al.'s (2012) study was focused on homeland security preparedness, whilst the outcome in Stewart and Oliver's (2014) study was focused on the implementation of homeland security innovations.

### 5.4 Mechanisms, moderators, economic and implementation studies (Objective 2)

#### 5.4.1 Mechanisms

Of the 181 studies deemed eligible for Objective 2 of the review, only one study reached a rating of 3 or more on the EMMIE appraisal tool for examining mechanisms underpinning multiagency interventions with police as a partner for countering radicalisation, extremism and/or terrorism (Carter et al., 2014, see Table 4). This indicates that most studies providing at least some content on potential mechanisms either provided only a general statement of assumed theory or a description of a theory linked with potential mechanisms, but did not provide a full description of the theory of change underpinning the intervention and testable predictions (rating of 3 on EMMIE tool) or conduct a robust analysis to determine if the theory of change operates as expected (rating of 4). Although the authors of the sole study with a rating of 3 do not directly test the proposed mechanism, of all the studies rated on the EMMIE tool, this was the only study that provided an explicit and cohesive description of theory drawn from prior work (rating of 2) along with a description of a theory of change and testable predictions (rating of 3).

Carter et al. (2014) used a survey of 272 law enforcement agencies to categorise a range of practitioners on the degree to which they aligned with the DHS TCL. These independent variables were conceptualised as the multiagency intervention as they explicitly encourage multiagency working in the context of terrorism, extremism and/or radicalisation (see Section 5.1.2). The NCISP is a guide prepared by the Global Intelligence Working Group and U.S. Department of Justice that provides a framework detailing the characteristics that law enforcement entities should adhere to in order to practice successful intelligence‐led policing. The TCL provides a guide for law enforcement agencies that aims to assist agencies in their ability to prevent/respond to/recover from “major events,” including terrorism.

Carter et al. (2014) use loose coupling theory to contextualise their study, which characterises organisations as being split into two internal levels: (1) superordinate level and (2) subordinate level. The superordinate level acts in a way to satisfy the expectations of the external environment and the subordinate level follows the superordinate's prescription for the agency but retains some level of independence in how they perform their duties. The theory “suggests that both groups do not always work in tandem” (p. 434) and refers to potential disconnect between the two which can be influenced by factors such as agency size.

Unfortunately, the authors use the intervention variables (degree of adherence to multiagency frameworks) as proxies for testing the theory rather than measuring and testing whether the superordinate/subordinate level and the cohesive working components discussed in loose coupling theory exist within the organisations or in their relationship with partners. This lack of explicit measurement of the proposed mechanisms prohibited direct testing of the loose coupling theoretical mechanisms (e.g., degree of adherence to frameworks→cohesiveness→outcome).

#### 5.4.2 Moderators

Of the 181 studies deemed eligible for Objective 2 of the review, only one study reached a rating of 3 or more on the EMMIE appraisal tool for examining factors that may moderate the effect of multiagency interventions with police as a partner for countering radicalisation, extremism, and/or terrorism (Burruss et al., 2012, see Table 4). This indicates that most studies reporting at least some content on potential moderators either provided ad hoc descriptions of possible moderators or tested possible post hoc moderators yet did not (a) provide a theoretically grounded description of relevant moderators (rating of 3); or (b) collect and analyse data relating to the theoretically grounded moderators and/or contexts (rating of 4).

Burruss et al. (2012) used data from a self‐report survey to examine the level of national security preparedness among a sample of 350 state and local U.S. law enforcement agencies^8^ by measuring the degree to which they employed 13 actions that denoted steps taken by departments to prevent, respond to, and recover from homeland security incidents. These authors examined whether the relationship between preparedness actions (intervention) and organisational efficiency (outcome) was impacted by a range of factors (moderators). The intervention variable was measured by asking respondents to indicate whether they had employed the 13 counterterrorism strategies within their agency, which included actions indicative of multiagency collaboration (e.g., interagency taskforce participation, mutual aid agreements with law enforcement and other agencies). It should be noted that these measures were combined with other actions that were not considered to be multiagency working. As such, the moderation findings need to be interpreted with caution. The outcome of organisational efficiency was measured by asking respondents to rate their agency on a 14‐item scale regarding their ability to respond, in a multiagency way, to a homeland security event.

The authors draw on organisational theory as an overarching framework—encompassing contingency theory, resource dependency theory and institutional theory—to explain how levels of preparedness for national security incidents are shaped. Contingency theory proposes that preparedness activities are rational actions to threats. For example, the perceived risk of a terrorist attack will lead police agencies to adjust their level of preparedness. However, the organisational structure and degree of preparedness actions will be contingent on external factors (e.g., crime rates), size of the organisation, and the availability of technology. The second theory, resource dependent theory, argues that level of preparedness is influenced by external funding to support the adoption of new policies and practices. Lastly, institutional theory proposes that agencies and individuals outside the organisation who provide the organisation with funding, materials, equipment, clients, and other inputs, can place pressure on an organisation to confirm to certain expectations. Hence, pressure coming from external agencies and bodies, such as other police agencies, can act as an important moderator of national security preparedness on organisational efficiency to deal with a homeland security event. Burruss et al. (2012) measured a range of moderators linked with these theories, including: extent of influence or effective modelling by peers and agencies, perceived pressure from professional associations, perceived influence of relevant publications (government and otherwise), agency size and proximity, and grant programs or funding opportunities.

The results of the SEM undertaken by Burruss et al. (2012) found that self‐reported national security preparedness predicted 53% of the variation in perceived organisational efficacy to respond to a terrorism event. In addition, the impact of self‐reported preparedness actions—including multiagency actions—on organisational efficacy was moderated by institutional pressures (*β* = 0.708), followed by interactions with other law enforcement agencies (*β* = 0.298), availability of state and federal grants (*β* = −0.288), the size of an agency (*β* = 0.166), and if a police agency was in a rural or urban area, with those in rural areas reporting lower levels of preparedness (*β* = −0.157). The authors argue that institutional pressures lead to agencies being more aware of professional practice trends and literature, and that interactions with other agencies and the size of the agency are likely to magnify the influence of these practice and literature trends. Specifically, they contend that connections with “external bodies and individuals (such as training organisations, professional associations, government agencies, scholars, and other law enforcement agencies) can help shape the structures and activities of law enforcement agencies” (p. 66) by increasing diffusion of knowledge and by creating “change agents” who can disseminate information and encourage the adoption of practices and policies to enhance preparedness.

#### 5.4.3 Implementation considerations (facilitators)

Of the 181 studies deemed eligible for Objective 2 of the review, 21 studies reached a rating of 3 or more on the EMMIE appraisal tool for examining implementation considerations regarding multiagency interventions with police as a partner for countering radicalisation, extremism, and/or terrorism (see Table 4). This indicates that most studies reporting at least some content on implementation considerations either provided ad hoc comments or “concerted efforts to document implementation or implementation challenges” (Thornton et al., 2019, p. 271), yet did not provide (a) an “evidence‐based account of levels of implementation or implementation challenges” (p. 271); or (b) “a complete evidence‐based account of implementation or implementation challenges and specification of what would be necessary for replication elsewhere” (p. 271). Table 12 provides an overview of the practical implementation stages and considerations reported in each of the 21 studies. Of the 21 studies, 16 discuss factors that enable implementation of the intervention (i.e., facilitators).

**Table 12:** Overview of implementation considerations

Study	Implementation in Practice
Australian National Audit Office (2010)	Origins: Department of Prime Minister and Cabinet was appointed to oversee the National Security Campaign. This included a public information campaign and consultation with research, leading to the decision to create a dedicated Commonwealth 24/7 response number for people to report information regarding national security (National Security Hotline, NSH)
	Operations, Materials, and Resources:
	The NSH itself is a single organisation that liaises with ASIO, AFP and citizens, but the degree of multiagency working involved in the following components is unclear
	NSH staff training: Staff are trained to take their time on calls to elicit as much information as they can (p. 31), 5‐day initial training (topics include: questioning, info gathering, negotiation, how to use IT systems), mentoring system, call monitoring until competent, self‐paced e‐learning with simulations, review of prior reports. Specific staffing schedule, 30‐min overlap in shifts to allow for handovers, minimum of 2× operators and 1× supervisor. Explicit contingencies and procedures if call centre is compromised or has a surge in calls. IT and telecommunication specifications (e.g., electronic call flagging and communication to relevant agencies via checkboxes). Explicit procedures for stakeholder agencies who receive NSH call details (automatic email to specific email address nominated by agency, secure web‐based portal). Biannual stakeholder forum with 1–2 representatives from each agency to discuss practices and other issues Joint ASIO and AFP team who evaluates and triages calls, AFP officer embedded in ASIO and has full connectivity between the databases. An intelligence officer downloads calls, checks them for information, and then forwards the proforma to AF who check the call information against their own and sends back, the intelligence officer then assesses whether the call is of no interest, a reference call, or a lead. Specific processes for AFP handling of calls that are not triaged through ASIO (pp. 62–63)
Braziel et al. (2015) Wurmb et al. (2018)	Origins: These two studies provide case studies of multiagency responses to public safety events (e.g., active shooters)
	Operations, Materials, and Resources:
	Dispatch calls to first responders regarding violent lone actors (police initially, followed by other agencies), rapid arrival on scene Timely information sharing and coordination of movements between federal, state, local agencies to locate shooters and triage injured victims Specialised law enforcement unites (e.g., SWAT, explosive experts) and tactical command post. Telecommunication technology to avoid nonlaw enforcement listening into scanners (e.g., push‐to‐talk phones). Triage area and swift transport of wounded victims away from scene to hospitals Evacuation of nearby buildings, clear markers to indicate cleared locations Thorough sweeps of initial shooting site prevented the detonation of secondary weapons Community collaboration to assist in the process of transporting and containing witnesses during lengthy interviewing processes (e.g., churches) Community collaboration to provide a location for witnesses to reunite with family, and a site for counselling services to be accessed Public Information Officers included in all command‐level briefings and strategy sessions and informed the media throughout the day. Social media to communicate alerts and information to the public
Carter (2006)	Origins: Program intended to enhance intelligence capacities of state, local and tribal law enforcement (SLTLE) agencies
	Operations, Materials, and Resources:
	20–24 h of training over two days, sufficient space for group activities (e.g., breakout groups) Student handbook containing all information presented during lectures to supplement individual notes Day 1: Session 1 (overview of intelligence history, initiatives, the process and future directions); Session 2 (community partnerships for intelligence); Session 3 (products and resources, including data networks and availability); Sassoon 4 (infrastructure, such as intelligence plans). Day 2: Session 5 (intelligence capacity building including: information management, civil rights and privacy and liability and intelligence records, auditing), Session 6 (intelligence‐led policing); Session 7 (external funding for supplementing regular budgets). Final phase comprised of 2‐h breakout sessions of 12 people discussing issues or intelligence functions of their agencies in order to identify the obstacles for agencies. Evaluation and data collection occurred during this phase examine the effectiveness of this programme to develop/recalibrate intelligence capacity and how agency size affects adequacy of intelligence capacity
Davis et al. (2016)	Origins: Agencies approached the Institute for Intergovernmental Research (IRR), whose planning staff work with the agency to determine their training needs
	Operations, Materials, and Resources:
	SLATT involves four main activities:
	1. The IRR deliver face‐to‐face on‐site training and workshops, including specific training on certain topics (i.e., investigative/intelligence), expert instruction at meetings, and sessions at conferences. 2. Train‐the‐trainer workshops which aim to develop capacity of trainers in law enforcement agencies to upskill their staff. 3. SLATT trainees can access online training webinars and training modules. 4. IRR provide customised technical assistance to law enforcement stakeholders. Details regarding the modality for intervention components not explicit (e.g., only lecture‐based or include interactive activities). However, authors state that, typically, workshops include a panel of local representatives from FBI, Joint Terrorism Task Forces, and other government agencies to facilitate networking. Training covers an overview of the nature and warning signs of terrorism, and then specific/targeted training around tactics for the respective topic covered in the workshop (e.g., the investigative workshop gives training around the investigative interview, while the train‐the‐trainer workshop provides guidance for attendees to implement when developing their own in‐house training). See Tables 1.1 and 1.2 of the report for further detail Investigative workshops are “typically two‐and‐a‐half days” (p. 8) while train‐the‐trainer workshops are “typically one‐and‐a‐half” days (p. 8). The exact duration seems to depend on the individual agency that requests the training. Specific details are provided regarding logistical management of a SLATT event (p. 30), including marketing and information dissemination. Workshops are advertised via flyers which are disseminated via the requesting agency's email lists
Department of Homeland Security (2015)	Origins: Fusion centres are collaborative centres between two or more agencies that provide and share resources, intelligence, expertise and information in order to prevent, detect, or respond to criminal/terrorist activity. This report focuses on the national network of centres in the United States in 2014
	Operations, Materials, and Resources:
	Business/operating hours vary across fusion centres (e.g., 24/7, extended operating hours, or normal business hours, p. 9). Colocation of fusion centres with different agencies (p. 10 Table 2). The highest number of fusion centres co‐locate with state, county or city law enforcement, but there is a range of other partners that they are co‐located with including law enforcement intelligence, emergency operation centres, state homeland security, FBI, and fire services. Staff predominantly state‐level, with a range of roles (see p. 11) with most focusing on analysis. Least engagement with private sector. Some staff who work in fusion centres but are not paid out of the fusion centre's budget (e.g., public health nurses or fire fighters who might be assigned as subject matter experts or analysts) Creation of “standing information needs” (SINs) to tag subjects of operational or intelligence interest. This tracks the overall operations and whether they are meeting customers' needs (see p. 15, which also assesses whether they are tagging SINs accurately/appropriately) DHS Grant Program requires all fusion centres to “post all distributable analytic products on HSIN‐Intel” (database, p. 16‐17). Biweekly threat information sharing forum implemented alongside this Provision of direct support to preplanned events and disasters (p. 18) Governance bodies usually oversee and guide fusion centre budgets, programs and operations (see p. 21 and 22 Distribution of federal funding assists fusion centres predominantly to include personnel, training, support for National Network (i.e., headquarters), IT, and training (see p. 28)
Department of Homeland Security (2016)	Origins: Fusion centres are collaborative centres between two or more agencies that provide and share resources, intelligence, expertise and information in order to prevent, detect, or respond to criminal/terrorist activity. This report focuses on the national network of centres in the United States in 2015
	Operations, Materials, and Resources:
	Fifty‐three operate at state or territorial level (have responsibility of entirety of states or territories), remaining 25 fusion centres operate in major urban areas and have smaller geographic responsibility Business hour variance (p. 5), based on mission requirements and mission resources 89.6% (96) of fusion centres located either in the same office space or building with at least one other federal or SLTT agency Total of 2479 LSTT and private sector staff members and most common role is analyst (p. 6) Operational costs, including personnel, account for the overwhelming majority of all expenditures for centres
Kerry (2007)	Origins: The CBRN First Responder Training Program (FRTP) is multiagency programme coordinated by Public Safety Canada. It aims to increase preparedness, readiness and capability to respond” to terrorist‐related incidents in Canada” (p. iii)
	Operations, Materials, and Resources:
	Funded under public security and terrorism Content focused on introductory/awareness, intermediate and advanced, and health‐specific courses for first responders and receivers with 4 expected outcomes: awareness, basic, intermediate and advanced (see p. 8) Flowchart clearly specifies development, delivery, management, and coordination of the training (p. 9) Roles and responsibilities of each stakeholder organisation is clearly delineated (p. 10) Requires a real‐life scenario as a training simulation, along with a complete team of first responders (i.e., fire, police, emergency medical services, p. 24)
Knight (2009)	Origins: Fusion centres are collaborative centres between two or more agencies that provide and share resources, intelligence, expertise and information in order to prevent, detect, or respond to criminal/terrorist activity. This dissertation focuses on the Tampa fusion centre including findings from direct observations of collaborative sessions/meeting/interviews of fusion centre participants and the federal/state managers and supervisors
	Operations, Materials, and Resources:
	See above detail for DHS (2015) and DHS (2016) Top‐down model with DHS centralised administration operational directions (e.g., federal funding, training, and standardising effective and recommended procedures, see p. 26) A networked model for fusion centres requires modification to obtain the collaborative effort and action desired (e.g., input and participation by individuals, clear roles and responsibilities linked with procedures)
Lamb (2012)	Origins: Joint Intelligence (JIG) multiagency network formed to collect intelligence on individuals who were at‐risk of disrupt the G20 event in Toronto
	Operations, Materials, and Resources:
	Interconnected teams, including: Liaison Management Team, which works with external partners to keep abreast of potential terrorist threats (see p. 78); Domestic Intelligence Liaison Management Team (for liaison with other police/military/security agencies, see p. 79); International Intelligence Liaison Management Team (p. 81); and Corporate Intelligence Liaison Management Team (p. 82) Weekly briefs leading up to the G20 to keep partners abreast of any issues of concern leading up to the event (p. 149) Creation and dissemination of Intelligence Bulletins (p. 149, frequency not specified) Closer to the event, weekly reports replaced with “Daily Situational Reports” (p. 150). These reports contained information specific to likely public safety events (including terrorism) and threat and risk‐level assessments to inform intelligence and planning (p. 152 onwards)
Lamb (2013)	Origins: The “three cups of tea” approach was embedded within the PREVENT stream of the CONTEST policy in the UK
	Operations, Materials, and Resources:
	The “three cups of tea” approach entails a series of interactions between Security and Partnership officers and their assigned community members/area/institution, and is an adaptation upon existing community policing approaches in the area Security and Partnership officers work under the area Counter Terrorism Unit, not the general West Midlands policing units (although can work alongside them), and do not wear a radio nor attend to other calls so that they may fully devote their time to building community relationships. Also, crucially, these relationships are built through face‐to‐face interactions The “first cup of tea” signals the beginning of the relationship (familiarisation) between officer and community partner through continued instigation on the officer's part. In this stage it is imperative that officers disclose that they work for the Counter Terrorism Unit, and are clearly uniformed, to maintain transparency and avoid feelings of betrayal or perceived deception. Their uniforms are also worn to grant visibility in a way that is similar to neighbourhood police, with which community members are already familiar. In order to effectively differentiate themselves from local neighbourhood officers and foster sustained relationships, Security and Partnership officers are required only to spend meaningful time in the community—as such they are not required to wear a radio and complete other policing jobs. Once officers believe a level of familiarity has been established they progress onto the next stage In the “second cup of tea” phase, officers build trust with community partners by capitalising on any opportunity to assist in small community problems, streamlining solutions by sidestepping “red tape”. This stage entails Security and Partnership officers to move beyond establishing familiarity and towards gaining the trust of community so community members feel comfortable in discussing sensitive topics related to terrorism/extremism/radicalisation. This includes assisting and addressing small concerns and issues of the community to demonstrate officers' genuine willingness to offer aid and support to the community rather than gather intelligence or enforce laws The final phase, “third cup of tea”, is when officers may broach the subject of terrorism, radicalisation and extremism through official talks/meetings (“ACT NOW”), interactive workshops (“Workshop to Raise Awareness of Prevent”, aka “WRAP”) or less formal discussions with small pockets of the community/individuals. The authors argue the success of this final stage hinges on the familiarity and trust built from previous stages Key to the strategy is engaging regularly, networking, and building relationships with community members and stakeholders before terrorism and radicalisation information is divulged
Lewandowski (2012)	Origins: Fusion centres are collaborative centres between two or more agencies that provide and share resources, intelligence, expertise and information in order to prevent, detect, or respond to criminal/terrorist activity. This dissertation focuses on Regional Operations Intelligence Centre (ROIC) which is the fusion centre for the New Jersey area, providing a ethnographic account of a “day in the life” of staff at the ROIC
	Operations, Materials, and Resources:
	Analysis Element which handles the distribution and creation of intelligence data, including threat analysis and crime analysis (see p. 11 for a diagram on the organisational structure) Threat watch desk, which falls under the threat analysis arm, where most of the collaboration between agencies and the public occurs. The purpose of the threat watch desk is to prepare reports and assessments when incidents occur or are planned and may involve “a potential nexus to homeland security and/or terrorism, potential deployment of law enforcement resources and high visibility” (p. 12) Office space is setup to facilitate communication and rapport‐building among staff (pp. 22–25 and 45–46). Daily morning meetings to discuss events over the last 24–72 h, pp. 26–27, p. 30) Soft management skills for leaders to building good atmosphere in a multiagency office (e.g., p. 35) “Common Operation Picture” (COP) which is a daily email the ROIC sends to public safety officials in New Jersey to disseminate accurate and timely information regarding the threat environment (p. 56 and Supporting Information Appendix D for content examples)
Mabrey et al. (2006)	Origins: Research, training development, and implementation was conducted by a collaboration of universities who then delivered training to over 60 agencies, including police
	Operations, Materials, and Resources:
	Research‐informed training manual regarding identification and interviewing techniques for terrorist threats and other border‐based crimes (e.g., organised crime). Specific detail on the content of the training is limited Lesson plans and training sites required, along with adaption of materials to local needs (e.g., features of the setting conducive to the crime problem)
Mesloh et al. (2003)	Origins: The University of Central Florida and the University of Central Florida Police Department developed an Amnesty Box initiative at public events to reduce risk (including radicalised violence)
	Operations, Materials, and Resources:
	Sealed container allowing individuals to discard contraband items before entering a metal detector or security checkpoint (including contraband that may facilitation violent extremism). Size: 30‐gallon plastic bins with swinging lids, taped to the bin. Shredded paper was placed at the bottom of the bin to absorb liquids Signage around the exterior of the auditorium explaining the purpose of the amnesty boxes (p. 8 for specific size and display requirements) Deployment of uniformed and plainclothes police in the area during the event to monitor patron behaviour Boxes removed after patrons had departed and were examined and documented in a safe location
Onyango (2018)	Origins: The Terrorism Amnesty Reintegration Program (ARP) was implemented by National Counter‐Terrorism Centre of Kenya (multiagency group including state agencies, collaborates with nonstate agencies) and offers amnesty to radicalised Kenyan youths who voluntarily surrender to Kenyan national security agencies (County Commissioners or police)
	Operations, Materials, and Resources:
	Originally 10‐day period and eventually extended indefinitely Returnees are monitored before and after integration to ensure sincerity (largely by Anti‐Terrorist Police Unit and National Intelligence Service) Returnees are provided with training, counselling and access to social services Returnees are provided with “reintegration kits” that contain tools which could aid economic independence (e.g., sewing machines) Courts required to swiftly and firmly punish returnees who defy rules for inclusion in the programme Programme numbers are capped to maintain monitoring and privacy of returnees (e.g., retaliatory attacks from violent extremist groups) Suggestion that formal implementation plan and legal framework would enhance implementation
Schanzer et al. (2016)	Origins: This study examines police‐community partnership and outreach in the US using interviews and focus groups to explore what works and what does not
	Operations, Materials, and Resources:
	Commitment from police chief and political leaders essential (p. 36) Community outreach separate to intelligence gathering and investigation (pp. 38–40) Police briefings/community forums with community members regarding terrorism Police working with community to create and deliver training products for police (p. 42, 45) Police outreach activities (e.g., attending community events)
van den Heuvel et al. (2012)	Orig ins: The HYDRA Immersive Simulation system is high‐fidelity training tool whereby leadership and decision‐making can be observed in controlled conditions.
	Operations, Materials, and Resources:
	For this study information regarding a simulated terrorist incident was revealed to participants in real‐time (throughout the course of 1 8‐h day), through a range of mediums (paper, video, graphic, actors, etc.) Each syndicate (group) is set‐up in a room, and are accompanied by a “loggist” who records all decisions made, and the rationales behind them Debriefing meetings to reflect on the multiagency management of the event and develop strategies to manage identified issues
Weine et al., 2017)	Origins: The Countering Violent Extremism Tailored Community Policing (CVETCP) initiative was developed in Los Angeles by police and community members
	Opera tions, Materials, and Resources:
	Comprised of five main practices, with communications conducted by LAPD's Liaison Bureau (est. 2008), comprised of ~6 police officers and 25 specialist volunteers and reserve officers (see p. 8). 1. Engagement: Community outreach officers meet with community leaders individually, build relationships with community organisations and religious groups Quarterly Muslim Forum held by LAPD and attended by Muslim organisation representatives (p. 4), interfaith events, engaging youths 2. Building Trust: Community outreach officers facilitate an open dialogue about sensitive issues with community members (includes police efforts to combat islamophobia) Crucial elements: providing a confidential space to talk, acknowledging past traumas (in countries of origin and the United States) that may have caused community distrust in police, publicly addressing islamophobia, listening to community perspectives/feedback, transparency, demonstrate open‐mindedness and helpfulness. 3. Educating: Building knowledge of hate crimes, police work, community resources, and violent extremism/counter violent extremism Includes interfaith education, CVE programme development, teaching communities about connecting with relevant resources, and law enforcement, education on nonviolent extremism issues (i.e., domestic violence and disaster preparedness) 1. Problem‐solving: Encouraging violent extremism prevention by facilitating community diffusion of tension (daily issues, as well as speech/hate crimes), teaching problem solving skills, providing workshops educating about hate crimes and how to cope as an individual and as a community, and providing technical assistance to communities 2. Mobilising: Encouraging civic engagement within the community, partner with NGOs to assist during crises, encourage civic engagement within the community to build a strong network, spread messaging countering that of violent extremist groups, support communities during crises, provide problem solving skills for addressing refugee and immigrant security issues, empower women and youth in the community to engage in leadership roles
Williams et al. (2016)	See “Included studies” section
Sandoval (2013)	Origins: This study uses a quantitative survey to explore what works and what does not with regard to information sharing behaviour between agencies.
	Operations, Materials, and Resources:
	Knowledge management, especially understandings and organisational perceptions of policy Emphasis on information sharing by leaders Emphasis on understanding of IT compatibility (including hardware, software, security systems and data standards) Interagency trust for safeguarding and protecting information shared with other agencies; time, reciprocity, understanding, security and timeliness all seen as vital Cultural emphasis on interagency partnerships from those in leadership roles Hindrances for information sharing may include organisational culture factors (e.g., attitudes or resistance from leadership, attitudes from colleagues), understandings of IT systems, lack of trust in other agencies with regard to lax safeguarding of IT systems, and policies

The enablers identified across the 16 studies include having available additional funding to support partnership work in a counterterrorism context and the need to have a dedicated coordinator to drive a multiagency initiative (Department of Homeland Security, 2016; Knight, 2009). Partnership work is also argued to improve when agencies are co‐located and there are convenient locations for meetings to take place (Department of Homeland Security, 2015; Williams et al., 2016). Ensuring agency participants have a clear understanding of programme goals and that these align with government priorities also helps to facilitate multiagency and interagency cooperation (Kerry, 2007; Schanzer et al., 2016). The need for organisational leadership by police is also identified as being important, indicating that senior levels of support help to facilitate partnership working, with political commitment also necessary (Australian National Audit Office, 2010; Braziel et al., 2015; Department of Homeland Security, 2015; Kerry, 2007; Knight, 2009; Lewandowski, 2012; Schanzer et al., 2016).

In the context of partnerships involving the provision of training, the reputation of the agency and its ability to provide good quality training was found to help ensure that training on counterterrorism and national security preparedness is adopted (Davis et al., 2016). Studies argued that training should not be one‐off, with participant follow‐up and opportunities for participants to link‐up regularly being identified as applicable to the sustainable impact of any training. This is further facilitated by having easy access to training products and activities that directly align with end‐user needs and priorities (Carter, 2006; Department of Homeland Security, 2016; Mabrey et al., 2006; Schanzer et al., 2016). Costs to initiate partnerships and provide products should also be kept low (Mesloh et al., 2003).

A number of the included studies note that it is important to have intelligence gathering activities separate and distinct from any community outreach activities aimed at countering violent radicalisation and/or extremism, ensuring that these two activities do not become blurred (Lamb, 2013; Schanzer et al., 2016; Williams et al., 2016; Weine & Younis, 2015). Police often require time to build relationships with community groups in order to foster transparency and meaningful engagement in this work (Lamb, 2013). It was also identified that having the same partners involved from start to finish of a partnership ensures consistency of participation (Lewandowski, 2012; Mabrey et al., 2006; Onyango, 2018; Schanzer et al., 2016; Williams et al., 2016).

Thirteen studies identified facilitators that were considered to be important for successful implementation of the intervention. These factors included access to funding, increased information sharing (two‐way process) and access to applicable intelligence databases and technology. In addition to access to required technology (e.g., Sandoval, 2013), included studies identified a need for administrative oversight of these databases and the importance of avoiding duplication of both activities and data collection (Department of Homeland Security, 2015, 2016; Knight, 2009). The studies also suggest that information should be targeted to the needs of participants and formal processes should be put in place to enable the efficient transfer of information to partners (Knight, 2009). Targeted information may take the form of both information that increases general awareness around terrorist risk, and also specific and accurate threat pictures delivered to stakeholders to allow them to make resource decisions about how to respond to national security threats in their jurisdictions (Department of Homeland Security, 2014, 2015; Kerry, 2007; Knight, 2009).

The quality of relationships with partners external to any partnership or partnership mechanism (e.g., a fusion centre) was identified by some studies as vital to collaboration and sharing of threat assessment information (Knight, 2009; Schanzer et al., 2016). These studies argued that police must engage in relationship building with stakeholders and that engagement should be broad in nature (Department of Homeland Security 2015, 2016; Mabrey et al., 2006.

Partnerships that have strong privacy, civil rights, and civil liberties policies and protections in place are considered crucial to success, especially in partnerships with many organisations such as in the case of fusion centres (Department of Homeland Security, 2015, 2016). Some included studies highlight the need for standardised assessment tools for defining the specific training needs across jurisdictions and training that is based on real scenarios (Kerry, 2007). Having checklists to assist partners to undertake relevant threat assessments was also identified as key (Mesloh et al., 2003). During critical incidents, having in place communication hotlines (i.e., priority communication channels) to speed up communication was suggested as important to agency responsiveness to terrorist events (Wurmb et al., 2018). In this context, it is suggested that partners should receive training around how to respond to critical incidents, with procedures in place to ensure consistent responses to incidents and interview suspects (Braziel et al., 2015; Kerry, 2007; Mesloh et al., 2003; Wurmb et al., 2018).

When working with partners, the included studies suggest that law enforcement needs to be transparent in their engagement, open‐minded and offer confidential spaces so partners can talk openly (Williams et al., 2016; Weine & Younis, 2015). Time, resources and energy should be invested into research and some studies argue that bureaucratic permissions need to be sought prior to the implementation of a partnership (Mabrey et al., 2006; Weine et al., 2017; Williams et al., 2016; Weine & Younis, 2015). When working and engaging with the community, some evidence indicates that police should focus less on law enforcement goals and more on community concerns, and be prepared to respond to thses (Lamb, 2012; Weine et al., 2017; Williams et al., 2016; Weine & Younis, 2015).

#### 5.4.4 Implementation considerations (barriers)

Of the 26 studies rated as a 3 for implementation on the EMMIE tool (see Tables 4 and 12), 16 studies discuss a range of obstacles encountered in implementing police multiagency interventions for countering radicalisation to violence. The first barrier identified was applicable to the delivery of training in national security preparedness and counterterrorism. Four studies examined partnerships that were concerned with national security/counterterrorism training (Carter, 2006; Davis et al., 2016; Kerry, 2007; van den Heuvel et al., 2012). The lack of ongoing support to supplement police training once the training is completed was identified as a barrier to implementation (Kerry, 2007), and agencies charged with providing training can face limited capacity to deliver their training to enough police and other personnel in the required timeframe (Davis et al., 2016). Further, a lack of clear organisation policies or a culture of blame can impact whether multiagency training simulations are derailed (van den Heuvel et al., 2012). Demands from funding agencies to deliver training in a short timeframe may mean that some police can miss out (Davis et al., 2016).

The second barrier was around administrative burdens and oversight requirements imposed on law enforcement partnerships. The included studies suggested that barriers to evaluations included a lack of access to relevant data to assess their effectiveness (Department of Homeland Security, 2015). These administrative burdens may inhibit the smooth operations of such partnerships (Knight, 2009). Internal bureaucratic processes and laws may also act to impede the sharing of information and intelligence, which is considered to be a critical component of multiagency working in a counterterrorism context (Mabrey et al., 2006). Studies highlight how the establishment of inadequate intelligence sharing systems and processes can be a problem (Department of Homeland Security 2015). This can be exacerbated by staff who are not committed to working with community groups and share intelligence with them (Lamb, 2012; Schanzer et al., 2016). Even if intelligence is shared with other agencies, the lack of adequate recording practices when intelligence is collected was identified as impacting on the ability of partners to action intelligence when it is passed on to them (Australian National Audit Office, 2010; Braziel et al., 2015; Lewandowski, 2012; Sandoval, 2013; van den Heuvel et al., 2012).

The third implementation barrier was the negative consequences of a rift between law enforcement and community goals around countering extremism (Schanzer et al., 2016). Some studies indicated that when partnering with community groups, there can be a tendency for police to adopt a narrow focus on extremism as mainly arising from Islamist extremism, which in turn implies Muslim communities are the problem (Schanzer et al., 2016; Williams et al., 2016). Study findings indicate that this can generate push‐back from communities to work with police (Lamb, 2013; Schanzer et al., 2016; Williams et al., 2016; Weine et al., 2017; Weine & Younis, 2015). A lack of willingness on the part of the police to learn about the concerns of the community and respond to them compounds this rift, particularly hampering the development of trust, which was identified as essential by a number of studies as a vital ingredient of effective multiagency partnerships to tackle terrorism (Lamb, 2013; Lewandowski, 2012; Onyango, 2018; Schanzer et al., 2016; Williams et al., 2016; Weine & Younis, 2015).

The fourth barrier that was identified by our review is high staff turnover, which can contribute to instability and lack of sustainability within multiagency partnerships (Department of Homeland Security, 2015, 2016). Two studies report issues with recruiting and/or retaining participants (Onyango, 2018; Williams et al., 2016). In the context of retaining participants in a partnership, it was identified that commitment and participation will be determined by the perceived value of being involved in a partnership, the required levels of commitment and other competing priorities faced by partners (Williams et al., 2016). Hence initiatives that counter violent extremism can be more attractive to participants when they find them personally satisfying, better than other competing alternatives, and have a personal investment in the initiative (Williams et al., 2016). Retaining participants is also influenced by difficulties that can arise when multiagency work requires agencies to integrate systems and change practices (Onyango, 2018).

Lastly, the fifth barrier to implementation is the time taken to develop and nurture multiagency partnerships, especially when fostering trust between agencies (Lamb, 2013; Schanzer et al., 2016; Williams et al., 2016; Weine & Younis, 2015). The need for investment in developing policies and practices, particularly those that prioritise community engagement and agency relationship‐building around the collection of intelligence, compounds the problem of time needed for multiagency partnerships to function efficiently (Lamb, 2013). The quality of pre‐existing relationships can help to overcome this time issue and this is particularly the case when police are required to work with community members or individuals who can help them identify individuals at‐risk of radicalisation (Williams et al., 2016). Poor pre‐existing relationships may inhibit communication and engagement, thereby lengthening the time it takes to establish functioning multiagency partnerships (Onyango, 2018; Schanzer et al., 2016; Williams et al., 2016; Weine & Younis, 2015).

#### 5.4.5 Economic considerations

Of the 181 studies deemed eligible for review Objective 2, four studies reached a rating of 3 or more on the EMMIE appraisal tool for examining economic considerations linked with multiagency interventions with police as a partner for countering radicalisation, extremism, and/or terrorism (Davis et al., 2016; Sandler et al., 2011; Stewart & Mueller, 2018; see Table 4). This indicates that most studies reporting at least some content pertaining to economic considerations either reported only direct cost and/or benefit estimates or both direct and indirect cost and/or benefit estimates, yet did not (a) provide estimates of marginal or total or opportunity costs and/or benefits (rating of 3); or (b) provide estimates of marginal or total or opportunity costs and/or benefits by bearer or recipient (rating of 4).

Davis et al. (2016) undertook a cost‐benefit analysis (CBA) of a State and Local Anti‐Terrorism Training (SLATT) programme delivered by the Institute for Intergovernmental Research, a nonprofit corporation based in Florida. The CBA is not a proper assessment of the law enforcement direct costs of SLATT training because it did not assess the costs and benefits to the agencies or personel who implemented the training (e.g., costs of planning, marketing and recruitment, or hosting the training). Instead, the evaluation involved measuring the costs and benefits of the programme to individual SLATT participants, rather than the cost‐benefit or cost‐effectiveness of the training to the public or taxpayers in relation to the monetary value of the training in decreasing terrorist risk.

The cost benefit analyses that were conducted drew from a cross‐sectional survey of programme participants and examined the costs individual participants incurred to participate in the SLATT training. The participants in the SLATT training programme included representatives from U.S. state, local and tribal law enforcement agencies. The training programme focused on “how to understand, detect, deter, and investigate acts of terrorism and violent criminal extremism by international and domestic actors” (Davis et al., 2016, p. 2). It comprised different components including onsite training, train‐the‐trainer workshops to facilitate in‐house training, online training modules and webinars, and specialised assistance (Davis et al., 2016).

The study authors used a choice experiment to assess what attendees deemed acceptable in terms of personal out‐of‐pocket costs, how they were reimbursed for their time (e.g., paid or asked to take a day off to attend), how far they had to travel, and how long the workshop was. The study quantified the inputs required for the SLATT intervention, which were measured as (a) the duration of the workshop in number of days (i.e., 1, 2, 3 or 4), (b) the proximity of workshop location to the requesting agency who was participating (e.g., <25, 25–100, or 100+ miles), (c) the scope of workshop topics (i.e., whether the focus was on domestic terrorism or international examples), or (d) whether there was a course registration fee ($0, $100 or $200). The authors found that while the SLATT training was delivered at no cost to participants (as it was covered by the Department of Justice), the participants still incurred some personal costs, but that this was outweighed by the perceived value of participating in different components of the programme. Average expenses per person for participants who received full departmental reimbursement ranged from $12 for participants from the train‐the‐trainer component to $22 for participants from the investigative/intelligence workshop. For those who were not fully reimbursed by their department, expenses averaged $86 per person for train‐the‐trainer participants and $168 for investigative/intelligence participants.

The remaining three studies examined economic considerations regarding the direct costs of implementation of the partnership programme and an estimate of the cost‐effectiveness and cost‐benefit (Sandler et al., 2011; Stewart & Mueller, 2018). Sandler et al. (2011) examined the monetary payback derived from Interpol's (International Criminal Police Organisation) efforts to coordinate counterterrorism measures amongst its member countries to arrest terrorists and weaken their capability to conduct operations. This study derived data from Interpol's Stolen and Lost Travel Documents database (SLTD), that comprises information on member agency requests to Interpol on the arrests of terrorist suspects and hits on positive matches relating to those suspected of terrorism offences (see Sandler et al., 2011). The SLTD is an Interpol resource provided to members that enhances communication and the coordination of international counterterrorism activities. Partners in the programme include Interpol's General Assembly of delegates from member countries, Interpol's Executive Committee (elected by General Assembly), member countries' national central bureaus (NCBs), the United Nations, and “all organisations, authorities, and services whose mission is to prevent or combat international crime” (Sandler et al., 2011, p. 82). The study made a range of assumptions and calculations relating to the estimated costs of a successful terrorist attack and the monetary benefits derived from Interpol's resources. The authors analysed different scenarios to calculate the costs and benefits and found that each dollar of Interpol counterterrorism spending returned approximately US$200 in benefit. The study stated that Interpol's total counterterrorism expense in USD was $13,506,843 (2006), $16,647,566 (2007) and $12,013,837 (2008) (Sandler et al., 2011). The study authors compared arrests and the theoretical absence of these arrests, relative to the costs associated with a successful terrorist attack. They reported the cost/benefit ratio across 12 scenarios and with two different conditions: cost/benefit ratios including only casualties, or casualties and Gross Domestic Product (GDP) benefits. The average benefit cost ratio for 2006 was $204.30 when including GDP, and $41.40 when only considering casualties. For 2007, the ratio value including GDP was $195.60, and $47.30 when only considering casualties (Sandler et al., 2011).

Stewart and Mueller (2013, 2014) undertook a CBA of the Australian Federal Police (AFP) airport counterterrorism policing in 2014 across Australia. These counterterrorism activities include Joint Airport Investigation Teams (comprising the AFP, state/territory police and customs/border force to target serious/organised crime in the aviation network), Joint Airport Intelligence Groups, which exist to provide dedicated intelligence support, police aviation liaison officers, airport police commanders, airport uniformed officers, and counterterrorism and first response teams (Stewart & Mueller, 2013). The study examined the cost‐effectiveness of the $90 million AFP airport counterterrorism policing budget in Australia (cost in 2014) against different probabilities of risk reduction (benefit) relating to a terrorist attack. These calculations were based on various terrorist threat scenarios to airports and aircraft, as well as calculated costs from government budget papers. The study authors concluded that AFP airport policing moves toward cost‐effectiveness if it reduces the risk of an stewar attack “by approximately 25% and if the probability of an attack at any airport in Australia exceeds 5% per year” (Stewart & Mueller,  2014, p. 113). The study concluded that if risk of a terrorist attack is reduced by 50%, with the annual threat probability of terrorist attack being 5%, this yields a net value of $76 million per year, with the benefit‐to‐cost ratio being 1.84. At that level, airport counterterrorism policing for the ten airports included in the study would be cost‐effective, with $1 of cost buying $1.84 in benefits.

Stewart and Mueller (2018) conducted a risk and economic analysis of various counterterrorism law enforcement strategies employed at U.S. airports, including partnerships involving police that comprised JTTF and Visible Intermodal Protection Response (VIPR) teams. JTTFs are coordinated by FBI and are a multiagency investigative taskforce (see Casey, 2004). VIPRs comprise teams "to augment local, state, and federal entities' efforts to enhance security on U.S. critical transportation infrastructure", including airports (Department of Homeland Security, 2012, p. 1). The direct cost of implementing the programme in 2016 was (in USD) $125 million for the JTTF and $20 million for the VIPR teams. The risk reduction and economic benefit was reported as a calculated percentage in risk reduction of a passenger‐borne bomb attack per year of spending on various initiatives. This study found that both JFFTs and VIPRs were cost‐effective in their level of risk reduction and cost. This included cost‐effectiveness data relating to the percentage of risk reduction in a passenger‐borne bomb attack per year of spending on these initiatives. For example, for the JTTF, risk reduction equalled 2.3% and for the VIPR Teams it equalled 0.37%.

## 6 DISCUSSION

Agencies working cooperatively together is an approach used to address the complex nature of radicalisation risk and pathways to violent extremism. This is because a single agency (particularly police), organisation or entity does not have the capability to address the various factors that lead to violent radicalisation. As such, multiagency partnerships may provide capacity to collectively address violent radicalisation in a holistic, coordinated and collaborative manner. Police involvement in these multiagency programs are theorised to disrupt pathways from radicalisation to violence because the police are often one of the first points of contact with individuals who have radicalised to extremism. The police are also the first point of call for those who are concerned about or report known associates, friends or family members as being at‐risk of radicalisation. Multiagency programs that involve policing in efforts to reduce radicalisation to violence and improve collaboration entail a range of partnership approaches. They can involve multiagency data and intelligence sharing which is assumed to increase the capacity of the partnerships to identify and then target people at‐risk of radicalisation and prevent acts of terrorism. Other approaches include agencies working in multiagency teams and receiving joint training that is assumed to improve interagency collaboration and coordinated responses to national security incidents.

### 6.1 Summary of main results

The review identified five studies that contributed to the assessment of the effectiveness of the multiagency approach, with one study assessing the impact on radicalisation to violence and the other four studies assessing the impact on collaboration outcomes. The single study that assessed the impact on radicalisation to violence was conducted in 2015 and was carried out in the United States. It was a Muslim‐led initiative involving police that aimed to counter violent extremism through a community‐based education and awareness programme. The programme aimed to improve referral networks for agencies/third parties to help assist individuals identified as at‐risk of radicalisation. Evidence from this study showed that the effect sizes were small to medium and favoured the treatment group, aside from one item that favoured the comparison group (survey item: “I make friends with people from other races”). These results need to be interpreted with caution for four reasons: (1) the survey was completed by the programme volunteers, and the authors did not specifically identify these volunteers as individuals at‐risk of radicalisation; (2) the survey items require testing with different samples to ensure the questions are indeed valid and reliable measures of radicalisation risk (3) it is not clear if the survey respondents had all been directly exposed to the two intervention components that involved police and (4) this study requires replication to allow for stronger conclusions, ideally using a randomised design. Overall, this study may not be a direct evaluation of how effective police‐involved multiagency interventions are for countering violent radicalisation.

Four studies met the inclusion criteria to assess the impact of multiagency interventions that aimed to increase collaboration. One study examined the impact of agency alignment with a TCL. The evidence from this study showed that greater alignment with the TCL showed small but positive effects for greater working relationships between organisations, more intelligence sharing, and more engagement with the FBI, as well as all levels of law enforcement agencies, and fusion centres. A second study assessed how the number of multiagency collaborative partners impacted perceptions of clarity and understanding of U.S. DHS funded strategies and stated goals. Evidence from this study suggests that a greater number of partners involved in a multiagency intervention is associated with more perceived clarity and understandings of missions, responsibilities and goals at the state and local level, but were conversely associated with less perceived clarity and understanding of federal DHS missions, responsibilities and goals. The third and fourth studies both examined the impact of grants from the DHS. One of these studies found when respondents perceived that homeland security grants had more influence on their agency's approach to homeland security, they reported that their agency engaged in fewer homeland security preparedness activities. The other study found that receipt of homeland security funding did not directly influence whether a policing agency engaged in *at least one* homeland security initiative, but for those policing agencies that did engage in at least one homeland security initiative, agencies that received grants engaged in more homeland security initiatives than those that did not receive grants.

Overall, the review finds that there is no clear evidence to determine if multiagency partnerships involving police are effective approaches for reducing radicalisation to violence. There exists only a very small amount of mixed evidence about the effectiveness of multiagency partnerships for improving collaboration through building better information sharing, opportunities for training and fostering shared understanding of missions and goals. It is important to note that these results are based on low quality evidence, and in some instances the measures rely on scales that also include nonterrorism‐related items. The results for the review of effectiveness should therefore be interpreted with a great deal caution.

To better understand the processes underpinning multiagency interventions that aimed to reduce radicalisation to violence, we conducted an assessment of mechanisms, moderators, implementation, and economic considerations using the realist synthesis informed EMMIE framework developed by the UK's *What Works for Crime Reduction Centre* (Johnson et al., 2015; Thornton et al., 2019). Twenty‐six studies met our criteria for synthesising the processes that facilitate or constrain implementation, the mechanisms underpinning the intervention, factors moderating the impact of the intervention, and/or the information about the costs and benefits of the programme. A possible mechanism underpinning police‐involved multiagency interventions for countering violent radicalisation may be the degree of coupling (or disconnect) between partnering agencies. Possible moderators of the impact of police‐involved multiagency interventions for countering violent radicalisation may be institutional pressures, availability of grants, the size of the law‐enforcement agency, and whether the law‐enforcement agency is positioned in a rural or urban setting.

Four studies examined cost‐benefits of police‐involved multiagency programs for countering violent extremism, with one focused on a training intervention and the other three focused on specialised task forces or teams or multiagency counterterrorism responses in airports. The training‐focused study suggests that the perceived value of training may encourage practitioners to engage in training regardless of their own personal cost to attend. The other three studies indicate that while the costs of specialised taskforces and multiagency counterterrorism strategies at airports are substantial, they can be effective in terms of monetary benefits and risk reduction.

One theme to emerge from the synthesis of implementation considerations includes the importance of multiagency teams taking time to build trust and develop shared goals between partners. This also means that police need to be open and transparent when engaging with community partners and act in a way that prioritises the needs of the community. The analysis also suggests that multiagency teams should not overburden staff with unnecessary administrative tasks. One important finding from the synthesis is that targeted and strong privacy provisions need to be in place for intelligence sharing across multiagency team members. There is also a need for multiagency teams to have access to ongoing support and training for the duration of the partnership.

### 6.2 Overall completeness and applicability of evidence

Just one study met our inclusion criteria to assess the impact of police‐involved multiagency programs on reducing radicalisation to violence, which prevents any generalisable conclusions from the results. As a consequence, it was not possible to conduct any analysis of publication bias or identify whether or not effectiveness varied by the intervention type, target, or location. This lack of evidence demonstrates a significant gap in understanding the impact of multiagency programs in being able to address the problem of radicalisation to violence.

Four studies met our inclusion criteria to assess the impact of police‐involved multiagency programs on the quality or nature of the multiagency collaboration, with little commonality amongst the evaluated interventions or outcomes. Indeed, no single effectiveness finding has been examined by two or more studies in this review, which means that these findings should be treated with the utmost caution. It is not reasonable to generalise from the results of the effectiveness studies, at least not until they have been replicated, ideally several times.

The focus on U.S. interventions also limits our capacity to generalise beyond the U.S. context. The review is also limited in that the located studies did not report on a wide range of interventions. The outcome measures reported in the included studies did not capture the types of collaboration outcome measures—such as frequency and quality of collaboration—that we had anticipated at the outset of the review.

A total of 181 studies reported on an empirical study of a police‐involved multiagency intervention for countering radicalisation to violence, with some level of evidence for mechanisms, moderators, implementation facilitators and barriers, and/or economic considerations. Yet only 26 studies were rated as providing more rigorous accounts of the mechanisms, moderators, implementation facilitators and barriers, and/or economic cost‐benefits that offer some practical and policy insights of police‐involved multiagency programs. Some potential practical and policy insights are gained from the presentation of more detailed evidence about the barriers and facilitators around the implementation of multiagency programs. By contrast, the limited information reported in these twenty‐six studies restricts our capacity to draw meaningful practical insights about the estimated costs and benefits of the programs or about the theorised mechanisms and moderators of the multiagency collaborations.

### 6.3 Quality of the evidence

All studies included in this review have substantial methodological issues and/or risk of bias. The extant evaluation evidence does not utilise randomised or rigorous quasi‐experimental methods, and only one of the studies identified for the review of effectiveness *specifically* focused on evaluating the effectiveness of police‐involved multiagency interventions to counter violent radicalisation. Although countering radicalisation to violence through multiple agencies working together is intuitive and argued to be a potentially valuable intervention approach (Bellasio et al., 2018; Schanzer et al., 2016), the current review did not locate sufficient evidence to determine whether or not police‐involved multiagency interventions have an impact on either reducing radicalisation to violence or improving the nature and quality of multiagency partnerships. Moreover, the quality of the evidence for mechanisms, moderators, and economic considerations is particularly limited, with scant articulation of theoretically informed hypothesis testing or detailed accounts of costs and benefits. To improve the quality of evidence on police‐involved multiagency interventions to counter violent radicalisation, there is a critical need to test this intervention approach using theoretically informed randomised and rigorous quasi‐experiments with matched control groups/areas.

Methods for evaluating counterterrorism initiatives are particularly challenging due to a range of factors, such as the hard‐to‐reach nature of the population and securing and maintaining stakeholder support for developing theoretically and methodologically rigorous evaluations (Deloughery et al., 2016). In addition to the lack of formal evaluations, programs that target radicalisation pathways to violence often lack appropriate methods and practices to measure impact or effectiveness (Koehler, 2017). Programs that adopt clear inclusion criteria and collect baseline data (Holdaway & Simpson, 2018) could increase the likelihood of robust evaluations on the impact of police‐involved multiagency responses to countering violent extremism. The existing body of evidence is largely a function of the fact that few programs in this area have been subject to any type of formal evaluation (see also Bellasio et al., 2018; Deloughery et al., 2016).

### 6.4 Limitations and potential biases in the review process

We did not identify any specific limitations or biases in the systematic review process. Although the review identified very few impact evaluations, the use of the GPD and a number of supplementary systematic search strategies reduces the likelihood that eligible evidence was not captured by the review.

Specific strategies were employed to maintain consistency and validity for the component of the review assessing moderators, mechanisms, implementation and economic considerations of the intervention (Objective 2), such as independent double‐coding and collaborative discussions about eligibility thresholds during the screening and coding process. However, syntheses of qualitative evidence have an inherent element of subjectivity, a challenge noted by Thornton et al. (2019) when reflecting on the development and application of the EMMIE framework.

One limitation of the findings is that the review includes research published by December 31st, 2018. The evaluation of multiagency interventions with police as a partner may be a rapidly evolving area of research, which may mean this review omits eligible studies conducted in 2019 and 2020. Therefore, it will be important that this review is updated within 2–3 years to capture any new research, which will ideally also facilitate more concrete conclusions about the effectiveness of multiagency interventions with police as a partner for reducing radicalisation to violence.

### 6.5 Agreements and disagreements with other studies or reviews

Due to the limited and mixed nature of evaluation and review literature on the effectiveness of police‐involved multiagency interventions aimed at reducing radicalisation to violence, the findings of this review do not reaffirm or contradict any existing review. One study included in this review (Williams et al., 2016) was also included in a review conducted by Mazerolle, Eggins et al. (2020) that assessed the effectiveness of policing approaches aimed at enhancing community connectedness to counter violent extremism. The interpretation of the Williams et al. (2016) study results are consistent across this review and the Mazerolle, Eggins et al. (2020) review.

## 7 AUTHORS' CONCLUSIONS

Currently, there is limited and mixed evidence that assesses the processes and impact of programs that use police‐involved multiagency approaches aimed at reducing radicalisation to violence. The evidence reported in the synthesis of implementation factors suggests that building trust and shared understandings of missions and goals is central to multiagency collaborations and that intelligence sharing is possibly the most valuable aspect of multiagency collaborations. Future research should aim to rigorously evaluate the impact and outcomes of multiagency partnerships.

### 7.1 Implications for practice and policy

Multiagency collaborations to reduce radicalisation to violence make intuitive sense because it is assumed that the complex process of radicalisation needs a multifaceted approach for addressing a variety of risk factors. It is assumed that police should be involved in such partnerships given they can be the first point of contact with individuals who have radicalised or are planning a terrorist attack. This review does not find sufficient evidence to assess whether or not these multiagency collaborations are effective.

A key finding from the review of mechanisms, moderators, implementation and economic considerations is that capability alignment of the agencies involved in a multiagency collaboration is an important consideration for policy and practice moving forward. Some evidence from this review suggests that when the capabilities of the various agencies are well aligned, then there might be a greater chance for better working relationships between partners, increased intelligence sharing, and improved engagement with other law enforcement agencies, than when the capabilities of participating agencies are not well aligned. This was observed in the U.S.‐based fusion centre approach. These various capabilities are related to prevention (including information gathering, recognition of warning indicators and intelligence analysis), protection (including critical infrastructure protection), responsiveness (including critical resource logistics and dissemination and onsite incident management), recovery (such as structural damage assessment and restoration of lifelines), as well as common capabilities such as planning and communications across agencies (see U.S. Department of Homeland Security, 2007). While the degree of alignment of capabilities may limit the breadth multiagency interventions, it needs to be balanced against the need to involve an optimal (not too large, not too small) number of partners in a collaborative programme. Policy makers should consider prioritising grant allocations to foster those collaborations that have shared law enforcement and community goals around countering radicalisation to violence, low levels of staff turnover, low levels of administrative costs, and high levels of trust between partners. However, these implications should be interpreted with caution given that they are drawn from a small sample of studies which are not rigorous impact evaluations and most of which have high risk of bias.

### 7.2 Implications for research

The field of research into approaches to reduce radicalisation has grown considerably (see Romaniuk, 2015; Thompson et al., 2020). Nevertheless, there remains limited efforts to use robust methods to evaluate multiagency approaches that seek to reduce radicalisation to violent extremism (for an exception, see Thompson et al., 2020). Four recommendations for future research emerge from this review.

First, researchers need to more deeply theorize about the mechanisms that underpin multiagency partnerships. This review, as well as a recent report by Thompson et al. (2020), find that there are a lot of unsubstantiated assumptions about why multiagency approaches are good for community safety, yet organisational theories are not well integrated into the logic models that underpin the purpose, capacities, functioning and stated outcomes of these collaborations. Researchers should work together with agencies implementing these multiagency programs at the early stages of the programs being formed so that theories of change (particularly organisational theories) can help inform the operations and outcomes of these collaborations.

Second, the review was unable to ascertain whether the effectiveness of police‐involved multiagency interventions varies by factors such as the geographicial location of the intervention, nature of the target population, or nature of the intervention, including the optimal number of collaborating partners. Further research is needed to determine whether the number of partners in a collaboration is too small, then the capacity of the partnership to access relevant intelligence and identify at‐risk individuals might be inadequate. Conversely, if the number of partners in a multiagency intervention is too large, then the collaboration may run the risk of facing difficulties in implementing efficient intelligence sharing processes, can lack mutual understandings of missions and goals between partners, be subject to high staff turnover, and encounter administrative burdens and demanding oversight requirements.

Third, this review found that there are many more process evaluation studies around multiagency approaches that seek to reduce radicalisation to violence than there are impact evaluations. Nevertheless, the quality of these process evaluations is generally poor with inconsistent exploration and reporting of basic process evaluation components (see Thompson et al., 2020). This was particularly highlighted by the EMMIE approach to qualitatively synthesising the process evaluation evidence. Similarly, a recent article by Thornton et al. (2019) concludes that all dimensions of EMMIE rarely receive equal coverage in published papers, with a lot of missing or incomplete data and information, with mechanisms rarely tested, and economic information and analysis poor or rarely undertaken. This suggests that future researchers should ensure evaluations of multiagency programs that aim to reduce radicalisation to violence have clearly articulated and explicit theories of change, that the evaluations collect, analyse and report cost‐benefit information, and that researchers ensure that moderators and implementation information is built into the process evaluation design from the outset.

Fourth, whilst recognising that process evaluations are important (see Thompson et al., 2020), the dearth of impact evaluations suggest a need for greater focus in this area. This recognises that conducting evaluation research in the area of countering violent extremism is particularly difficult (Koehler, 2017; Romaniuk & Chowdhury Fink, 2012).

## INFORMATION ABOUT THIS REVIEW

### Roles and responsibilities

- *Content*: Lorraine Mazerolle, Adrian Cherney, Elizabeth Eggins, Lorelei Hine, and Angela Higginson.
- *Systematic review methods*: Elizabeth Eggins, Angela Higginson, and Lorelei Hine.
- *Statistical analysis*: Elizabeth Eggins and Angela Higginson.
- *Information retrieval*: Elizabeth Eggins, Lorelei Hine, and Angela Higginson.

### Sources of support

This review is funded by a Campbell Collaboration grant awarded to Lorraine Mazerolle, Elizabeth Eggins, Adrian Cherney and Angela Higginson via Public Safety Canada.

We acknowledge the support provided by the following research assistants: Lynley Anderson, Kaliah Daley, Rebecca Dunne, James McEwan, Georgia Hassall, Monique Lynn, and Natalya Seipel.

### Declarations of interest

Three of the review authors have internal roles within the Campbell Collaboration Crime and Justice Group. Lorraine Mazerolle is the Co‐Chair of the Crime and Justice Coordinating Group (CJCG), Angela Higginson is the Editor of the CJCG, and Elizabeth Eggins is the Managing and Associate Editor of the CJCG. Consequently, Lorraine Mazerolle, Angela Higginson, and Elizabeth Eggins will not be involved in any editorial or internal Campbell Collaboration communications about this review. In addition, Adrian Cherney has published research that is closely linked with the review topic. To minimise potential bias, Cherney will not be involved in the screening or coding of any studies for this review.

### Plans for updating the review

Lorraine Mazerolle and Adrian Cherney will be responsible for updates of this review, which are anticipated to occur every 3–5 years.

## Supporting information

Supporting information

Supporting information

Supporting information

Supporting information

## Footnotes

1 We conducted a search of the literature using the following terms to identify existing reviews: terroris* OR extremis* OR radicali*. Searches of the following locations did not identify any existing systematic reviews (completed or ongoing) on the specific topic proposed in this proposal: Campbell Collaboration; Cochrane Collaboration; PROSPERO registry; Google Scholar.

2 Information provided via personal correspondence with study authors.

3 The relationships scale was an index variable comprising six subquestions asking agencies to report on their relationship with each of the six external agencies. Responses were measured on a four‐point Likert scale from 1 (no relationship) to 4 (very close relationship). Values on the index therefore ranged from 6 to 24, with higher values indicating a closer relationship with a larger number of agencies.

4 Both the provide and receive intelligence scales were index variables comprising six subquestions asking agencies to report on the frequency with which they received or provided intelligence to each of the six external agencies. Responses were measured on a four‐point Likert scale from 1 (very infrequently) to 4 (very frequently) for both indexes. Therefore, values could range from 6 to 24, with higher values indicating greater frequency of information sharing or receiving from a larger number of agencies.

5 Whilst the author extensively refers to this variable in text as the number of collaborations that the police agency had, the survey item was worded “Please check the following agencies/organisations that your local government collaborates with on homeland security issues. (Check all that apply)” (p.65). For the purposes of this review, we assume that the author is correct in interpreting “local government” as policing agency.

6 Information provided via personal correspondence with study authors.

7 A system that compiles information from law enforcement and other agencies into a central state repository (see https://www.dps.texas.gov/administration/crime_records/pages/texasdataexchange.htm).

8 The participants for this study are described in the “Review of quantitative effectiveness” section.
