# Supplementary material for: Multiagency programs with police as a partner for reducing radicalisation to violence
Source: Campbell Syst Rev. 2021 May 5;17(2):e1162. doi: 10.1002/cl2.1162 (PMC8356331; doi:10.1002/cl2.1162)
Supplement: Supplementary file 1 — Supporting information [file CL2-17-e1162-s001.docx]

## Appendix A: Systematic search record

Table A1: Grey literature search record

| **Source** | **Search date** | **Search strategy** | **N results** |
| --- | --- | --- | --- |
| *Global Terrorism Research Centre (Monash University)* | 11/02/2020 | No search functionality. Hand-searched the publications section of the website for publications mentioning police and/or a response to terrorism/radicalisation/extremism. | 21 |
| *Triangle Centre on Terrorism and Homeland Security* | 11/02/2020 | No search functionality. Filtered by “reports” and “journal articles” and date range 2002-2018. | 16 |
| *Department of Homeland Security* | 20/02/2020 | No search functionality in publications library. Filtered by “law enforcement partnerships” and hand-searched results for publications mentioning police and/or a response to terrorism/radicalisation/extremism within 2002-2018 date range. | 40 |
| *Public Safety Canada* | 19/02/2020 and 12/03/2020 | - Filtered by “counter-terrorism” in the Publications & Reports section. - Conducted two searches in the Public Safety Canada library with the same search terms (police OR policing OR “law enforcement” OR “law-enforcement”). Search 1 was in the title field and search 2 was in the subject field. Limiters applied: unchecked Commissioner’s Directions and DVDs under Sources, date range 2002-2018. - Conducted two searches in the Police Catalogue with the same search terms (terror* OR extrem* OR radicali* OR counter* OR de-radicali*). Search 3 was in the title field and search 4 was in the subject field. Limiters applied: unchecked Commissioner’s Directions and DVDs under Sources, date range 2002-2018. | 1,382 |
| *National Consortium for the Study of Terrorism and Responses to Terrorism (START)* | 11/02/2020 | No search functionality. Filtered by topic area “policing terrorism” and date range 2002-2018. | 85 |
| *Terrorism Research Centre* | 17/02/2020 | No search functionality. Hand-searched the publications section of the website for publications mentioning police and/or a response to terrorism/radicalisation/extremism within date range 2002-2018. | 2 |
| *Global Centre on Cooperative Security* | 11/02/2020 | Could not narrow down search via search filters (e.g., document type, country, author), and no text search functionality. Hand-searched the publications section of the website for publications mentioning police and/or a response to terrorism/radicalisation/extremism within date range 2002-2018. | 37 |
| *Hedayah* | 11/02/2020 | No search functionality on reports and publications page. Hand-searched the publications section of the website for publications mentioning police and/or a response to terrorism/radicalisation/extremism within date range 2002-2018. | 4 |
| *RAND Corporation* | 20/02/2020 | No search functionality. Filtered by topic area “terrorism” and category “research”. Hand-searched results for publications mentioning police and/or a response to terrorism/radicalisation/extremism within date range 2002-2018. | 30 |
| *Radicalisation Awareness Network (RAN)* | 17/02/2020 | No search functionality. Hand-searched the publications section of the website for publications mentioning police and/or a response to terrorism/radicalisation/extremism within date range 2002-2018. | 24 |
| *RadicalisationResearch* | 11/02/2020 | No search functionality. Examinations of this website revealed that the research indexed was predominantly journal articles from journals already captured by the systematic search. | 0 |
| *Royal United Services Institute (RUSI)* | 19/02/2020 | Not search functionality Filtered by topic area “terrorism” and date range 2002-2018. | 1 |
| *Impact Europe* | 19/02/2020 | No search functionality. Filtered by “internal publications” and “external publications” and date range 2002-2018. | 5 |
| *National Criminal Justice Reference Service* | 12/03/2020 | Due to limited search and export functionality, specific indexing terms provided by NJCRS that were related to terrorism were selected and all results displayed were manually added to and EndNote library. | 100 |
| *Terrorism Research Centre (University of Arkansas)* | 13/11/2020 | This website directs users to the START, which is already included in the search. | - |
| *International Association of Law Enforcement Intelligence Analysts* | 13/11/2020 | The journal for this association is included in the GPD systematic search and so was not searched separately for this review. | - |
| *Naval Post-Graduate School* | 13/11/2020 | Theses from this source are indexed in ProQuest Dissertation and Theses Global Database, which is included in the GPD systematic search. Therefore, this source was not searched separately for this review. | - |

Table A2: Journal hand searches, trial registries, and expert consultation

| **Search component and source** | **Search date** | **Search strategy** | **N results** |
| --- | --- | --- | --- |
| Journal hand search:   - Critical Studies on Terrorism - International Journal of Conflict and Violence - Policing—An international Journal of Police Strategies and Management - Policing and Society - Sciences of Terrorism and Political Aggression - Studies in Conflict & Terrorism - Terrorism and Political Violence | 05/03/2020 | Using Web of Science platform, refined to the specific journal titles, searched the TOPIC field (captures title, abstract and subject fields) using the following search string: (police* OR policing OR “law enforcement”). Search was limited to the 12-months prior to 31^st^ December 2018. | 13 |
| Journal hand search:   - Dynamics of Asymmetric Conflict - Journal of Policing, Intelligence and Counter Terrorism | 05/03/2020 | Using Taylor and Francis platform, refined to the specific journal titles, searched on the title and keyword fields using the following search string: (police* OR policing OR “law enforcement”). Search was limited to the 12-months prior to 31^st^ December 2018. | 3 |
| Journal hand search:   - Journal for Deradicalization - Perspectives on Terrorism | 05/03/2020 | Using Directory of Open Access Journals platform, refined to the specific journal titles, searched on the title and keyword fields using the following search string: (police* OR policing OR “law enforcement”). Search was limited to the 12-months prior to 31^st^ December 2018. | 1 |
| Trial registries:   - World Health Organisation (WHO) International Trial Registry - Trial registries indexed on the Office for Human Research Protections website <https://www.hhs.gov/ohrp/international/clinical-trial-registries/index.html> | 05/03/2020 | WHO trial registry captured by the GPD systematic search. Five trial registries not captured by the WHO registry, but listed in on the Office for Human Research Protections website were individually searched using the same policing search terms used for the GPD systematic search (Health Canada Clinical Trial Database; Swiss National Clinical Trials Portal; Philippine Health Research Registry; South African National Clinical Trials Register; Tanzania Clinical Trial Registry) | 0 |
| Consultation with experts (*n* = 44)   - Campbell Crime and Justice Coordinating Group Steering Committee - Authors of Department of Homeland Security funded reviews (Sarah Carthy, Michael Wolfowicz) - Members of the Department of Homeland Security and Public Safety Canada Advisory Committee for suite of funded Campbell Collaboration reviews on countering violent extremism - International colleagues of Professors Mazerolle and Cherney who conduct research in the area of countering violent extremism and/or community-oriented policing approaches | 10/03/2020 | A truncated copy of the protocol, PRISMA flowchart, and a list of excluded topic relevant studies were emailed by Professors Mazerolle and Cherney | 0 |

Se
