# Supplementary material for: Multiagency programs with police as a partner for reducing radicalisation to violence
Source: Campbell Syst Rev. 2021 May 5;17(2):e1162. doi: 10.1002/cl2.1162 (PMC8356331; doi:10.1002/cl2.1162)
Supplement: Supplementary file 3 — Supporting information [file CL2-17-e1162-s004.docx]

## Appendix C: Full-Text Coding Form^[[1]](#footnote-1)^

### General Study Details

*Note to coder.* Record information in this section for all studies, irrespective of whether the study will be used for the quantitative effectiveness component of the review of the qualitative narrative synthesis.

1. Study ID [textbox]

2. Report ID [textbox]*

3. What type of document is this study? [*dropdown menu*]

- 1. Peer-reviewed journal article
  2. Book chapter
  3. Dissertation
  4. Conference presentation
  5. Government report, technical report, or working paper
  6. Other (specify in textbox)

4. In what country was the intervention implemented? [*textbox*]

5. In what year was the intervention implemented? [*textbox*]

6. If the evaluation and/or intervention was funded, record the funding source. [*textbox*]

*SysReview allows for multiple reports of a single study to be included in the one full-text coding record form. Each report is nested within the overall study record and the Report ID will consist of the Study ID followed by a unique alphabetical code (e.g., 1234_a, 1234_b…).

### Coding Domains for Impact Evaluation Studies

*Note to coder.* Record information in this section for only impact evaluations that will be used in the quantitative effectiveness component of the review.

**Participants**

1. Who are the participants? [*checkboxes*]
   1. Citizens
   2. Practitioners (specify in textbox)
   3. Micro places (specify in textbox)
   4. Macro places (specify in textbox)
   5. Other (specify in textbox)
2. How were participants recruited? [*textbox*]
3. What were the eligibility criteria for inclusion in the study? [*textbox*]
4. Describe sample attrition. [*textboxes*]

| **Number of Participants** | **Treatment** | **Comparison** | **Total** |
| --- | --- | --- | --- |
| Referred to study |  |  |  |
| Consented |  |  |  |
| Assigned |  |  |  |
| Began intervention |  |  |  |
| Completed intervention |  |  |  |
| Completed follow-up 1 |  |  |  |
| Completed follow-up 2 (if applicable) |  |  |  |

1. Describe the characteristics of the sample. [*textboxes*]

| **Number of Participants** | **Treatment** | **Comparison** | **Total** |
| --- | --- | --- | --- |
| Age (M, SD, range) |  |  |  |
| Gender (% female) |  |  |  |
| Ethnicity (proportions) |  |  |  |
| Socioeconomic status (proportions) |  |  |  |

1. Record any other pertinent sample information for both the treatment and comparison groups. [*textbox*].

**General Methodological Details and Nature of Comparisons**

1. What is the nature of the comparisons for this study? [*dropdown menu*]
   1. Single intervention contrasted with single comparison condition
   2. Multiple interventions against a single comparison condition
   3. Within one group over time
   4. Other (specify in textbox)
2. General research design classification [*dropdown menu*]
   1. Randomised controlled trial
   2. Quasi-randomised controlled trial
   3. Non-randomised controlled trial (e.g., interrupted time-series, matched control group design)
   4. Other (specify in textbox)
3. What type of comparison condition was used? [*dropdown menu*]
   1. No treatment
   2. Treatment-as-usual (specify in textbox)
   3. Alternative treatment (specify in textbox)
   4. Waitlist control
   5. Other (specify in textbox)
4. How were treatment and comparison groups formed? [*dropdown menu*]
   1. Random allocation
   2. Matching (specify matching method and matching variables in textbox)
   3. On basis of score on a specific measure (e.g., diagnosis, specify in textbox)
   4. Self-selection
   5. Other (specify in textbox)
   6. Unclear
5. What was the unit of allocation? [*dropdown menu*]
6. Participant
7. Dyads
8. Family
9. Service site
10. Other (specify in textbox)
11. Unclear
12. If participants were randomly allocated to conditions, how was this implemented? [*dropdown menu*]
13. Simple
14. Yoked pairs
15. Cluster (specify cluster in textbox)
16. Block/stratified (specify variables in textbox)
17. Matched pairs (specify matching variables in textbox)
18. Other (specify in textbox)
19. Unclear
20. Not applicable
21. Who executed the randomisation? [*dropdown menu*]
22. Researchers
23. Practitioners
24. Other (specify in textbox)
25. Unclear
26. If applicable, was randomisation equivalent across intervention sites? [*dropdown menu*]
27. Yes
28. No
29. Unclear
30. Not applicable
31. Was group equivalence assessed? [*dropdown menu*]
32. Yes (specify how this was done in textbox)
33. No
34. Unclear
35. Not applicable
36. Were the treatment and comparison groups equivalent at baseline? [*dropdown menu*]
37. Yes
38. No (specify differences)
39. Unsure
40. Not applicable
41. Are there any differences between participants who completed versus did not complete the treatment? [*dropdown menu*]
42. Yes (specify differences)
43. No
44. Unsure
45. Not applicable
46. What was the unit of analysis? [*dropdown menu*]
47. Participant
48. Family
49. Service site
50. Other (specify in textbox)
51. Unclear

**Intervention Details**

- 1. What is the name of the intervention(s), as reported by study authors? [*textbox*]
  2. What settings were used during the intervention(s) (e.g., community, institutions etc)? [*textbox*]
  3. Not including police, how many partners where part of the intervention? [*textbox*]
  4. Specify the exact partners who were part of the intervention [*textbox*].
  5. When was the intervention conducted (e.g., year)? [*textbox*]
  6. Describe the intervention provided to participants, ensuring you record the specific components or materials implemented and the mode of implementation. [*textbox*]
  7. Describe the duration of the entire intervention. If reported, describe the minimum, maximum, mean and standard deviation for intervention duration. [*textbox*]
  8. Describe the intensity of the intervention (e.g., frequency of contacts and length of contacts). If reported, describe the minimum, maximum, mean and standard deviation for intervention intensity. [*textbox*]
  9. Who implemented the intervention? [*textbox*]
  10. Was there more than one intervention site? [*dropdown menu*]
  11. Yes (specify number of sites in textbox)
  12. No
  13. Unclear
  14. Was treatment integrity monitored? [*dropdown menu*]
  15. Yes (specify in textbox)
  16. No
  17. Unclear
  18. Were there any issues with fidelity? [*dropdown menu*]

1. Yes (specify in textbox)
2. No
3. Unclear
   1. Did the authors report cost-benefit data? [*dropdown menu*]
4. Yes (specify in textbox)
5. No
6. Unclear

**Outcome(s) Measurement***

*To be completed for each eligible outcome within a study (or group of reports for a study). To add another outcome, click the ‘Add another outcome’ button located at the bottom of the screen.

- 1. What is the outcome being measured? [*textbox*]
  2. How was the outcome data gathered? [*dropdown menu*]
  3. Self-report
  4. Observation
  5. Official source
  6. Interview
  7. Other (specify in textbox)
  8. What are the psychometric properties of the measurement tool (e.g., reliability, validity, diagnostic thresholds, what higher /lower values mean)? [*textbox*]
  9. Who was the respondent/participant? [*textbox*]
  10. At what time-point(s) was the outcome measured? [*textbox*]
  11. Were data collected in the same manner for the treatment and comparison conditions? [*dropdown menu*]
  12. Yes
  13. No (specify in textbox)
  14. Unclear
  15. Which condition does the raw difference/effect favour (ignore statistical significance)? [*dropdown menu*]
  16. Experimental condition
  17. Comparison condition
  18. Neither condition (no difference)
  19. Unclear
  20. In which direction did the outcome change? [*dropdown menu*]
  21. Positive
  22. Negative
  23. Mixed (specify in textbox)
  24. Unclear
  25. Were there statistically significant differences for this outcome? [*dropdown menu*]
  26. Yes
  27. No
  28. Not tested
  29. Unclear
  30. What were the study author(s)’ conclusions about this outcome? [*textbox*]

**Effect Size Data***

*To be completed for each eligible outcome within a study (or group of reports for a study). To add another outcome, click the ‘Add another outcome’ button located at the bottom of the screen.

- 1. On what page number is the effect size data reported? [*textbox*]
  2. What type of effect size is being coded? [*dropdown menu*]
  3. Post-intervention only (first point of measurement after intervention)
  4. Baseline and post-intervention or pre-test measure prior to intervention)
  5. Follow-up (subsequent point of measurement after first post-test)
  6. What is the timeframe captured for the measure?
  7. Minimum [*textbox*]
  8. Maximum [*textbox*]
  9. Mean [*textbox*]
  10. Same for all participants (i.e., fixed) [*textbox*]
  11. How was the effect size obtained for this outcome? [dropdown menu]
  12. Reported in document → Go to Question 5
  13. Calculated by user → Go to Question 6
  14. Identify the type of effect size reported for this outcome and enter the required data for that effect size in the text boxes provided. [*textboxes*]

**
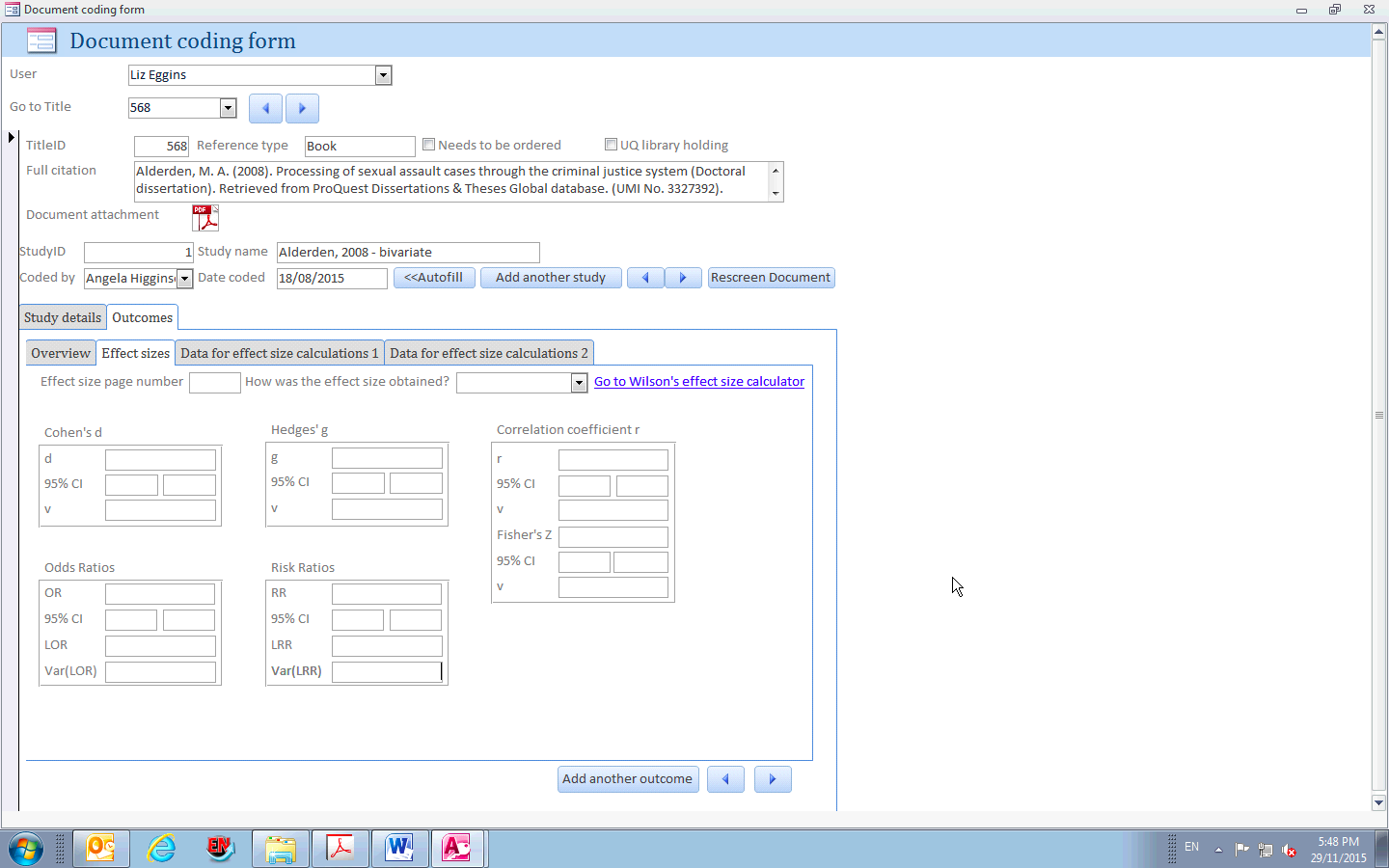
**

- 1. Enter the appropriate data in the relevant ‘Data for effect size calculations’ tabs (see below). The data entered will depend on what is reported in the document. If none of the circumstances in the tabs reflect the data in the document, follow the link to David Wilson’s online effect size calculator to calculate an effect size. You can enter the data in the ‘Data for effect size calculations 2’ tab in the ‘Other information’ textbox. [*textboxes*]


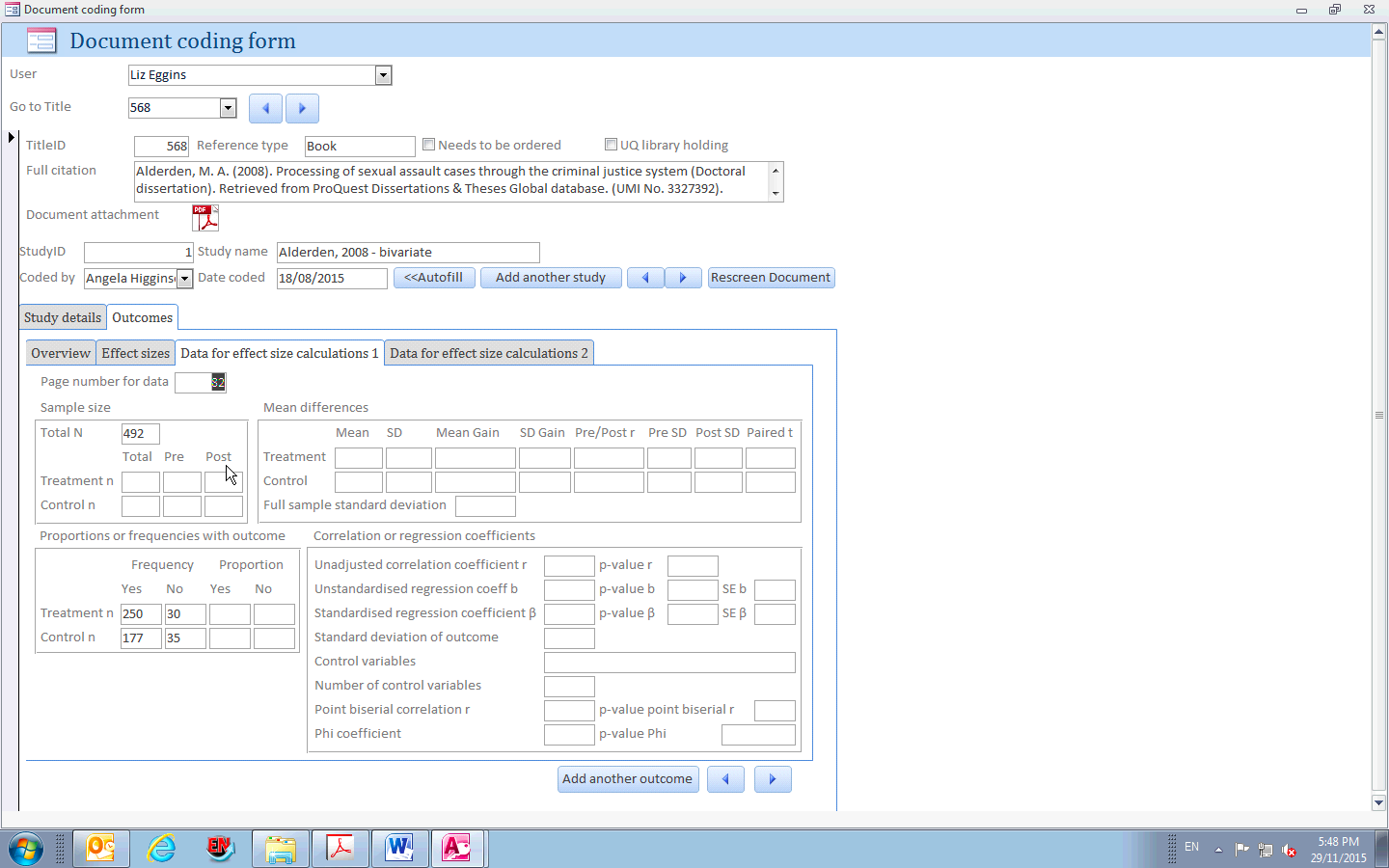


**
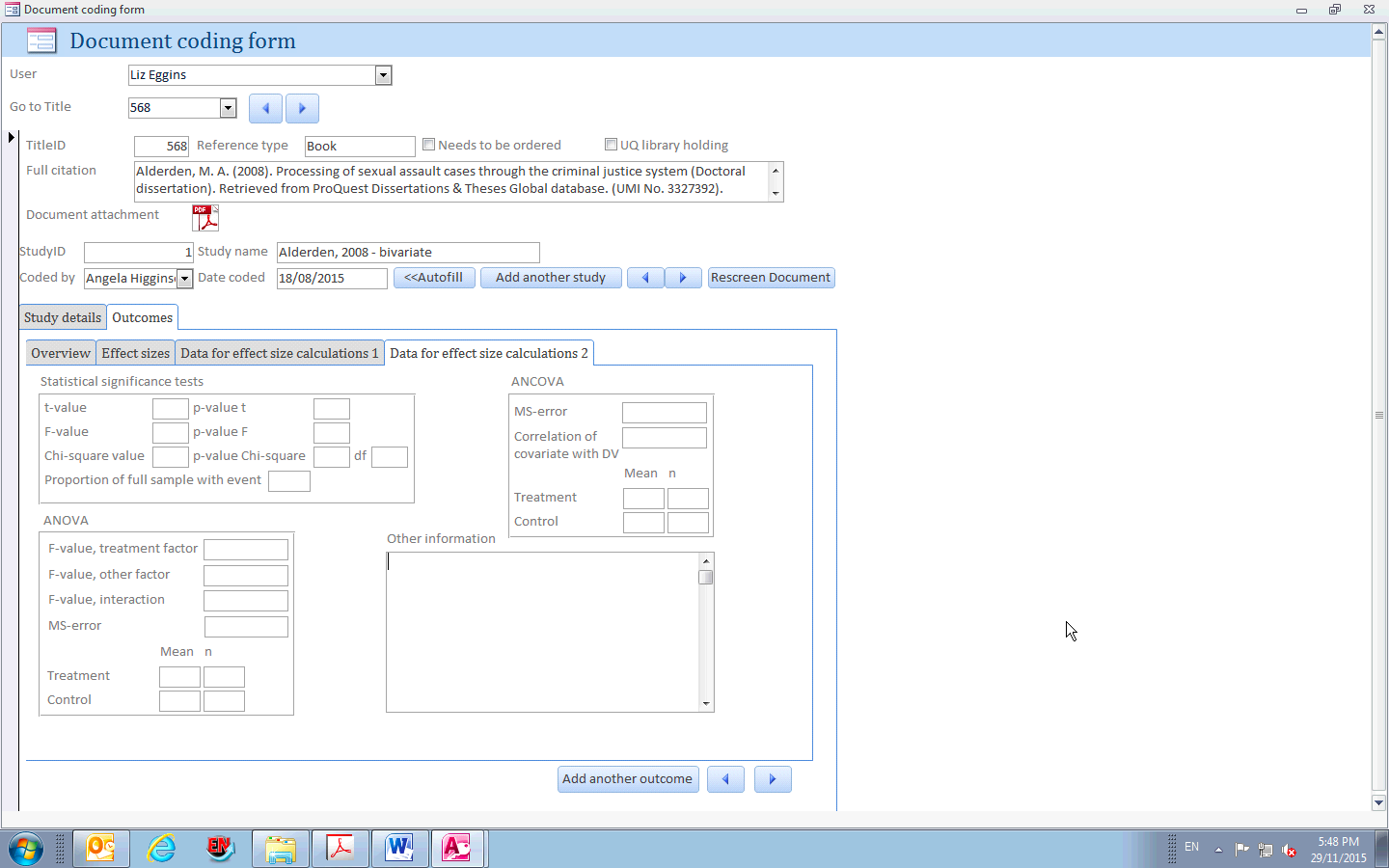
**

***Note to coder:*** Proceed to the Mechanisms, Moderators, Implementation and Economic coding tabs if this study is identified as requiring this type of coding.

### Coding Domains for Qualitative Synthesis

***Note to coder.*** For all studies, begin with the relevant *CASP Checklist* section. For studies only being used in the qualitative synthesis, then proceed to the *General Methodological Details* section. For studies included in the quantitative effectiveness component of the review, begin at the *Mechanisms* section.

**General Methodological Details and Nature of Comparisons**

1. General research design classification [*dropdown menu*]
2. Randomised or quasi-experimental trial with qualitative outcome data only
3. Single group pre-post design with qualitative or quantitative outcome data
4. Raw unadjusted correlational design where the variation in the level of the intervention is compared to the variation in the level of the outcome
5. Cross-sectional survey study
6. Process evaluation with both quantitative and qualitative data
7. Process evaluation with only qualitative data
8. Case study with quantitative and/or qualitative data
9. Other (specify in textbox)
10. What is the nature of the comparisons for this study (if applicable)? [*dropdown menu*]
11. Single intervention contrasted with single comparison condition
12. Multiple interventions against a single comparison condition
13. Within one group over time
14. There are no comparisons between conditions
15. What type of comparison condition was used? [*dropdown menu*]
16. No treatment
17. Treatment-as-usual (specify in textbox)
18. Alternative treatment (specify in textbox)
19. Waitlist control
20. Other (specify in textbox)
21. Not applicable
22. How were treatment and comparison groups formed? [*dropdown menu*]
23. Random allocation
24. Matching (specify matching method and matching variables in textbox)
25. On basis of score on a specific measure (e.g., diagnosis, specify in textbox)
26. Self-selection
27. Other (specify in textbox)
28. Unclear
29. Not applicable

**Participants**

1. Who are the participants? [*checkboxes*]
2. Citizens
3. Practitioners (specify in textbox)
4. Organisations (specify in textbox)
5. Micro places (specify in textbox)
6. Macro places (specify in textbox)
7. Other (specify in textbox)
8. How were participants recruited? [*textbox*]
9. What were the eligibility criteria for inclusion in the study? [*textbox*]
10. Record any other pertinent sample information. [*textbox*].

**Intervention Details**

1. What is the name of the intervention(s), as reported by study authors? [*textbox*]
2. What settings were used during the intervention(s) (e.g., community, institutions etc)? [*textbox*]
3. Not including police, how many partners where part of the intervention? [*textbox*]
4. Specify the exact partners who were part of the intervention [*textbox*]
5. Describe the intervention provided to participants, ensuring you record the specific components or materials implemented and the mode of implementation. [*textbox*]
6. Describe the duration of the entire intervention. If reported, describe the minimum, maximum, mean and standard deviation for intervention duration. [*textbox*]
7. Describe the intensity of the intervention (e.g., frequency of contacts and length of contacts). If reported, describe the minimum, maximum, mean and standard deviation for intervention intensity. [*textbox*]
8. Who implemented the intervention? [*textbox*]
9. Was there more than one intervention site? [*dropdown menu*]
10. Yes (specify number of sites in textbox)
11. No
12. Unclear
13. Was treatment integrity monitored? [*dropdown menu*]
14. Yes (specify in textbox)
15. No
16. Unclear
17. Were there any issues with fidelity? [*dropdown menu*]
18. Yes (specify in textbox)
19. No
20. Unclear

**Mechanisms**

*Note to coder.* Mechanisms generally refer to the means or variables through which the intervention works to generate changes in the relevant outcome. They link with articulations of the theory of change, an can include single variable or a sequence of events that occur to produce the *effect*. In other words, mechanism refers to the causal pathway through which an intervention makes an impact on the participants or area. Studies might discuss or report on mechanisms in different ways. At a minimum it might be referred to as ‘increasing effort’ mechanism, describing how a crime prevention measure is designed to produce the sought-after outcome. By contrast, a study might provide a detailed narrative of ‘how’ an intervention is hypothesised to work without necessarily using the term ‘mechanism’. An example for this review might be a police-involved multiagency intervention which aims to increase detection of those at-risk of violent extremism and the mechanism might be the quality of the referral process between agencies.

1. Does the study report on empirical data (qualitative or quantitative) that may be a mechanism for police-involved multiagency interventions for reducing terrorism, extremism or radicalisation?
   1. Yes (specify exact mechanism variables^[[2]](#footnote-2)^ in textbox)
   2. No (proceed to Moderators section)
2. Is there an articulation of the theory of change? This includes sets of testable intermediate (or proxy) measurable variables and patterns that would be observed in the data. In other words, is there consideration of the steps involved in activating a particular mechanism?
   1. Yes (describe this in textbox)
   2. No
3. Is there a map or logic model of possible mechanism(s)?
   1. Yes (specify page number)
   2. No
4. Did the authors collect and analyse data to test the mechanism(s)?
   1. Yes (specify how data was collected and analysed in textbox)
   2. No
5. How did the authors measure the mechanism(s)? [*textbox*]
6. What did the authors conclude about the mechanisms after analysis? [Textbox] (enter ‘Not applicable’ if the authors did not conduct any analysis).

**Moderators**

*Note to coder.* Moderators refer to those conditions or contextual factors that influence the activation of the mechanism (or the causal chain of events that lead to the effect). For example, the mechanism of 'shaming' for mandatory arrest policies for domestic violence seems to be moderated by the type of population. It appears to fire for middle-class populations who have a loss of status by the public shaming, whereas it appears to backfire for other populations, who arguably have less to lose. Other moderating conditions might be geographical area, crime type, time period, age group, or gender. In the context of multiagency interventions, moderators might be the nature of organisations involved (e.g., number and size of organisations, governmental v. non-governmental) or level of funding available. Remember, moderators are **pre-existing characteristics** that are difficult to modify by the implementers of the intervention, or the evaluation team. Factors that can be manipulated are usually considered to fall more in the **IMPLEMENTATION** section (although there is a fine line between these).

1. Does the study report on empirical data (qualitative or quantitative) that may be a moderator for police-involved multiagency interventions for reducing terrorism, extremism or radicalisation?
   1. Yes (specify exact moderator variables in textbox)*
   2. No (proceed to Implementation section)

* Note: To answer ‘Yes’, the study must mention factors that are considered causally relevant to the activation of the mechanism(s) through which the intervention under review is expected to work. These are factors that are a pre-existing condition (i.e. age, gender of the participants) rather than a factor to do with the study. The term *causally relevant* is key: reference to factors that are not reasonably expected to influence the outcome patterns (such as signs of the zodiac on responsivity to drugs) would not be appropriate for answering ‘Yes’.

1. Did the authors collect and analyse data to test the moderator(s)?
   1. Yes (specify how data was collected and analysed in textbox)
   2. No
2. How did the authors measure the moderator(s)? [*textbox*]
3. What did the authors conclude about the moderator(s) after analysis? [Textbox] (enter ‘Not applicable’ if the authors did not conduct any analysis).

**Implementation**

*Note to coder.* Implementation refers to the process of turning a working program theory into a practical successfully running program. Implementation can be complex and there are many things that might cause implementation failure along the way (e.g., resources, logistics, legalities, community support). Implementation factors can relate to the context in which the program is to be implemented, those that are expected to implement the program, those that are intended to receive it, and the program itself. Factors of the program that are able to be manipulated by the implementers or actors (e.g., study design, follow-up period, outcome data, what areas/people, what blinding procedure, what 'buy-in' from front-line staff) can be considered as **IMPLEMENTATION** factors. Those conditions that are pre-existing (i.e., not manipulatable) are considered MODERATORS.

Implementation can be thought of as dynamic factors that are introduced by the intervention (e.g. to do with the implementers or the delivery of the intervention). It is the *who* did *what* and *how.* Most crime reduction schemes involve several partners and occur in multiple stages. Miscommunication, misunderstanding, misspecification and combinations thereof at any stage in this process can result in sub-optimal results, and in extreme cases backfire effects. Understanding the process involved in turning a working theory into a practical successfully running programme is therefore very useful information, particularly for the purposes of scale-ups and replications.

1. Does the study report on empirical data (qualitative or quantitative) that is an implementation consideration for police-involved multiagency interventions aimed at reducing terrorism, extremism or radicalisation?
   1. Yes
   2. No (proceed to Economics section)
2. Does the study provide a description of what was implemented in practice?
   1. Yes (describe this in textbox)*
   2. No

To answer yes, the study must provide at least some basic details of the key stages involved in implementing a particular scheme effectively. More thorough reporting will include extensive information on both the key stages but also the people involved at different stages and some of the challenges and resolutions encountered at such stages. **Note** that implementation issues involve actions and conditions that are under the control of the actors involved (in other words are things that are dependent on the action of people). We are meaning here the choices and actions of the people actually responsible for producing the required output.

1. Does the study discuss enablers encountered in attempting to implement the intervention?
   1. Yes (specify these in the textbox)
   2. No
2. Does the study discuss obstacles encountered in attempting to implement the intervention?
   1. Yes (specify these in the textbox)
   2. No
3. Does the study identify what is crucial to successful implementation of the intervention?
   1. Yes (specify in the textbox)
   2. No
4. Does the study report any issues with recruiting and/or retaining participants?
   1. Yes (specify in the textbox)
   2. No
5. Does the study report any issues with implementing an evaluation of the intervention?
   1. Yes (specify in the textbox)
   2. No
   3. Not applicable

**Economics**

*Note to coder.* The economics domain refers to the costs and benefits of running the intervention. These may fall on a number of stakeholders/actors, and take a variety of forms. Preferably these costs and benefits are monetised - that is, a cash value is associated with them - but this is not as common as we would like. Please record any mention of costs and/or benefits to any stakeholder in this section. If the study refers to another document for costs then please note that down, as sometimes that is the only source of economic information.

1. Does the study discuss costs and benefits of the intervention?
   1. Yes (specify exact page number(s))
   2. No
2. Does the study quantify or describe the inputs required for the intervention?
   1. Yes (describe this in textbox)*
   2. No

*This should detail what resources, and how much of them were involved in the implementation of the program. Please detail all inputs (e.g. staff time, hardware purchased, etc) and quantities if reported.

1. Does the study quantify or describe the actual outputs for the intervention?
   1. Yes (describe this in textbox)*
   2. No

*This should detail what resources, and how much of them were actually utilised.

1. If ‘Yes’, can you calculate a cost per unit of output? (e.g., cost per hour of specialised practitioner)?
   1. Yes (report this in textbox)
   2. No
2. Does the study report the direct costs of implementation incurred by the implementers?
   1. Yes (specify these in the textbox)
   2. No
3. Does the study report the indirect costs of implementation incurred by the implementers?
   1. Yes (specify these in the textbox)
   2. No
4. Does the study explicitly report an estimate of cost-effectiveness per unit output?*
   1. Yes (specify in the textbox)
   2. No

* If reported in the review, please report any data supplied. If reported, cost effectiveness estimates should be reported for two or more scenarios (e.g. the status quo or two different interventions) and might include: 1) the estimated cost of reducing one crime; 2) the estimated cost of delivering one output (e.g. one hour of offender supervision). Please also indicate if the estimates of cost were full or partial estimates (i.e. did the review include all of the costs associated with intervention or only some of them).

1. Does the study explicitly conduct a cost-benefit analysis?
   1. Yes (specify results in the textbox)*
   2. No

* Cost benefit analysis compares the cost of implementation with the monetised benefit of the estimated outcomes (crime or reoffending reduced).

1. This form has been informed by published coding forms (e.g., Littel et al., 2008; Mazerolle, Higginson, & Eggins, in press; Mitchell, Wilson, Eggers, & MacKenzie, 2012). [↑](#footnote-ref-1)
2. The term ‘variables’ may not be used in qualitative research. Some eligible documents may utilise qualitative coding themes that can be considered variables. [↑](#footnote-ref-2)
