# Supplementary material for: Multiagency programs with police as a partner for reducing radicalisation to violence
Source: Campbell Syst Rev. 2021 May 5;17(2):e1162. doi: 10.1002/cl2.1162 (PMC8356331; doi:10.1002/cl2.1162)
Supplement: Supplementary file 4 — Supporting information [file CL2-17-e1162-s003.docx]

## Appendix D: CASP Critical Assessment Tool Guiding Questions

Table D.1: Guiding questions for ratings on the CASP Economic Evaluation Checklist

| Domain | Guiding Questions |
| --- | --- |
| Well defined question? | Is it clear what the authors are trying to achieve:   - What is the perspective; - How many options are compared; - Are both costs and consequences considered; - What is the time horizon |
| Comprehensive description of competing alternatives? | Is there a clear decisions tree (or similar given):   - Can you tell who did what, to whom, where and how often |
| Evidence for effectiveness? | Consider:   - If an RCT or systematic review was used; - If not, consider how strong the evidence was (economic evaluations frequently have to integrate different types of knowledge stemming from different study designs) |
| Effects of intervention measured and valued appropriately? | Effects can be measured in terms of:   - Natural units (e.g. years of life); - More complex units (e.g. years adjusted for quality of life such as QALYS); or - Monetary equivalents of the benefit gained (e.g. $) |
| All important and relevant resources required, and outcome costs for each alternative identified, measured in appropriate units and valued credibly? | Consider how these were identified:   - Remember the perspective being taken;   Consider if it was measured accurately:   - Are appropriate units measured accurately (may be hours of nursing time, number of physician visits, years-of-life gained etc.);   Consider if it is valued credibly:   - Are the values realistic; - How have they been derived; - Have opportunity costs been considered |
| Costs and consequences adjusted for different times (discounting)? |  |
| Incremental analysis of consequences and cost of alternatives provided? | Consider:   - What is the bottom line; - What units were used (e.g. cost/life year gained, cost/WALY, net benefit) |
| Adequate sensitivity analysis performed? | Consider:   - If all the main areas of uncertainty were considered by changing the estimate of the variable and; - looking at how this would change the result of the economic evaluation |

Table D.2: Guiding questions for ratings on the CASP Cohort Study Checklist

| Domain | Guiding Questions |
| --- | --- |
| Well Defined Question? | A question can be ‘focused’ in terms of:   - The population studied; - The risk factors studied; - Whether the study tried to detect a beneficial or harmful effect; - The outcomes considered |
| Appropriate Recruitment? | Look for selection bias which might compromise validity of the findings:   - Was the cohort representative of a defined population; - Was there something special about the cohort; - Was everybody included who should have been |
| Exposure Measured to Minimise Bias? | Look for measurement, recall or classification bias:   - Did they use subjective or objective measurements; - Do the measurements truly reflect what you want them to (have they been validated); - Were all the subjects classified into exposure groups using the same procedure |
| Outcome Measured to Minimise Bias? | Look for measurement, recall or classification bias:   - Did they use subjective or objective measurements; - Do the measurements truly reflect what you want them to (have they been validated); - Has a reliable system been established for detecting all the cases (e.g for measuring disease occurrence); - Were the measurement methods similar in the different group; - Were the subjects and/or the outcome assessor blinded to exposure (does this matter?) |
| Identification of and Account Important Confounding Factors in Design or Analysis? | Consider:   - The factors that may be important or that may have been missed; - Restriction in design and techniques e.g. modelling, stratified-, regression-, or sensitivity analysis to correct, control or adjust for confounding factors |
| Complete and Appropriate Length of Follow-Up? | Consider:   - Whether the good or bad effects should have had long enough to reveal themselves; - Whether the persons that are lost to follow-up may have different outcomes than those available for assessment; - In an open or dynamic cohort, was there anything special about the outcome of the people leaving, or the exposure of the people entering the cohort |

Table D.3: Guiding questions for ratings on the CASP Qualitative Study Checklist

| Domain | **Guiding Questions** |
| --- | --- |
| Well Defined Question? | Consider:   - What was the goal of the research; - Why it was thought important; - Its relevance |
| Qualitative Method Appropriate? | Consider:   - If the research seeks to interpret or illuminate the actions and/or subjective experiences of research participants; - Is qualitative research the right methodology for addressing the research goal |
| Research Design Appropriate for Research Aims? | Consider:   - If the researcher has justified the research design (e.g. have they discussed how they decided which method to use) |
| Appropriate Recruitment Strategy? | Consider:   - If the researcher has explained how the participants were selected; - If they explained why the participants they selected were the most appropriate to provide access to the type of knowledge sought by the study; - If there are any discussions around recruitment (e.g. why some people chose not to take part |
| Data Collection Appropriate for Research? | Consider:   - If the setting for the data collection was justified; - If it is clear how data were collected (e.g. focus group, semi-structured interview etc.); - If the researcher has justified the methods chosen; - If the researcher has made the methods explicit (e.g. for interview method, is there an indication of how interviews are conducted, or did they use a topic guide); - If methods were modified during the study. If so, has the researcher explained how and why; - If the form of data is clear (e.g. tape recordings, video material, notes etc.); - If the researcher has discussed saturation of data |
| Relationship Between Researcher and Participants Adequately Considered? | Consider:   - If the researcher critically examined their own role, potential bias and influence during (a) formulation of the research questions (b) data collection, including sample recruitment and choice of location; - How the researcher responded to events during the study and whether they considered the implications of any changes in the research design |
| Ethical Issues Taken into Consideration? | Consider:   - If there are sufficient details of how the research was explained to participants for the reader to assess whether ethical standards were maintained; - If the researcher has discussed issues raised by the study (e.g. issues around informed consent or confidentiality or how they have handled the effects of the study on the participants during and after the study); - If approval has been sought from the ethics committee |
| Rigorous Data Analysis? | Consider:   - If there is an in-depth description of the analysis process; - If thematic analysis is used. If so, is it clear how the categories/themes were derived from the data; - Whether the researcher explains how the data presented were selected from the original sample to demonstrate the analysis process; - If sufficient data are presented to support the findings; - To what extent contradictory data are taken into account; - Whether the researcher critically examined their own role, potential bias and influence during analysis and selection of data for presentation |
| Clear Statement of Findings? | Consider:   - If the findings are explicit; - If there is adequate discussion of the evidence both for and against the researcher’s arguments; - If the researcher has discussed the credibility of their findings (e.g. triangulation, respondent validation, more than one analyst); - If the findings are discussed in relation to the original research question |
